# Host–Pathogen Interactions between *Xanthomonas fragariae* and Its Host *Fragaria* × *ananassa* Investigated with a Dual RNA-Seq Analysis

**DOI:** 10.3390/microorganisms8081253

**Published:** 2020-08-18

**Authors:** Michael Gétaz, Joanna Puławska, Theo H.M. Smits, Joël F. Pothier

**Affiliations:** 1 Environmental Genomics and Systems Biology Research Group, Institute of Natural Resource Sciences, Zurich University of Applied Sciences (ZHAW), CH-8820 Wädenswil, Switzerland; michael.getaz@gmail.com (M.G.); theo.smits@zhaw.ch (T.H.S.); 2 Department of Phytopathology, Research Institute of Horticulture, 96-100 Skierniewice, Poland; joanna.pulawska@inhort.pl

**Keywords:** strawberry, plant inoculation, transcriptome, RNA-sequencing, virulence factors

## Abstract

Strawberry is economically important and widely grown, but susceptible to a large variety of phytopathogenic organisms. Among them, *Xanthomonas fragariae* is a quarantine bacterial pathogen threatening strawberry productions by causing angular leaf spots. Using whole transcriptome sequencing, the gene expression of both plant and bacteria in planta was analyzed at two time points, 12 and 29 days post inoculation, in order to compare the pathogen and host response between the stages of early visible and of well-developed symptoms. Among 28,588 known genes in strawberry and 4046 known genes in *X. fragariae* expressed at both time points, a total of 361 plant and 144 bacterial genes were significantly differentially expressed, respectively. The identified higher expressed genes in the plants were pathogen-associated molecular pattern receptors and pathogenesis-related thaumatin encoding genes, whereas the more expressed early genes were related to chloroplast metabolism as well as photosynthesis related coding genes. Most *X. fragariae* genes involved in host interaction, recognition, and pathogenesis were lower expressed at late-phase infection. This study gives a first insight into the interaction of *X. fragariae* with its host. The strawberry plant changed gene expression in order to consistently adapt its metabolism with the progression of infection.

## 1. Introduction

Plants cannot move to escape environmental challenges such as various biotic and abiotic factors throughout their life cycle. Therefore, they have developed sophisticated perception systems and polyvalent biochemical defense response mechanisms to cope with these threats [1]. Strawberry (*Fragaria* × *ananassa*) is one of the most appreciated cultivated fruits in the world owing to the pleasant flavor and nutritional content of the fruits [2,3], which makes it an economically important crop in the world. A better understanding of strawberry physiological responses at a molecular level can provide valuable information to improve future breeding strategies for new strawberry varieties and to engineer strawberry plants for durable and broad-spectrum disease resistance [4]. *Fragaria × ananassa* is a hybrid octoploid species (2n = 8*x* = 56) resulting from a spontaneous cross of two wild octoploid species, *Fragaria chiloensis* and *Fragaria virginiana* [5]. The genome size of *F. × ananassa* was estimated to be in the order of 708–720 Mb [6,7]. However, no complete genome sequence of *F.* × *ananassa* was made publicly available so far [8]. The dissection of the available genomes belonging to the *Fragaria* species led to the construction of a virtual reference genome by integrating the sequences of four homoeologous subgenomes of *F.* × *ananassa* wild relatives (*Fragaria iinumae*, *Fragaria nipponica*, *Fragaria nubicola*, and *Fragaria orientalis*), from which heterozygous regions were eliminated [9]. Recently, a study focusing on the gene expression of strawberry fruit ripening of *F.* × *ananassa* and assembling transcriptome from RNA-seq data resulted in a high sequence identity of 91.3% with the woodland strawberry *Fragaria vesca* [8]. Indeed, to date, most of the strawberry genetic research was focused on *F. vesca* because of its relatively simple diploid genome compared with *F.* × *ananassa* [10]. *F. vesca* has a small genome size (approximately 240 Mb; 2n = 2*x* = 14) [11] and its full genome sequence was publicly released [12], thus making it relevant as a reference for further genomic analyses.

*F.* × *ananassa* originates from a plant species susceptible to a large variety of phytopathogenic organisms [3,13,14,15]. One of these, *Xanthomonas fragariae*, is a Gram-negative bacterium causing angular leaf spots disease [16]. Under favorable conditions, the pathogen can cause significant damage to both plant stock and strawberry production [17]. Therefore, *X. fragariae* was listed in 1986 as an A2 quarantine pest on planting stocks within Europe by the European and Mediterranean Plant Protection Organization (EPPO) [18]. *X. fragariae* causes angular water-soaked spots appearing initially only on the abaxial leaf surface [19]. The size of the lesions increases progressively, which may lead to visible coalescent spots on the upper surface of the leaf [20]. Subsequently, the lesions spread all over the foliage and form larger necrotic spots [21]. Finally, the plants can suffer from vascular collapse [22]. However, incidence of the disease was reported to be variable between strawberry cultivars, suggesting differential sensitivity to *X. fragariae* [21]. The bacterial disease was first reported in 1960 in Minnesota, USA [16]. In 2018, a study reported that two distinct groups of strains were already separated at that time [23]. Complete reference genomes from both groups of strains are available [24,25], thus providing an ideal base for gene expression analyses. Both groups were reported as being pathogenic on strawberry and harbored similar virulence-related protein repertoires including a type III secretion system (T3SS) and its effectors (T3E), a type IV secretion system (T4SS), and a type VI secretion system (T6SS) [26].

Advances in plant–pathogen interactions are of great interest in order to understand response pathways of both plant and pathogen, and reconstruct multiscale mechanistic models incorporating plant, pathogen, and climate properties in a context of agricultural challenges for the future [27]. A metabolomics approach allows the simultaneous analysis of primary and secondary plant metabolites, both quantitatively and qualitatively, in organisms [28,29], and thus reflects changes in the level of metabolites related to biotic or abiotic stress [30]. This method was applied for naturally infected strawberries (*F.* × *ananassa*) with *X. fragariae* and revealed a reduction of some plant-defense pathways for long-term bacterial disease stress [31]. However, this technique did not allow performing a simultaneous monitoring of the bacterial activity.

DNA microarrays have been largely used to study the expression levels of transcripts in many plants including strawberry [32,33,34]. This technique could unveil a subset of genes in *Arabidopsis thaliana* responsible for both resistance and susceptibility to diseases, while the phenotype relies on the timing and magnitude of expression of those genes [35]. However, DNA microarrays have a number of limitations, providing indirect measures of relative concentrations with possible saturation or too high detection limits, and the array can only detect sequences that it was designed to detect [36]. With the advent of next-generation sequencing, high-throughput mRNA sequencing (RNA-seq) has become the major method for transcriptomic analysis, which can quantify genome-wide expression in a single assay with higher resolution and better dynamic range of detection [37]. This technique has been successfully applied to investigate differential gene expression in several pathosystems, like *Xanthomonas arboricola* pv. pruni in peach leaves [38], *Xanthomonas axonopodis* pv. glycines within soybean leaves [39], *Xanthomonas oryzae* pv. oryzae in rice varieties [40], or *Erwinia amylovora* in apple flowers [41] and apple shoots [42].

To better understand the behavior of both *X. fragariae* and *F.* × *ananassa* during its interaction, the transcriptome of both organisms was assessed using RNA-seq after artificial plant inoculation. This allows a first view on the interaction between the host plant and the pathogen.

## 2. Materials and Methods

### 2.1. Bacterial Strain and Bacterial Preparation

The type strain *X. fragariae* PD 885^T^, which contains a chromosome and two plasmids (GenBank accession numbers: LT853882—LT853884) [24], was stored in 50% glycerol at −80 °C and revived on plates containing Wilbrinks-N medium [43], 5 to 7 days before performing liquid cultures. The inoculum was prepared by growing the bacteria in liquid Wilbrinks-N medium [43] for 48 h while shaking at 220 rpm. Bacteria were collected by centrifugation and washed twice with Ringer solution (Sigma Aldrich, Buchs, Switzerland). Washed bacteria were resuspended in Ringer solution and the concentration was adjusted to 0.1 OD_600_ units (Libra S22; Biochrom, Cambridge, UK).

### 2.2. Plant Inoculation and Leaf Collection

Six strawberry plants (*F*. × *ananassa* variety Elsanta) were inoculated by spraying *X. fragariae* on the foliar part of the plants following the protocol described by Kastelein et al. [44]. The plants were placed in a plastic bag two days before and after inoculation in order to keep high relative humidity (RH) to allow opening of stomata and, therefore, to favor infection. Plants were kept for a total of 30 days post inoculation (dpi) in a climate chamber (WeissTechnik, Leicestershire, United Kingdom). Controlled conditions were set for the whole experiment with 16 h of daylight with 22 °C and a 70% RH and an 8 h nighttime with 17 °C and 80% RH. Symptoms were recorded starting from 12 dpi. Leaves were collected at 12 and 29 dpi. Three leaves per time point were collected in a sterile 50 mL tube and immediately frozen in liquid nitrogen. Storage was done at −80 °C until RNA extraction.

### 2.3. RNA Extraction from Plant Material

Total RNA (i.e., both bacterial and plant RNA) was extracted from all collected leaves. Owing to the richness in polysaccharides and phenolic compounds of strawberry plant tissues, the extraction was performed with a modified method of Christou et al. [45], as outlined below. Collected leaves were cut into three sections, used as triplicates of 100 mg initial material and extracted in parallel. The extraction buffer (EB) was supplemented with freshly added 2% β-mercaptoethanol (Applichem GmbH, Darmstadt, Germany) in order to preserve samples from RNase activity; the powdered leaves were transferred in ice-cold EB and let on ice for 15 min with shaking every 3 min, in order to allow the extraction buffer to access all plant material and avoid sedimentation of material, instead of directly adding phenol/chloroform/isoamyl alcohol (25:24:1 *v/v*; AppliChem GmbH, Darmstadt, Germany); RNA samples were washed twice with 70% (*v*/*v*) ethanol in order to remove traces of phenols and other potentially interfering components; and nucleic acid pellet was air-dried at room temperature for 2 min and subsequently dissolved in 30 µL RNase free water on ice for 15 min.

### 2.4. RNA Quantification, Qualification, and DNase Treatment

All three replicate RNA samples isolated from three plant leaves in each of the two collection days were tested for nucleic acid quantity and purity by measuring spectrophotometrically the absorbance ratios A_260_/A_230_ and A_260_/A_280_ using a Q5000 micro volume spectrophotometer (Quawell Technology, San Diego, CA, USA; Table S1).

Total RNA of replicates collected at 12 dpi and 29 dpi were treated with DNase I (Macherey-Nagel GmbH & Co., Germany) according to manufacturer’s protocol, followed by an ethanol-based RNA precipitation before resuspending the RNA in 30 µL RNase free water. Two PCR controls using primer sets previously designed to amplify housekeeping genes, namely *gyrB* in *X. fragariae* [46] and actin in woodland strawberry [47], were performed to confirm the absence of contaminating DNA. The PCR mixture consisted of 10 μL polymerase 2× KAPA2G Robust HotStart ReadyMix PCR Kit (KAPABiosystem, Wilmington, MA, USA), 10 μM forward primer, 10 μM reverse primer, 5 μL ultrapure water, and 3 μL template DNA. Amplification was performed using a Bio-Rad PCR machine, with a thermal cycle programmed for 3 min at 95 °C as initial denaturation, followed by 15 cycles of 15 s at 95 °C for denaturation, 15 s at 60 °C as annealing, 15 s at 72 °C for extension, and 1 min at 72 °C for final extension. DNase I treatment was repeated in the case of a positive amplification. The RNA integrity of extracted nucleic acids was verified by running samples after DNase treatment through a fragment analyzer (Advanced Analytical, Akeny, IA, USA) with a high sensitivity RNA analysis kit (Advanced Analytical). Only one replicate per leaf was selected for RNA sequencing (Table S1).

### 2.5. RNA Processing and Sequencing

The selected RNA samples were depleted of rRNA with both bacterial and plant Ribo-Zero rRNA Removal Kits (Illumina, San Diego, CA, USA). For each replicate, cDNA libraries were prepared by the Functional Genomics Centre Zurich (University of Zurich, Switzerland) using a TruSeq Stranded mRNA Library Prep kit (Illumina, San Diego, CA, USA). All libraries were then pooled and sequenced with 125 bp single direction reads using two lanes of an Illumina HiSeq 4000 machine. All raw sequencing reads and processed data supplementary files were deposited in NCBI Gene Expression Omnibus (GEO, https://www.ncbi.nlm.nih.gov/geo/) with accession number GSE150636.

### 2.6. Bioinformatics

Reads were trimmed with Trimmomatic v. 0.36 [48] in order to clip sequencing adapters and to remove low quality reads. Reads were subsequently mapped with Bowtie 2 v. 2.3.2 [49] separately on either the *X. fragariae* PD 885^T^ genome (GenBank assembly accession GCA_900183975.1) [24] or the *F. vesca* genome v.4.0 [12]. SAMtools v. 0.1.19 [50] was subsequently used to sort the mapped reads on their respective bacterial or plant reference genome. The sorted files of a total of six replicates, resulting from three independent leaves per collection day, were processed with the Cufflinks RNA-seq workflow v. 2.2.0 [51] in order to obtain gene and transcript expression information per replicate and per treatment, for the bacterium and the plant separately. Gene expression levels were normalized using fragments per kilobase of exon per million mapped reads (FPKM) report values. The outputs were analyzed and visualized on the package cummeRbund v. 2.20.0 [52] in R v. 3.4.3 [53]. The replicates were controlled for reproducibility using a principal component analysis (PCA), and in the case of an outlier replicate, the Cufflinks workflow was repeated after removing the outlier replicate. Genes were considered as significantly differentially expressed, when their fold change (Log_2_) between 12 dpi and 29 dpi was ≥1.5 or ≤−1.5, respectively, and their adjusted *p* value< 0.05. For each differentially expressed bacterial gene, the gene annotation from the reference genome PD 885^T^ was assigned, and gene ontology (GO) categorization was subsequently added with Blast2Go [54]. Additionally, virulence-related genes in *X. fragariae*, such as T3SS, T3E, T4SS, and T6SS, retrieved from the annotated genome PD 885^T^ [26], were specifically screened for expression levels for both collection days and compared with housekeeping genes.

For each differentially expressed plant gene, gene functions for *F. vesca* were obtained using ad hoc Perl scripts to combine GO, InterProScan (IPR), KEGG orthologues, and pathways, as well as BLAST information obtained from the Genome Database for *Rosaceae* (GRD, URL www.rosaceae.org).

## 3. Results and Discussion

### 3.1. Sequenced RNA Reads Selection

Sequencing of the different RNA samples yielded between 39 million and 149 million reads per sample (Table 1). Subsequent filtering removed between 2.6% and 11.0% of low-quality reads.

**Figure 1:**
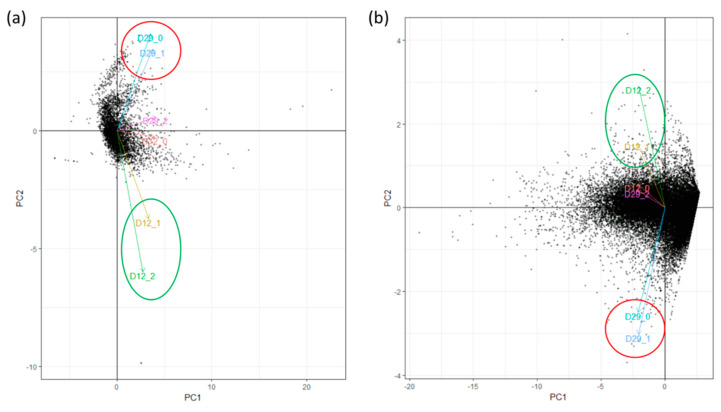
The principle component analysis (PCA) performed with the CummeRbund workflow on differentially expressed genes for (**a**) *Xanthomonas fragariae* and (**b**) *Fragaria × ananassa*. Three leaf replicates at 12 days post inoculation (dpi) (D12_0, D12_1, D12_2) and three leaf replicate at 29 dpi (D29_0, D29_1, D29_2) were analyzed with principle component for both bacteria and plant and the arrows represent the most-varying direction of the data.

Mapping of the remaining reads on the *X. fragariae* genome yielded between 1.23 and 4.81 million mapped reads, which represented 2.58% to 8.51% of the filtered reads. The read mapping on the *F. vesca* genome yielded between 32.63 and 109.97 million mapped reads, representing between 83.44% and 90.7% of the filtered reads (Table 1). On the basis of PCA analysis, one sample per collection day was defined as being an outlier (Figure 1a,b), with two replicates remaining per collection day for both bacterial and plant analysis.

### 3.2. Gene Expression in *X. Fragariae*

**Figure 2:**
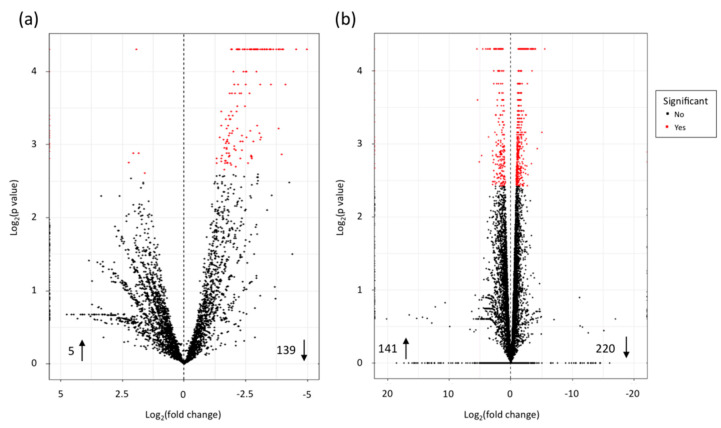
Volcano plots representing all expressed transcripts. For every transcript, the fold change of 12 days post inoculation (dpi) and 29 dpi was plotted against the *p*-value for both (**a**) *Xanthomonas fragariae* and (**b**) *Fragaria × ananassa*. Statistically significant differentially expressed genes, with a Log_2_ fold change ≥1.5 or ≤−1.5, are depicted as a red dot, and insignificant as black dots. For each organism, the numbers aside the arrows pointing up represent the number of higher expressed genes and the numbers aside arrows pointing down represent the number of lower expressed genes.

A total of five bacterial genes were more expressed at the later sampling point (Figure 2a; Table S2).

Among them, a single calcium-binding gene, also annotated as putative RTX related-toxin, was found (Table 2). Hemolytic and cytolytic RTX-toxins are reported to be pathogenicity factors of the toxin-producing bacteria and are very often important key factors in pathogenesis of the bacteria [55]. This suggests that *X. fragariae* may still have an active factor of pathogenesis at a late stage of the symptom expansion.

Among the resulting 139 higher expressed genes at early infection stage, the functions of some genes were related to different virulence-related systems as well as proteins involved in host interactions, recognition, and pathogenesis. Three structural elements of the T3SS were identified. HrcC and HrcJ are constitutive membrane elements of the T3SS, forming the outer and inner rings of the T3SS, respectively [56]. HrcU interacts with T3SS substrate specificity switch (T3S4) proteins including HrpB and was proposed to control the secretion of different T3S substrate classes by independent mechanisms [57]. One regulatory gene of the T3SS, *hrpB* (hypersensitive response and pathogenicity), was more expressed at 12 dpi and is reported to regulate transcriptional control of the T3SS [58]. This transcription factor is an expression activator of the T3SS encoding genes and T3E genes [59]. Two additional T3SS regulation factors, *hpa1* and *hpaB* (hypersensitive response and pathogenicity associated), reported to influence virulence with the host [58,60], were found to be more expressed at 12 dpi. While comparing with the change of expression of these genes between bacteria growing on microbiological medium and in planta, expression of all of them was significantly higher in strawberry plants 15 days after inoculation, which confirms that the T3SS is important in the early stage of infection [61]. Finally, three T3E genes, namely, *xopN*, *xopR*, and *xopV*, were more expressed at the early infection stage, suggesting their translocation into the host cell, thus contributing to virulence by suppressing innate immune response in strawberry [62]. Furthermore, a gene belonging to the T4SS pilus, *pilQ*, for which its gene product was reported to play a crucial role in pathogenicity, twitching motility, and biofilm formation in *Xanthomonas* species [63,64,65], was more expressed at the early symptom stage, similarly to on microbiological medium than in planta [61]. Three elements from the T6SS were higher expressed at 12 dpi as well. The needle protein Hcp forms the tubular structure that is secreted out of the cell [66], whereas the VgrG protein was reported as an indispensable component for the specific delivery of effectors and acting as a puncturing device [67]. The membrane element EvpB, homologous to TssB [68,69], forms a sheath that wraps around the Hcp tube and dynamically propels the Hcp-VgrG puncturing device and T6SS effector across the bacterial membrane [70,71]. In general, T6SS have mainly been shown to contribute to pathogenicity and competition between bacteria [72]. The presented results suggest that both T3SS and T6SS are more active at 12 dpi and may secrete effectors for both systems. The differentially expressed genes from T3SS, T4SS pilus, and T6SS may thus be good candidate targets for mutational analysis in *X. fragariae* in order to test their role in virulence as they could constitute key virulence factors, and thus reveal weakness of the bacterium if silenced.

**Figure 3:**
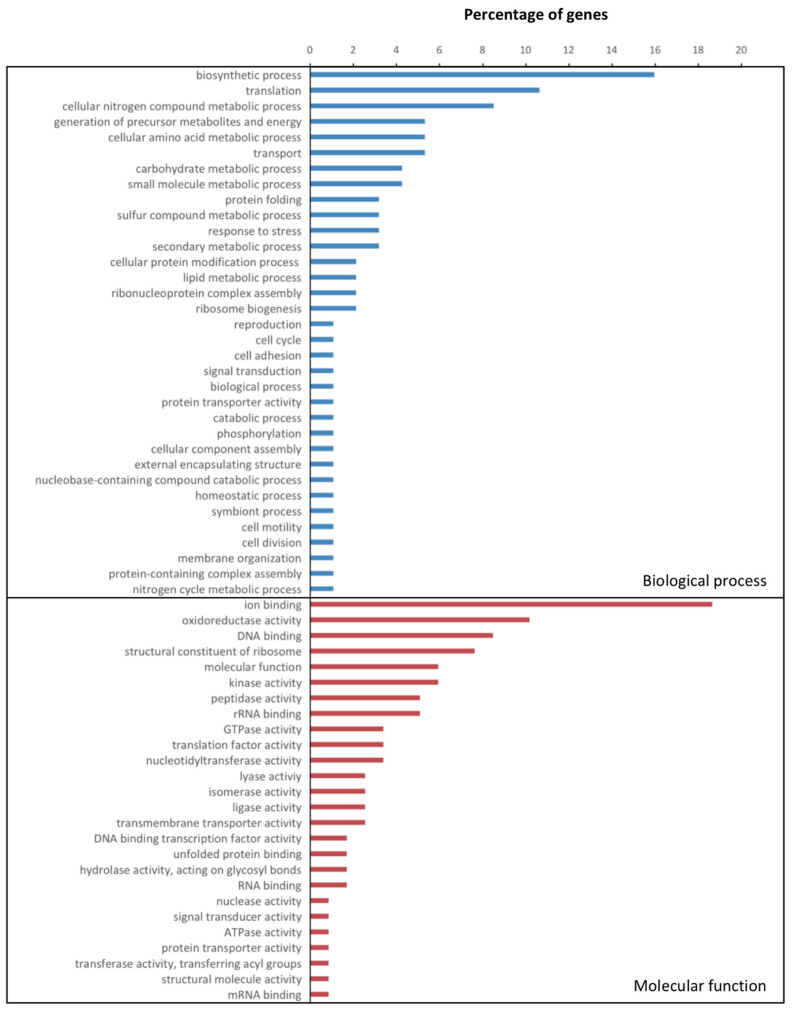
Gene ontology (GO) categories less expressed at 29 days post inoculation (dpi) in *Xanthomonas fragariae***.** Two classes of GO terms, namely biological process and molecular functions in inoculated strawberry plants between 12 and 29 dpi, are shown as a percentage of present genes.

The genes for other factors such as chaperonin GroEL, known as a common antigen and effecting the innate and acquired immune systems [73], glutamate synthetase *glnA*, which was shown to contribute to the virulence in *Streptococcus suis* [74], bacterial recognition, and interaction-related genes, such as a leucine-rich protein, putatively involved in bacterial surface recognition [75], and avirulence factors in host tissue [76], were more expressed at an early infection stage. Subsequently, a total of eight genes related to ribosomal functions in 30S and 50S were found, which, together with the previous set of genes, would suggest a faster growth rate at the early infection stage [77]. The GO annotation for biological process congruently showed that biosynthetic process, translation, metabolic process, and generation of precursor metabolites were more expressed at 12 dpi (Figure 3).

Further higher-expressed genes at an early stage of infection were coding for the membrane proteins OmpA and OmpW, which may favor bacterial pathogenesis by anchoring the host cell [78,79]. They may be involved in biofilm formation [80], similarly to proteins responsible for lipopolysaccharide (LPS) also highly expressed at 12 dpi, and assemble at the cell surface [81,82]. Biofilms facilitate adhesion of the colonization to both biotic and abiotic surfaces, thus allowing the bacteria to resist physical stresses imposed by fluid movement that could separate the cells from a nutrient source and increasing bacterial fitness in the plant [83]. On the basis of the transporter classification database (TCDB) [84], both bacterial TonB-dependent receptors (TBDRs), which were more expressed at an early infection stage, were found to be involved in iron (Fe^3+^) binding and transport. There is evidence that phytopathogenic bacteria can use iron uptake systems to multiply in the host and to promote infection [85]. A study could already report that iron acquisition was crucial for *X. fragariae* bacterial growth because an iron deprivation could inhibit *X. fragariae* growth and symptoms on strawberry plant [86].

Overall, the higher expressed bacterial genes at 12 dpi would suggest that the bacteria were more actively growing in the plant leaf compared with 29 dpi. At this time point, expression of the pathogenicity factors was higher. At the later time point, growth of the bacterium was reduced. The growth limitation and bacterial metabolism change could be explained by an effective bacterial recognition by the plant and a deprivation of nutrients in the leaf by the reduction of the photosynthesis process in the leaf (see below), thus limiting the access of nutrients for the bacteria. However, the collection time at 12 dpi also coincides with the preparatory stage of the bacteria before the exudation phase, which usually starts at 14 dpi [44].

Additionally, the lower expression of virulence-related genes at a later infection stage could reflect that *X. fragariae* appears rather to be a biotrophic pathogen [87]. The reduced cell wall degrading enzyme (CWDE) repertoire, as reported from the draft genome of *X. fragariae* in comparison with other *Xanthomonas* spp., typically found in biotrophic pathogens [87,88], would only support this hypothesis. However, the T3SS in addition to defense suppression may also have induced cell death (see below), thus indicating a hemibiotrophic life style [89]. In fact, phytopathogenic bacteria should be seen as a continuum of hemibiotrophs owing to the different life style phases occurring during plant–bacterial interactions [89].

### 3.3. Gene Expression in Strawberry

The analysis of RNA-seq data indicated that a total of 141 genes were more expressed at the later sampling point (29 dpi), while 220 genes were more expressed at the early infection stage (Figure 2b; Table S3). Some pathways were shown to be partly more expressed at an early stage, while some elements of the same pathways were more expressed at a late stage of infection (Table 3).

Among these pathways are genes with functions generally related to an unspecific response to biotic and abiotic stimuli, including glutathione metabolism [90] and cytochromes (mainly P450) [91]. Glutathione may affect the levels of reactive oxygen species (ROS) in the cell, and thus participate in the hypersensitive reaction (HR) launched by resistant plants following pathogen attack [92,93,94]. This could explain why the used cultivar was not considered as highly susceptible to *X. fragariae* [21]. Cytochrome P450 genes, which are involved in plant development, antioxidant, and detoxification of pollutants, are also involved in plant defense by protecting from various biotic and abiotic stresses [91,95]. Leucine-rich repeat (LRR) regions proteins were described as a part of the mechanism leading to recognition of pathogen and activation of signal pathways related to plant defense and disease resistance [96,97]; they are associated with the innate immune response, which is initiated through the sensing of pathogen-associated molecular patterns (PAMPs) [98]. Additionally, genes coding for proteins functioning as phytohormones such as auxin (AAI) and ethylene (ET), which are known to be key mediators of plant responses to both biotic and abiotic stresses [99,100,101,102], may be involved in senescence processes depending on concentrations [103]. Overall, this suggests that the listed pathways of recognitions and defense may have a differential and a long-action spectrum along the symptom expansion.

Among the down-regulated genes at a later infection stage (Table 3), a total of 54 genes were found to be located in the chloroplast: 9 of them were related to both photosystems I and II, 14 of them to chlorophyll A/B binding, 4 of them to plastid-lipid-associated proteins, and 6 were related to gluconeogenesis or citric acid cycle shunt and other functions. The chloroplast was reported to play a major role in plant immunity by hosting biosynthesis of several key defense-related molecules, such as hormones and secondary messenger [104,105,106]. A down-regulation of the light harvesting complexes and protein related to chlorophyll A/B was already reported in the reaction of peach plants to the pathogen *X. arboricola* pv. pruni [38], of kumquat as reaction to *Xanthomonas citri* subsp. citri [107] and of *Arabidopsis thaliana* to *Pseudomonas syringae* [108]. It was concluded that the down-regulation of the genes involved in photosynthesis was a cost for the plant fitness, where energy resources were redirected to defense response. This could induce a hypersensitive response following the infection [107]. A recent study showed that T3E from *P. syringae* could target the chloroplasts from *A. thaliana* and disrupt the photosystem II, leading to an inhibition of the photosynthesis, thus decreasing the PAMPs-induced reactive oxygen species (ROS) production [105]. Alternatively, in the case of bacterial infections, several reports have shown a suppression of photosynthetic functions in infected plants, possibly reflecting an active plant response to shut down carbon availability and limit pathogen growth, in order to favor the establishment of defense over other physiological processes [104,109] or to protect the photosynthetic apparatus against oxidative damage [110].

Among the more expressed genes at a late infection stage, four were involved in specific plant defenses regulation, such as WRKY transcription factors [111,112], which are described as part of the mechanism leading to recognition of pathogen and activation of signal pathways related to plant defense and disease resistance [96,97]. NAC domain containing proteins were also more expressed at a late stage of infection and the plant-specific NAC domain containing protein family controls processes such as development, defense, and abiotic stress responses [113]. A total of 16 genes coding for other pathogenesis-related factors were mostly more expressed at a late stage. Among them, two coding genes for beta-1,3-glucanase, three chitinases, three thaumatin-like proteins, and four genes coding for a glucan endo-1,3-glucosidase protein were found. Genes coding for beta-1,3-glucanase and chitinase were found to be involved in the reaction to symptomatic bacterial spots on tomato [114], while genes coding for thaumatin-like proteins and glucan endo-1,3-glucosidase proteins could play a role in plant defense against bacterial diseases [115,116].

**Figure 4:**
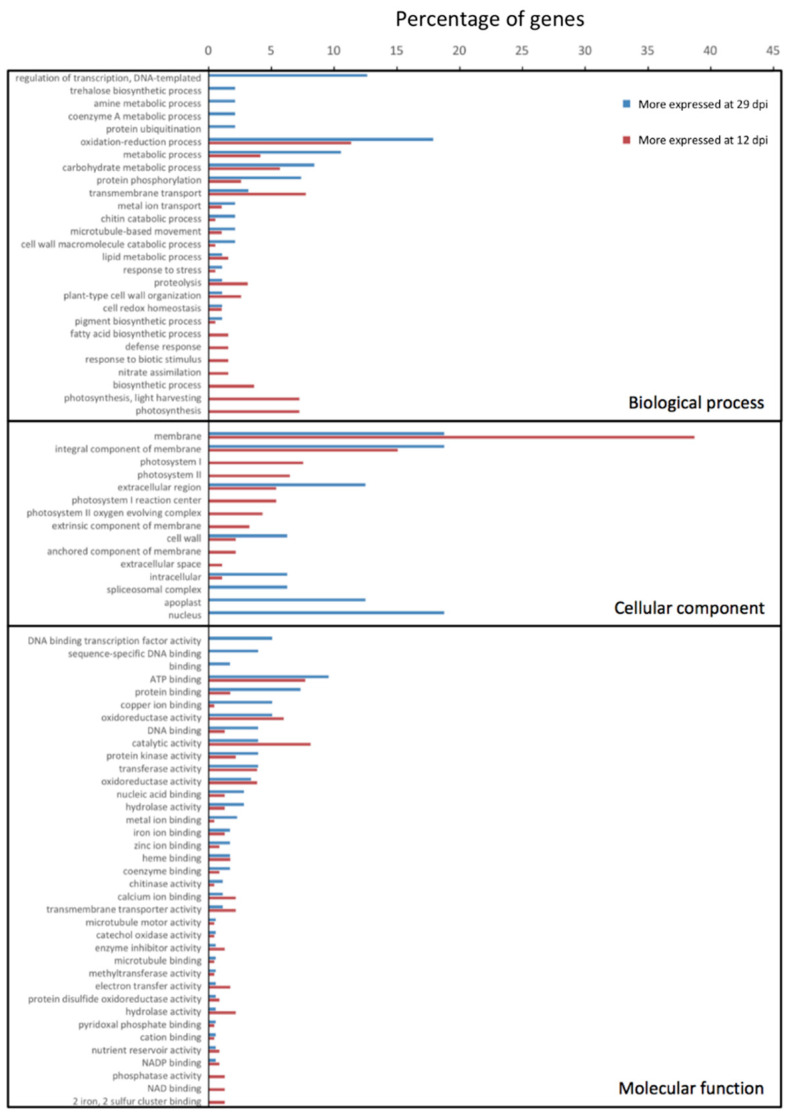
Gene ontology (GO) categories differentially expressed between 12 and 29 days post inoculation (dpi) in *Fragaria* × *ananassa*. The most represented categories from all three classes of GO annotations (i.e., biological process, cellular component, molecular function) are represented as a percentage of genes per categories.

Overall, complementary to the presented results, the GO annotation revealed that the biological processes from genes more expressed at 12 dpi were related to both photosystems I and II, metabolic processes, and transmembrane transports, as well as to defense response and response to biotic stimulus (Figure 4). This may reflect that defense mechanisms of the strawberry plant were already activated by the pathogen at 12 dpi, but that the process already declined at 29 dpi. The results at an early infection point suggest a change in plant defense strategy metabolism by changing mostly its chloroplast metabolism, and thus removing access to nutrients, favoring bacterial growth and possibly inducing cell death. Additionally, basal plant defense may already be activated at an early stage, but bacterial recognition may only be effective at a later infection stage.

## 4. Conclusions

The analysis of the interaction of *X*. *fragariae* and *F*. × *ananassa* using RNA-seq technology enhances our understanding of the genetics underlying the interaction mechanisms in this pathosystem. This study gives a global view of the gene expression of both the pathogen and host of the bacterial disease development caused by *X*. *fragariae* on strawberries. Moreover, the present study could explore the gene expression of *F*. × *ananassa* with a more complete picture than a previous study on metabolomics of strawberry plants infected with *X*. *fragariae* that could only focus on 28 compounds in strawberry leaves [31]. Although in this study, the used strawberry cultivar was not considered as highly susceptible to *X*. *fragariae* [21], we were able to show differences between the plant defense strategy and bacterial colonization at two selected time points.

## Figures and Tables

**Table 1:** Raw reads produced from RNA sequencing per replicate, retained reads resulted from RNA trimming. Reads were mapped on both *Xanthomonas fragariae* PD 885^T^ (GenBank assembly accession GCA_900183975.1) and *Fragaria vesca* (v. 4.0) genomes. Mapping results provided the number and percentage of reads uniquely mapped to the genome and number and percentage of reads mapped more than one time to the respective genome. Finally, the overall aligned amount and percentage of reads mapped on each genome were reported in the table. Dpi: days post inoculation.

Replicate	Raw Reads	Trimming and Filtering		Bacterial Mapping		Plant Mapping	
		Remaining Reads	Removed Reads (%)	Overall Aligned	Overall Aligned (%)	Overall Aligned	Overall Aligned (%)
12 dpi leaf 1 ^1^	65,512,500	56,513,044	13.74	4,806,523	8.51	39,162,615	69.30
12 dpi leaf 2	64,973,090	61,741,330	4.97	1,708,033	2.77	54,919,192	88.95
12 dpi leaf 3	44,154,658	41,413,993	6.21	1,235,070	2.98	37,562,210	90.70
29 dpi leaf 1	39,031,270	38,021,945	2.59	2,776,597	7.30	32,632,204	85.82
29 dpi leaf 2	79,106,667	70,440,561	10.95	3,101,000	4.40	58,772,409	83.44
29 dpi leaf 3 ^1^	149,738,897	143,962,456	3.86	3,711,553	2.58	109,970,724	76.39

^1^ These two replicates were removed from the analysis.

**Table 2:** Summary table of selected differentially expressed *Xanthomonas fragariae* genes while interacting in planta with *Fragaria × ananassa*. A complete list of differentially expressed genes is provided in Table S2. LPS, lipopolysaccharide.

Locus Tag	Locus: Position	Expression	Fold Change (Log_2_)	Protein Description
**Ribosome**				
PD885_RS14555	NZ_LT853882.1: 3129676–3130403	down	−1.82	30S ribosomal protein S5
PD885_RS09535	NZ_LT853882.1: 2044105–2045791	down	−2.01	30S ribosomal protein S1
PD885_RS14575	NZ_LT853882.1: 3132158–3132464	down	−3.53	30S ribosomal protein S14
PD885_RS14625	NZ_LT853882.1: 3136013–3136841	down	−2.05	50S ribosomal protein L2
PD885_RS01580	NZ_LT853882.1: 348309–348738	down	−2.15	50S ribosomal protein L13
PD885_RS14700	NZ_LT853882.1: 3154771–3155200	down	−2.28	50S ribosomal protein L11
PD885_RS14580	NZ_LT853882.1: 3132482–3133025	down	−2.52	50S ribosomal protein L5
PD885_RS04680	NZ_LT853882.1: 1026045–1026366	down	−2.70	50S ribosomal protein L21
PD885_RS14680	NZ_LT853882.1: 3152571–3152937	down	−3.07	50S ribosomal protein L7/L12
**T3SS**				
PD885_RS06675	NZ_LT853882.1: 1447891–1449712	down	−2.73	EscC/YscC/HrcC type III secretion system outer membrane ring
PD885_RS06645	NZ_LT853882.1: 1442977–1443742	down	−2.65	EscJ/YscJ/HrcJ type III secretion inner membrane ring
PD885_RS06630	NZ_LT853882.1: 1440799–1441873	down	−2.24	EscU/YscU/HrcU type III secretion system export apparatus switch
PD885_RS06635	NZ_LT853882.1: 1442090–1442546	down	−2.81	HrpB1 family type III secretion system apparatus
PD885_RS06580	NZ_LT853882.1: 1433397–1433868	down	−3.99	type III secretion protein HpaB
PD885_RS06680	NZ_LT853882.1: 1449789–1450173	down	−4.98	type III secretion protein Hpa1
PD885_RS06640	NZ_LT853882.1: 1442583–1442976	down	−2.54	type III secretion protein HrpB2
**T3E**				
PD885_RS01740	NZ_LT853882.1: 376677–378864	down	−2.39	type III effector XopN
PD885_RS02910	NZ_LT853882.1: 653223–653931	down	−2.97	type III effector XopR
PD885_RS17340	NZ_LT853882.1: 3731049–3732024	down	−1.89	type III effector XopV
**T4SS**				
PD885_RS16190	NZ_LT853882.1: 3471918–3473817	down	−1.90	type IV pilus secretin PilQ family protein–fimbrial assembly
**T6SS**				
PD885_RS10450	NZ_LT853882.1: 2231241–2232738	down	−1.65	type VI secretion system contractile sheath large subunit EvpB
PD885_RS10445	NZ_LT853882.1: 2230609–2231107	down	−3.63	type VI secretion system tube protein Hcp
PD885_RS04345	NZ_LT853882.1: 944106–946857	down	−1.72	type VI secretion system tip protein VgrG
**Chaperonin**				
PD885_RS02005	NZ_LT853882.1: 442628–444269	down	−1.50	molecule chaperonin GroEL
**Regulation**				
PD885_RS00915	NZ_LT853882.1: 215236–216646	down	−1.60	type I glutamate–ammonia ligase–glutamine synthetase GlnA
**LPS**				
PD885_RS15075	NZ_LT853882.1: 3219172–3222999	down	−1.81	LPS–assembly protein LptD–organic solvent tolerance protein
**Biofilm, membrane**				
PD885_RS13005	NZ_LT853882.1: 2801740–2802466	down	−1.75	OmpA family protein–cell envelope biogenesis protein
PD885_RS03590	NZ_LT853882.1: 788222–788894	down	−1.98	OmpW family protein–membrane protein
**TonB**				
PD885_RS16700	NZ_LT853882.1: 3587957–3590420	down	−1.83	TonB-dependent receptor (TCDB: 1.B.14.1.28)
PD885_RS16470	NZ_LT853882.1: 3524801–3527693	down	−2.02	TonB-dependent receptor (TCDB: 1.B.14.6.11)
**General stress**				
PD885_RS10575	NZ_LT853882.1: 2269375–2269633	down	−1.92	stress-induced protein
PD885_RS12550	NZ_LT853882.1: 2705902–2706391	down	−1.95	general stress protein
**Recognition**				
PD885_RS17775	NZ_LT853882.1: 3832365–3832962	down	−3.25	Ax21 family protein
**Motility**				
PD885_RS10885	NZ_LT853882.1: 2338459–2339659	down	−3.34	flagellin
**Toxin**				
PD885_RS16725	NZ_LT853882.1: 3595055–3603270	up	1.93	calcium-binding protein, Ca^2+^ binding protein, RTX toxin-related

**Table 3:** Summary table of selected differentially expressed *Fragaria × ananassa* genes challenged with *Xanthomonas fragariae*. A complete list of differentially expressed genes is provided in Table S3.

Locus Tag	Locus: Position	Expression	Fold Change (Log_2_)	Gene Description
**Glutathione metabolism**				
FvH4_4g13000	Fvb4: 16653443–16654859	up	2.45	crocetin glucosyltransferase, chloroplastic-like
FvH4_5g05100	Fvb5: 2978458–2983365	up	2.04	probable alpha,alpha-trehalose-phosphate synthase
FvH4_4g09780	Fvb4: 11758877–11762248	up	1.81	probable alpha,alpha-trehalose-phosphate synthase [UDP-forming]
FvH4_2g40150	Fvb2: 28671382–28672822	up	1.60	anthocyanidin 3-O-glucosyltransferase 5-like
FvH4_7g22820	Fvb7: 17936656–17943623	up	1.60	crocetin glucosyltransferase, chloroplastic-like
FvH4_3g29980	Fvb3: 23159280–23164945	down	−1.78	glucomannan 4-beta-mannosyltransferase 2
FvH4_6g53560	Fvb6: 39232986–39237091	down	−2.23	ribonucleoside-diphosphate reductase small chain
FvH4_7g31450	Fvb7: 22725705–22729890	down	−2.42	starch synthase 1, chloroplastic/amyloplastic
FvH4_1g12090	Fvb1: 6609415–6610712	down	−4.00	glyoxalase/fosfomycin resistance/dioxygenase domain
**Cytochrome**				
FvH4_4g29810	Fvb4: 29777129–29779171	up	2.55	cytochrome p450 78A5
FvH4_2g40560	Fvb2: 28894033–28900936	up	1.55	cytochrome p450, family 82, subfamily C, polypeptide 4
FvH4_2g07410	Fvb2: 6119730–6121188	up	1.55	allene oxide synthase-like
FvH4_5g27150	Fvb5: 18417464–18422984	down	−1.87	ferric reduction oxidase 7, chloroplastic
FvH4_5g02700	Fvb5: 1623401–1625033	down	−1.98	cytochrome p450 86A7
FvH4_5g14010	Fvb5: 7931662–7935314	down	−2.05	flavonoid 3’-monooxygenase
**Auxin (AAI)**				
FvH4_2g04750	Fvb2: 3685624–3688124	up	2.09	probable indole-3-acetic acid-amido synthetase GH3.1
FvH4_7g17340	Fvb7: 14759798–14760392	down	−1.81	auxin-induced protein X15-like
FvH4_6g44990	Fvb6: 34565510–34570206	down	−2.01	probable indole-3-acetic acid-amido synthetase GH3.5
FvH4_6g00660	Fvb6: 378744–381847	down	−2.32	putative auxin efflux carrier component 8
FvH4_6g34740	Fvb6: 27411186–27411858	down	−2.65	auxin-binding protein ABP19a
**Ethylene (ET)**				
FvH4_5g19800	Fvb5: 11637731–11638778	up	1.51	ethylene-responsive transcription factor 5
FvH4_5g38040	Fvb5: 28094328–28096045	up	2.76	aminocyclopropane-1-carboxylate oxidase homolog
FvH4_6g08370	Fvb6: 4946527–4949032	down	−1.71	S-adenosylmethionine synthase 1-like
FvH4_4g21340	Fvb4: 24380885–24383481	down	−2.15	S-adenosylmethionine synthase 2
**Leucin-rich repeat (LRR)**				
FvH4_5g24920	Fvb5: 16382894–16383420	up	2.20	putative F-box/lrr-repeat protein 23
FvH4_3g45520	Fvb3: 37735078–37737977	up	2.16	leucine-rich repeat receptor protein kinase EXS-like
FvH4_7g14060	Fvb7: 12491034–12492810	up	1.87	probable leucine-rich repeat receptor-like protein kinase At1g35710
FvH4_5g23420	Fvb5: 14763405–14766264	up	2.39	disease resistance protein RPM1-like (LRR superfamily)
FvH4_7g24240	Fvb7: 18726677–18731259	down	−1.69	probable lrr receptor-like serine/threonine-protein kinase At3g47570
FvH4_2g05530	Fvb2: 4568048–4570195	down	−1.97	leucine-rich repeat (lrr) family protein
**WRKY domain containing protein**				
FvH4_5g04360	Fvb5: 2573220–2577327	up	2.75	probable wrky transcription factor 53
FvH4_4g06830	Fvb4: 6132454–6133929	up	1.98	probable wrky transcription factor 11
FvH4_6g10510	Fvb6: 6310957–6313581	up	1.87	probable wrky transcription factor 33
FvH4_2g41060	Fvb2: 29128088–29130611	up	1.62	probable wrky transcription factor 40 isoform X2
**NAC domain containing protein**				
FvH4_4g31070	Fvb4: 30387328–30388714	up	3.29	NAC transcription factor 29-like
FvH4_2g16180	Fvb2: 14147225–14149397	up	1.83	NAC transcription factor 29
FvH4_3g20690	Fvb3: 13746269–13748147	up	1.80	NAC domain-containing protein 72-like
**Pathogenesis-related**				
FvH4_4g30150	Fvb4: 29928212–29930748	up	5.07	beta-1,3-glucanase
FvH4_6g45580	Fvb6: 34959190–34962068	up	1.94	probable endo-1,3(4)-beta-glucanase
FvH4_4g10610	Fvb4: 14349186–14350693	up	4.74	chitinase 4-like
FvH4_1g10600	Fvb1: 5814344–5815342	up	2.47	endochitinase-like protein
FvH4_4g11930	Fvb4: 15646302–15649061	down	−1.80	chitinase-like protein 1
FvH4_6g16950	Fvb6: 10815316–10816828	up	5.51	thaumatin-like
FvH4_5g01820	Fvb5: 1151603–1152293	up	4.12	thaumatin, protein P21-like
FvH4_6g24670	Fvb6: 18708864–18710041	up	2.76	thaumatin-like protein 1b
FvH4_3g28370	Fvb3: 21335348–21337404	up	4.57	glucan endo-1,3-beta-glucosidase-like
FvH4_5g06210	Fvb5: 3658609–3660218	up	3.76	glucan endo-1,3-beta-glucosidase, basic isoform-like
FvH4_6g24680	Fvb6: 18714133–18715667	up	2.28	glucan endo-1,3-beta-glucosidase, basic isoform-like
FvH4_2g02860	Fvb2: 2250275–2250770	up	2.81	pathogenesis-related protein 1A-like (cysteine-rich)
FvH4_3g02840	Fvb3: 1482707–1497385	up	2.15	cysteine-rich receptor-like protein kinase 10
FvH4_6g09980	Fvb6: 5928404–5929569	down	−1.55	non-specific lipid-transfer protein 1-like isoform X1
FvH4_6g09970	Fvb6: 5915102–5916203	down	−2.24	lipid transfer protein 4
FvH4_2g28920	Fvb2: 22545044–22545446	down	−2.84	14 kDa proline-rich protein DC2.15-like, lipip transfer
**Photosynthesis/Chloroplastic/Carbon fixation/Glyconeogenesis/Citric acid cycle shung**				
FvH4_3g21020	Fvb3: 14037513–14039386	down	−3.13	chlorophyll a-b binding protein 13, chloroplastic
FvH4_6g40970	Fvb6: 32372483–32373647	down	−2.59	chlorophyll a-b binding protein 151, chloroplastic
FvH4_6g41050	Fvb6: 32391614–32398766	down	−2.00	chlorophyll a-b binding protein 151, chloroplastic-like, partial
FvH4_7g19750	Fvb7: 16227980–16230030	down	−1.91	chlorophyll a-b binding protein 6, chloroplastic
FvH4_6g40150	Fvb6: 31710858–31712682	down	−2.02	chlorophyll a-b binding protein 8, chloroplastic
FvH4_5g30940	Fvb5: 21867161–21868613	down	−2.40	chlorophyll a-b binding protein CP24 10A, chloroplastic
FvH4_7g24350	Fvb7: 18809164–18811045	down	−2.52	chlorophyll a-b binding protein CP29.3, chloroplastic isoform X1
FvH4_6g38390	Fvb6: 30344332–30345143	down	−2.75	chlorophyll a-b binding protein of LHCII type 1
FvH4_6g32440	Fvb6: 25477938–25478742	down	−1.91	chlorophyll a-b binding protein of LHCII type 1-like
FvH4_3g06120	Fvb3: 3521880–3529614	down	−2.34	chlorophyll a-b binding protein of LHCII type 1-like
FvH4_6g38450	Fvb6: 30386770–30387574	down	−2.46	chlorophyll a-b binding protein of LHCII type 1-like
FvH4_3g37660	Fvb3: 32272449–32273253	down	−2.51	chlorophyll a-b binding protein of LHCII type 1-like
FvH4_1g09040	Fvb1: 4778659–4780612	down	−1.55	chlorophyll a-b binding protein, chloroplastic
FvH4_4g23750	Fvb4: 26130750–26132548	down	−1.68	chlorophyll a-b binding protein, chloroplastic
FvH4_6g44370	Fvb6: 34191144–34193039	down	−1.56	cytochrome b6-f complex iron-sulfur subunit, chloroplastic
FvH4_2g13890	Fvb2: 12167935–12172009	down	−1.68	fructose-1,6-bisphosphatase, cytosolic
FvH4_2g10390	Fvb2: 9250051–9252469	down	−1.74	fructose-bisphosphate aldolase 1, chloroplastic
FvH4_4g25450	Fvb4: 27213930–27219353	down	−1.71	glutamate-glyoxylate aminotransferase 2
FvH4_6g54460	Fvb6: 39756571–39759126	down	−1.52	glyceraldehyde-3-phosphate dehydrogenase A, chloroplastic
FvH4_5g25760	Fvb5: 17250900–17253991	down	−1.65	glyceraldehyde-3-phosphate dehydrogenase B, chloroplastic
FvH4_2g02490	Fvb2: 1986822–1989446	down	−1.97	malate dehydrogenase, glyoxysomal isoform X2
FvH4_6g38900	Fvb6: 30775176–30776861	down	−1.85	oxygen-evolving enhancer protein 2, chloroplastic
FvH4_3g02920	Fvb3: 1561440–1563015	down	−1.73	oxygen-evolving enhancer protein 3–2, chloroplastic
FvH4_5g33740	Fvb5: 24430492–24436620	down	−1.92	phosphoenolpyruvate carboxykinase [ATP]
FvH4_1g21630	Fvb1: 13591226–13595458	down	−1.69	photosynthetic NDH subunit of lumenal location 4, chloroplastic
FvH4_4g15260	Fvb4: 18876811–18877429	down	−1.71	photosystem I reaction center subunit II, chloroplastic-like
FvH4_3g11800	Fvb3: 6971526–6972286	down	−1.73	photosystem I reaction center subunit III, chloroplastic
FvH4_3g09680	Fvb3: 5629058–5631096	down	−2.00	photosystem I reaction center subunit psaK, chloroplastic
FvH4_3g41620	Fvb3: 34939645–34940283	down	−2.06	photosystem I reaction center subunit V, chloroplastic
FvH4_6g31740	Fvb6: 24848099–24849503	down	−1.54	photosystem I reaction center subunit VI, chloroplastic-like
FvH4_6g00530	Fvb6: 323097–325385	down	−1.68	photosystem I reaction center subunit XI, chloroplastic
FvH4_2g26970	Fvb2: 21549577–21552377	down	−2.05	photosystem II 22 kDa protein, chloroplastic
FvH4_2g31210	Fvb2: 23984136–23987486	down	−2.17	photosystem II PsbX
FvH4_2g20470	Fvb2: 17180656–17182221	down	−1.57	photosystem II reaction center Psb28 protein
FvH4_1g08270	Fvb1: 4379754–4380126	down	−2.13	photosystem II protein
FvH4_2g14790	Fvb2: 13006655–13015170	down	−1.55	probable glucuronosyltransferase
FvH4_1g24360	Fvb1: 16228411–16233750	down	−1.77	probable polygalacturonase
FvH4_4g16670	Fvb4: 20537377–20543743	down	−1.58	pyruvate, phosphate dikinase 2
FvH4_3g15380	Fvb3: 9556723–9560275	down	−1.76	sedoheptulose-1,7-bisphosphatase, chloroplastic-like

## Supplementary Materials

The following are available online at https://www.mdpi.com/2076-2607/8/8/1253/s1, Supplemental File 1 containing: Figure S1: Symptomatic strawberry leaves; Table S1: RNA quantity and quality for each leaf replicates; Table S2: Differentially expressed genes of Xanthomonas fragariae; Table S3: Differentially expressed genes of Fragaria × ananassa.
