# Supplementary material for: Host–Pathogen Interactions between Xanthomonas fragariae and Its Host Fragaria × ananassa Investigated with a Dual RNA-Seq Analysis
Source: Microorganisms. 2020 Aug 18;8(8):1253. doi: 10.3390/microorganisms8081253 (PMC7465820; doi:10.3390/microorganisms8081253)
Supplement: Supplementary file 1 [file microorganisms-08-01253-s001.pdf]

microorganisms

Article supplementary materials

Supplementary materials belonging to

Host–Pathogen Interactions between *Xanthomonas fragariae* and Its Host *Fragaria* × *ananassa* Investigated with a Dual RNA-Seq Analysis

Michael Gétaz, Joanna Puławska, Theo H.M. Smits and Joël F. Pothier

Supplemental figures:

Figure S1. Symptomatic strawberry leaves.

Supplemental tables:

Table S1. RNA quantity and quality for each leaf replicates.
Table S2. Differentially expressed genes of *Xanthomonas fragariae*.
Table S3. Differentially expressed genes of *Fragaria* × *ananassa*.

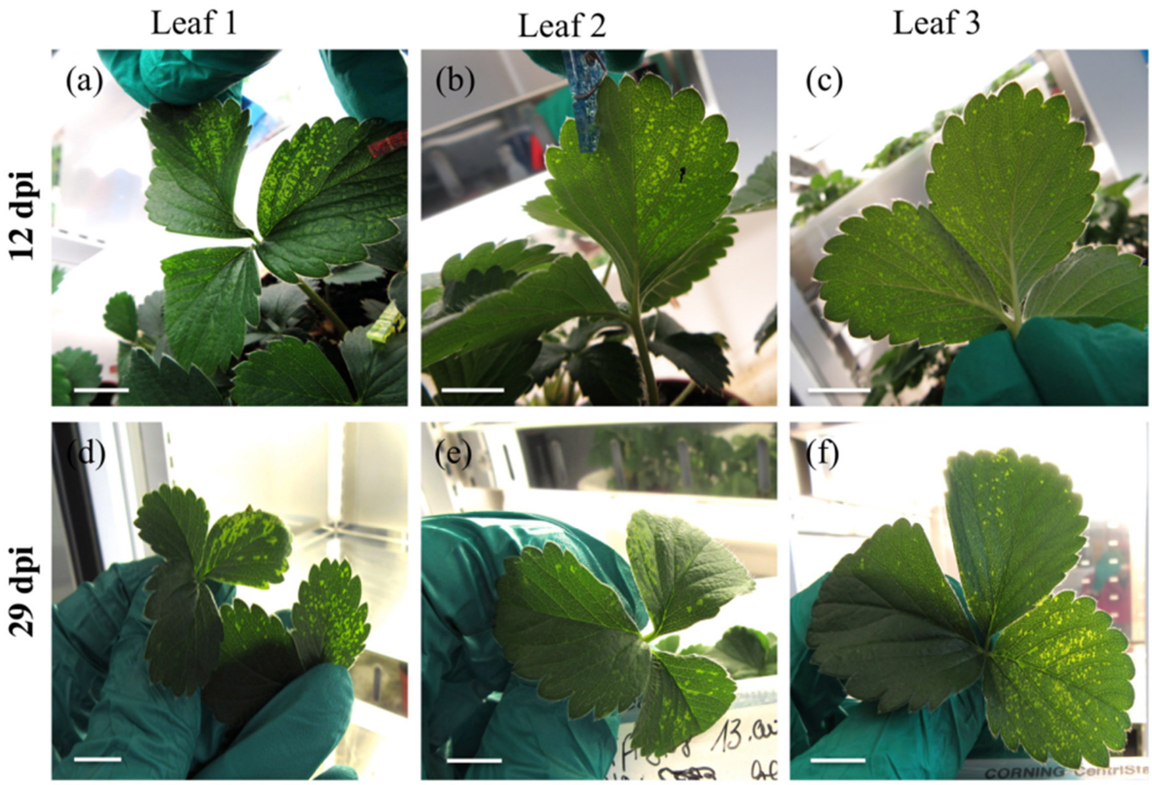

Figure S1. Symptomatic strawberry leaves. The pictures show representative results from spraying inoculation with *Xanthomonas fragariae* PD 885^{T} and collected (**a**–**c**) at 12 days post inoculation (dpi) and (**d**–**f**) at 29 dpi. Three replicates with symptoms were collected per dpi. The white line represents 1 cm on each picture.

Table S1. RNA quantity and quality for each leaf replicates. RNA samples of 12 days post inoculation (dpi) and 29 dpi were used for RNA sequencing. RNA samples were tested before DNase treatment for 260/280, 260/230 ratios and concentrations were reported and subsequently controlled for RNA integrity (RIN) after DNase treatment. Samples with best quality were selected for RNA-sequencing (bold).

Collection day	Leaf	Replicate	Micro-volume spectrophotometer (pre DNase)			Fragment Analyzer (post DNase)	
			260/280	260/230	Conc. (ng μl^{-1})	RIN	Conc. (ng μl^{-1})
12 dpi	1	1	1.79	1.26	82.94	7.8	824.71
		2	1.62	1.07	108.45	6.9	230.95
		**3**	**1.62**	**1.05**	**97.70**	**8.3**	**704.50**
	2	1	1.74	1.14	84.97	6.7	605.00
		2	1.64	1.01	62.79	6.6	367.18
		**3**	**1.75**	**1.12**	**147.60**	**6.8**	**139.98**
	3	1	1.88	1.35	113.37	7	442.10
		**2**	**1.85**	**1.37**	**78.84**	**7.4**	**86.82**
		3	1.81	1.34	113.97	6.6	728.31
29 dpi	1	**1**	**1.65**	**1.54**	**115.43**	**6.6**	**82.04**
		2	1.78	1.46	529.20	6.3	71.12
		3	1.77	1.59	176.51	NA [1]	NA
	2	1	1.78	1.21	62.25	6.7	50.69
		2	1.85	1.44	124.17	6.5	102.86
		**3**	**1.84**	**1.48**	**140.84**	**6.7**	**79.91**
	3	**1**	**1.67**	**1.33**	**145.14**	**7.4**	**166.6**
		2	1.77	1.31	130.99	8.9	6.06
		3	1.70	1.51	172.89	NA	NA

[1] NA: not available.

Table S2. Differentially expressed genes of *Xanthomonas fragariae*. This supplementary table provides the complete list of differentially expressed genes of *X. fragariae* while interacting with its host *Fragaria* × *ananassa* at 12- and 29-days post inoculation (dpi). Genomic information such as locus tag, locus, annotation as well as the gene ontology (GO) are listed below. Normalized values with Fragments Per Kilobase Million (FPKM) are listed for both collection days: 12 and 29 dpi.

Locus tag	Locus	Genome location	FPKM 12 dpi	FPKM 29 dpi	$\log_2$ (fold change)	Test stat	*P*-value	*q*-value	Annotation	Protein name	GO IDs [1]	GO names [1]	Enzyme codes	Enzyme names
PD885_RS11070	NZ_LT853882.1: 2378301-2380338	Chromosome	3,979	18,736	2.235	2.642	1.750E−03	0.037	hypothetical protein					
PD885_RS03890	NZ_LT853882.1: 843202-845575	Chromosome	4,415	18,303	2.051	2.643	1.300E−03	0.031	methyltransferase domain-containing protein		P:GO:0006464; F:GO:0016301; F:GO:0043167	P:cellular protein modification process; F:kinase activity; F:ion binding		
PD885_RS16725	NZ_LT853882.1: 3595055-3603270	Chromosome	5,607	21,383	1.931	3.003	5.000E−05	0.003	calcium-binding protein		C:GO:0005576; P:GO:0008150; F:GO:0043167	C:extracellular region; P:biological_process; F:ion binding		
PD885_RS04830	NZ_LT853882.1: 1055167-1058698	Chromosome	4,175	14,929	1.838	2.571	1.300E−03	0.031	histidine kinase		F:GO:0004871; C:GO:0005622; P:GO:0006464; F:GO:0016301	F:signal transducer activity; C:intracellular; P:cellular protein modification process; F:kinase activity	EC:2.7.13.3	Histidine kinase
PD885_RS14375	NZ_LT853882.1: 3099908-3101876	Chromosome	7,681	23,206	1.595	2.230	2.450E−03	0.050	DNA helicase		F:GO:0004386; F:GO:0004518; P:GO:0006259; P:GO:0009058; F:GO:0016887	F:helicase activity; F:nuclease activity; P:DNA metabolic process; P:biosynthetic process; F:ATPase activity	EC:3.1.30; EC:3.6.1.3; EC:3.1.21; EC:3.6.1.15	Acting on ester bonds; Adenosinetriphosphatase; Acting on ester bonds; Nucleoside-triphosphate phosphatase
PD885_RS02005	NZ_LT853882.1: 442628-444269	Chromosome	108,218	38,258	−1.500	−2.730	3.500E−04	0.014	molecular chaperonin GroEL	GroEL	C:GO:0005829; P:GO:0006457; C:GO:0032991; F:GO:0043167; F:GO:0051082	C:cytosol; P:protein folding; C:protein-containing complex; F:ion binding; F:unfolded protein binding		
PD885_RS14670	NZ_LT853882.1: 3143778-3147993	Chromosome	40,495	14,245	−1.507	−2.507	5.500E−04	0.019	DNA-directed RNA polymerase subunit beta'	RpoC	F:GO:0003677; P:GO:0009058; F:GO:0016779; P:GO:0034641	F:DNA binding; P:biosynthetic process; F:nucleotidyltransferase activity; P:cellular nitrogen compound metabolic process	EC:2.7.7.6	DNA-directed RNA polymerase
PD885_RS15605	NZ_LT853882.1: 3340297-3341782	Chromosome	64,043	22,461	−1.512	−2.395	1.700E−03	0.037	leucine rich protein					
PD885_RS14655	NZ_LT853882.1: 3140446-3142564	Chromosome	42,201	14,737	−1.518	−2.393	1.550E−03	0.034	elongation factor G	FusA	F:GO:0003924; C:GO:0005737; F:GO:0008135; F:GO:0043167	F:GTPase activity; C:cytoplasm; F:translation factor activity, RNA binding; F:ion binding	EC:3.6.1.15	Nucleoside-triphosphate phosphatase
PD885_RS17015	NZ_LT853882.1: 3659003-3661187	Chromosome	34,723	12,030	−1.529	−2.381	1.400E−03	0.032	tail-specific protease		F:GO:0008233	F:peptidase activity		
PD885_RS00915	NZ_LT853882.1: 215236-216646	Chromosome	53,692	17,737	−1.598	−2.445	8.500E−04	0.024	type I glutamate -ammonia ligase - glutamine synthetase	GlnA	C:GO:0005737; P:GO:0006520;	C:cytoplasm; P:cellular amino acid metabolic	EC:6.3.1.2	Glutamine synthetase

											P:GO:0009058; F:GO:0016874; F:GO:0043167; P:GO:0071941	process; P:biosynthetic process; F:ligase activity; F:ion binding; P:nitrogen cycle metabolic process		
PD885_RS14650	NZ_LT853882.1: 3139207-3140398	Chromosome	64,641	21,231	−1.606	−2.422	8.500E−04	0.024	elongation factor Tu	Tuf	F:GO:0003924; C:GO:0005737; F:GO:0008135; F:GO:0043167	F:GTPase activity; C:cytoplasm; F:translation factor activity, RNA binding; F:ion binding	EC:3.6.1.15	Nucleoside-triphosphate phosphatase
PD885_RS16510	NZ_LT853882.1: 3540199-3541201	Chromosome	56,108	18,226	−1.622	−2.265	2.200E−03	0.045	ketol-acid reductoisomerase		P:GO:0006520; P:GO:0009058; F:GO:0016491; F:GO:0016853; F:GO:0043167	P:cellular amino acid metabolic process; P:biosynthetic process; F:oxidoreductase activity; F:isomerase activity; F:ion binding	EC:1.1.1.86	Ketol-acid reductoisomerase (NADP(+))
PD885_RS10450	NZ_LT853882.1: 2231241-2232738	Chromosome	59,680	19,062	−1.647	−2.591	1.100E−03	0.028	type VI secretion system contractile sheath large subunit EvpB family	EvpB				
PD885_RS15285	NZ_LT853882.1: 3262650-3263610	Chromosome	65,631	20,705	−1.664	−2.380	1.800E−03	0.037	sulfotransferase domain-containing protein		F:GO:0003674	F:molecular function		
PD885_RS16620	NZ_LT853882.1: 3568032-3569205	Chromosome	49,356	15,470	−1.674	−2.381	1.650E−03	0.036	phosphate porin					
PD885_RS00675	NZ_LT853882.1: 157574-158369	Chromosome	100,598	31,406	−1.680	−2.449	1.650E−03	0.036	hypothetical protein					
PD885_RS15365	NZ_LT853882.1: 3277289-3277829	Chromosome	153,805	47,671	−1.690	−2.448	9.500E−04	0.025	hypothetical protein					
PD885_RS14720	NZ_LT853882.1: 3156611-3157802	Chromosome	73,146	22,539	−1.698	−2.627	4.500E−04	0.016	elongation factor Tu	Tuf	F:GO:0003924; C:GO:0005737; F:GO:0008135; F:GO:0043167	F:GTPase activity; C:cytoplasm; F:translation factor activity, RNA binding; F:ion binding	EC:3.6.1.15	Nucleoside-triphosphate phosphatase
PD885_RS00270	NZ_LT853882.1: 56761-58993	Chromosome	38,396	11,741	−1.709	−2.629	6.000E−04	0.020	NADP-dependent isocitrate dehydrogenase --Isocitrate dehydrogenase (NADP) (Oxalosuccinate decarboxylase) (IDH)		P:GO:0006091; F:GO:0016491; F:GO:0043167; P:GO:0044281	P:generation of precursor metabolites and energy; F:oxidoreductase activity; F:ion binding; P:small molecule metabolic process	EC:1.1.1.42	Isocitrate dehydrogenase (NADP(+))
PD885_RS00280	NZ_LT853882.1: 60991-61552	Chromosome	138,045	42,125	−1.712	−2.443	1.150E−03	0.028	hypothetical protein					
PD885_RS04345	NZ_LT853882.1: 944106-946857	Chromosome	37,416	11,375	−1.718	−2.574	9.000E−04	0.025	type VI secretion system tip protein VgrG	VgrG				
PD885_RS01900	NZ_LT853882.1: 411857-413252	Chromosome	37,890	11,495	−1.721	−2.415	9.000E−04	0.025	glycoside hydrolase family 10 protein		F:GO:0003674	F:molecular function		
PD885_RS07670	NZ_LT853882.1: 1645464-1646745	Chromosome	51,787	15,650	−1.726	−2.524	1.100E−03	0.028	hypothetical protein					
PD885_RS02795	NZ_LT853882.1: 621832-624714	Chromosome	46,366	13,922	−1.736	−2.400	1.300E−03	0.031	adenylyl-sulfate kinase		F:GO:0003924; P:GO:0006790; P:GO:0009058;	F:GTPase activity; P:sulfur compound metabolic process;	EC:2.7.7.4; EC:3.6.1.15	Sulfate adenylyltransferase; Nucleoside-

											F:GO:0016301; F:GO:0016779; P:GO:0019748; F:GO:0043167	P:biosynthetic process; F:kinase activity; F:nucleotidyltransferase activity; P:secondary metabolic process; F:ion binding	; EC:2.7.1.25	triphosphate phosphatase; Adenylyl-sulfate kinase
PD885_RS11875	NZ_LT853882.1: 2563240-2564200	Chromosome	48,989	14,577	−1.749	−2.308	1.800E−03	0.037	sulfatase modifying factor 1					
PD885_RS13005	NZ_LT853882.1: 2801740-2802466	Chromosome	80,018	23,808	−1.749	−2.396	1.750E−03	0.037	OmpA family protein -- cell envelope biogenesis protein		C:GO:0030312	C:external encapsulating structure		
PD885_RS19765	NZ_LT853882.1: 4257875-4259624	Chromosome	32,429	9,599	−1.756	−2.512	9.000E−04	0.025	sensor domain-containing diguanylate cyclase - diguanylate cyclase		F:GO:0004871; C:GO:0005622; P:GO:0006464; F:GO:0016301	F:signal transducer activity; C:intracellular; P:cellular protein modification process; F:kinase activity	EC:2.7.13.3	Histidine kinase
PD885_RS03685	NZ_LT853882.1: 805566-806532	Chromosome	63,900	18,885	−1.759	−2.482	8.500E−04	0.024	right-handed parallel beta-helix repeat-containing protein					
PD885_RS18320	NZ_LT853882.1: 3955123-3956248	Chromosome	53,203	15,384	−1.790	−2.470	5.500E−04	0.019	acyl-CoA desaturase		C:GO:0005575; P:GO:0006629; F:GO:0016491	C:cellular_component; P:lipid metabolic process; F:oxidoreductase activity	EC:1.14.19.3; EC:1.14.19	Acyl-CoA 6-desaturase; Acting on paired donors, with incorporation or reduction of molecular oxygen. The oxygen incorporated need not be derived from O(2)
PD885_RS15075	NZ_LT853882.1: 3219172-3222999	Chromosome	41,026	11,712	−1.809	−2.521	1.300E−03	0.031	LPS-assembly protein LptD - organic solvent tolerance protein	LptD	P:GO:0006810; P:GO:0022607; C:GO:0030312; P:GO:0061024	P:transport; P:cellular component assembly; C:external encapsulating structure; P:membrane organization		
PD885_RS15910	NZ_LT853882.1: 3411490-3412660	Chromosome	47,898	13,649	−1.811	−2.538	7.500E−04	0.023	ADP-forming succinate - CoA ligase subunit beta		P:GO:0006091; F:GO:0016874; F:GO:0043167; P:GO:0044281	P:generation of precursor metabolites and energy; F:ligase activity; F:ion binding; P:small molecule metabolic process	EC:6.2.1.5	Succinate--CoA ligase (ADP-forming)
PD885_RS14555	NZ_LT853882.1: 3129676-3130403	Chromosome	152,135	43,096	−1.820	−2.545	9.500E−04	0.025	30S ribosomal protein S5		F:GO:0003735; C:GO:0005829; C:GO:0005840; P:GO:0006412; F:GO:0019843	F:structural constituent of ribosome; C:cytosol; C:ribosome; P:translation; F:rRNA binding		
PD885_RS12560	NZ_LT853882.1: 2707408-2708254	Chromosome	61,770	17,417	−1.826	−2.419	1.450E−03	0.033	secreted protein					
PD885_RS06945	NZ_LT853882.1: 1503346-1504843	Chromosome	43,699	12,320	−1.827	−2.669	4.000E−04	0.015	lysine 6-aminotransferase		F:GO:0043167	F:ion binding		
PD885_RS16700	NZ_LT853882.1: 3587957-3590420	Chromosome	44,641	12,526	−1.833	−2.936	2.000E−04	0.008	TonB-dependent receptor		C:GO:0030312	C:external encapsulating structure		

PD885_RS17605	NZ_LT853882.1: 3793738-3794839	Chromosome	58,303	16,312	−1.838	−2.617	7.000E−04	0.022	sulfate ABC transporter substrate-binding protein		F:GO:0016887; F:GO:0022857	F:ATPase activity; F:transmembrane transporter activity	EC:3.6.1.3; EC:3.6.3.25; EC:3.6.1.15	Adenosinetriphosphatase; Sulfate-transporting ATPase; Nucleoside-triphosphate phosphatase
PD885_RS04770	NZ_LT853882.1: 1046922-1048353	Chromosome	58,735	16,412	−1.839	−2.768	4.500E−04	0.016	AraC family transcriptional regulator - DNA-binding protein	AraC	F:GO:0003677; F:GO:0003700; P:GO:0009058; P:GO:0034641	F:DNA binding; F:DNA binding transcription factor activity; P:biosynthetic process; P:cellular nitrogen compound metabolic process		
PD885_RS18325	NZ_LT853882.1: 3956257-3957334	Chromosome	46,761	12,880	−1.860	−2.508	1.400E−03	0.032	ferredoxin reductase - oxidoreductase		P:GO:0006091; F:GO:0016491; F:GO:0043167	P:generation of precursor metabolites and energy; F:oxidoreductase activity; F:ion binding		
PD885_RS17340	NZ_LT853882.1: 3731049-3732024	Chromosome	66,103	17,847	−1.889	−2.687	4.500E−04	0.016	type III secretion system effector protein	XopV				
PD885_RS05015	NZ_LT853882.1: 1101196-1101632	Chromosome	146,184	39,387	−1.892	−2.359	1.800E−03	0.037	glyoxalase		F:GO:0016491; F:GO:0016829	F:oxidoreductase activity; F:lyase activity		
PD885_RS16190	NZ_LT853882.1: 3471918-3473817	Chromosome	45,099	12,051	−1.904	−2.927	5.000E−05	0.003	type IV pilus secretin PilQ family protein - fimbrial assembly protein	PilQ	F:GO:0008565; C:GO:0030312	F:protein transporter activity; C:external encapsulating structure		
PD885_RS16225	NZ_LT853882.1: 3480442-3481732	Chromosome	51,354	13,640	−1.913	−2.748	3.500E−04	0.014	type II citrate synthase		C:GO:0005737; P:GO:0006091; F:GO:0016746; P:GO:0044281	C:cytoplasm; P:generation of precursor metabolites and energy; F:transferase activity, transferring acyl groups; P:small molecule metabolic process	EC:2.3.3.1	Citrate (Si)-synthase
PD885_RS10575	NZ_LT853882.1: 2269375-2269633	Chromosome	544,150	144,210	−1.916	−2.377	1.900E−03	0.039	stress-induced protein					
PD885_RS19485	NZ_LT853882.1: 4195809-4197219	Chromosome	48,774	12,781	−1.932	−2.824	2.000E−04	0.008	pyridine nucleotide-disulfide oxidoreductase		F:GO:0016491	F:oxidoreductase activity		
PD885_RS12550	NZ_LT853882.1: 2705902-2706391	Chromosome	140,365	36,438	−1.946	−2.552	7.000E−04	0.022	general stress protein		F:GO:0003674	F:molecular_function		
PD885_RS07850	NZ_LT853882.1: 1684140-1686255	Chromosome	58,102	14,967	−1.957	−3.061	5.000E−05	0.003	polyribonucleotide nucleotidyltransferase	Pnp	F:GO:0003723; C:GO:0005737; F:GO:0016779; P:GO:0034655; F:GO:0043167	F:RNA binding; C:cytoplasm; F:nucleotidyltransferase activity; P:nucleobase-containing compound catabolic process; F:ion binding	EC:2.7.7.8	Polyribonucleotide nucleotidyltransferase
PD885_RS03590	NZ_LT853882.1: 788222-788894	Chromosome	102,987	26,102	−1.980	−2.721	6.500E−04	0.021	OmpW family protein - membrane protein		C:GO:0005575	C:cellular_component		

PD885_RS12010	NZ_LT853882.1: 2599017-2599494	Chromosome	185,506	46,984	−1.981	−2.720	4.000E−04	0.015	hypothetical protein - Uncharacterised protein					
PD885_RS09535	NZ_LT853882.1: 2044105-2045791	Chromosome	171,639	42,751	−2.005	−3.002	1.000E−04	0.005	30S ribosomal protein S1		F:GO:0003723; F:GO:0003735; C:GO:0005840; P:GO:0006412	F:RNA binding; F:structural constituent of ribosome; C:ribosome; P:translation		
PD885_RS16470	NZ_LT853882.1 :3524801-3527693	Chromosome	73,304	18,126	−2.016	−3.147	1.000E−04	0.005	TonB-dependent receptor		C:GO:0030312	C:external encapsulating structure		
PD885_RS15035	NZ_LT853882.1: 3211734-3212736	Chromosome	69,980	17,229	−2.022	−2.844	1.500E−04	0.007	transcriptional regulator		F:GO:0003677; F:GO:0003700; P:GO:0009058; P:GO:0034641	F:DNA binding; F:DNA binding transcription factor activity; P:biosynthetic process; P:cellular nitrogen compound metabolic process		
PD885_RS14220	NZ_LT853882.1: 3064713-3068230	Chromosome	86,550	20,928	−2.048	−2.613	1.050E−03	0.027	multidrug transporter		C:GO:0005575; P:GO:0006810	C:cellular_component; P:transport		
PD885_RS14625	NZ_LT853882.1: 3136013-3136841	Chromosome	56,772	13,717	−2.049	−2.573	1.150E−03	0.028	50S ribosomal protein L2		F:GO:0003735; C:GO:0005840; P:GO:0006412; F:GO:0019843	F:structural constituent of ribosome; C:ribosome; P:translation; F:rRNA binding		
PD885_RS11680	NZ_LT853882.1: 2524008-2525064	Chromosome	69,904	16,789	−2.058	−2.919	2.000E−04	0.008	transposase					
PD885_RS07560	NZ_LT853882.1: 1626871-1627210	Chromosome	227,954	53,203	−2.099	−2.414	2.000E−03	0.041	lipoprotein					
PD885_RS13300	NZ_LT853882.1: 2865533-2866412	Chromosome	59,069	13,760	−2.102	−2.598	1.200E−03	0.029	elongation factor Ts	Tsf	C:GO:0005737; F:GO:0008135	C:cytoplasm; F:translation factor activity, RNA binding		
PD885_RS06375	NZ_LT853882.1: 1387780-1389565	Chromosome	60,307	13,859	−2.121	−3.364	5.000E−05	0.003	serine endopeptidase -- peptidase S8		F:GO:0008233	F:peptidase activity	EC:3.4.21	Acting on peptide bonds (peptidases)
PD885_RS15390	NZ_LT853882.1: 3281453-3283521	Chromosome	54,261	12,464	−2.122	−2.969	3.500E−04	0.014	serine protease		C:GO:0005575; F:GO:0008233	C:cellular_component; F:peptidase activity	EC:3.4.23	Acting on peptide bonds (peptidases)
PD885_RS01580	NZ_LT853882.1: 348309-348738	Chromosome	175,251	39,600	−2.146	−2.711	6.000E−04	0.020	50S ribosomal protein L13		F:GO:0003729; F:GO:0003735; C:GO:0005829; C:GO:0005840; P:GO:0006412	F:mRNA binding; F:structural constituent of ribosome; C:cytosol; C:ribosome; P:translation		
PD885_RS18845	NZ_LT853882.1: 4051958-4053686	Chromosome	94,908	21,263	−2.158	−3.653	5.000E−05	0.003	polyvinylalcohol dehydrogenase					
PD885_RS14145	NZ_LT853882.1: 3052859-3053633	Chromosome	94,789	21,177	−2.162	−2.990	3.000E−04	0.012	hypothetical protein		C:GO:0005575	C:cellular_component		

PD885_RS17545	NZ_LT853882.1: 3777461-3779594	Chromosome	60,433	13,229	−2.192	−3.529	5.000E−05	0.003	glycogen debranching enzyme GlgX	GlgX	P:GO:0005975; P:GO:0006091; P:GO:0009056; F:GO:0016798	P:carbohydrate metabolic process; P:generation of precursor metabolites and energy; P:catabolic process; F:hydrolase activity, acting on glycosyl bonds	EC:3.2.1.68	Isoamylase
PD885_RS19650	NZ_LT853882.1: 4233080-4234094	Chromosome	105,853	23,140	−2.194	−3.316	1.500E−04	0.007	hypothetical protein					
PD885_RS08815	NZ_LT853882.1: 1897776-1898475	Chromosome	131,256	27,932	−2.232	−3.238	5.000E−05	0.003	FKBP-type peptidyl-prolyl cis-trans isomerase		P:GO:0006457; P:GO:0006464; F:GO:0016853	P:protein folding; P:cellular protein modification process; F:isomerase activity	EC:5.2.1.8	Peptidylprolyl isomerase
PD885_RS06630	NZ_LT853882.1: 1440799-1441873	Chromosome	123,428	26,131	−2.240	−3.553	5.000E−05	0.003	EscU/YscU/HrcU family type III secretion system export apparatus switch protein	EscU/YscU/HrcU	C:GO:0005575; P:GO:0006810	C:cellular_component; P:transport		
PD885_RS04570	NZ_LT853882.1: 1003873-1005055	Chromosome	69,813	14,743	−2.243	−3.287	5.000E−05	0.003	polyketide cyclase		C:GO:0005575	C:cellular_component		
PD885_RS13680	NZ_LT853882.1: 2960339-2961794	Chromosome	67,213	14,193	−2.244	−3.377	5.000E−05	0.003	undecaprenyl-phosphate glucose phosphotransferase					
PD885_RS14520	NZ_LT853882.1: 3125069-3126068	Chromosome	68,594	14,472	−2.245	−3.098	2.000E−04	0.008	DNA-directed RNA polymerase subunit alpha		F:GO:0003677; C:GO:0005737; P:GO:0009058; F:GO:0016779; P:GO:0034641	F:DNA binding; C:cytoplasm; P:biosynthetic process; F:nucleotidyltransferase activity; P:cellular nitrogen compound metabolic process	EC:2.7.7.6	DNA-directed RNA polymerase
PD885_RS05755	NZ_LT853882.1: 1251583-1252876	Chromosome	95,123	19,956	−2.253	−3.578	5.000E−05	0.003	putative secreted protein		F:GO:0008233	F:peptidase activity	EC:3.4.24	Acting on peptide bonds (peptidases)
PD885_RS13280	NZ_LT853882.1: 2859762-2863142	Chromosome	86,800	17,959	−2.273	−2.726	9.000E−04	0.025	spore coat protein U		C:GO:0005575	C:cellular_component		
PD885_RS14700	NZ_LT853882.1: 3154771-3155200	Chromosome	142,748	29,466	−2.276	−2.652	1.550E−03	0.034	50S ribosomal protein L11	RplK	F:GO:0003735; C:GO:0005829; C:GO:0005840; P:GO:0006412; F:GO:0019843; P:GO:0022618; P:GO:0042254	F:structural constituent of ribosome; C:cytosol; C:ribosome; P:translation; F:rRNA binding; P:ribonucleoprotein complex assembly; P:ribosome biogenesis		
PD885_RS18515	NZ_LT853882.1: 3986216-3986563	Chromosome	375,260	74,875	−2.325	−3.004	2.000E−04	0.008	hypothetical protein					
PD885_RS16785	NZ_LT853882.1: 3615977-3616618	Chromosome	156,063	30,739	−2.344	−2.782	7.500E−04	0.023	hypothetical protein		C:GO:0005575	C:cellular_component		
PD885_RS12625	NZ_LT853882.1: 2720126-2720738	Chromosome	100,590	19,609	−2.359	−2.798	1.200E−03	0.029	superoxide dismutase		P:GO:0006950; F:GO:0016491; F:GO:0043167	P:response to stress; F:oxidoreductase activity; F:ion binding	EC:1.15.1.1; EC:1.11.1.7	Superoxide dismutase; Peroxidase

PD885_RS09050	NZ_LT853882.1: 1948127-1948694	Chromosome	97,254	18,608	−2.386	−2.816	9.000E−04	0.025	poly(hydroxyalcanoate) granule associated protein			
PD885_RS01740	NZ_LT853882.1: 376677-378864	Chromosome	73,967	14,121	−2.389	−3.908	5.000E−05	0.003	type III effector XopN	XopN		
PD885_RS18660	NZ_LT853882.1: 4008188-4009630	Chromosome	87,560	16,410	−2.416	−3.285	1.000E−04	0.005	secreted protein			
PD885_RS02425	NZ_LT853882.1: 534625-535318	Chromosome	147,785	27,411	−2.431	−3.530	5.000E−05	0.003	hypothetical protein		C:GO:0005575	C:cellular_component
PD885_RS11640	NZ_LT853882.1: 2516858-2518300	Chromosome	85,865	15,546	−2.466	−3.159	3.000E−04	0.012	secreted protein			
PD885_RS05180	NZ_LT853882.1: 1133923-1135210	Chromosome	97,515	17,569	−2.473	−3.757	5.000E−05	0.003	ATP-dependent Clp protease ATP-binding subunit ClpX	ClpX	P:GO:0006457; F:GO:0008233; F:GO:0043167; F:GO:0051082	P:protein folding; F:peptidase activity; F:ion binding; F:unfolded protein binding
PD885_RS05115	NZ_LT853882.1: 1123513-1124032	Chromosome	153,336	27,097	−2.501	−3.184	1.000E−04	0.005	peptidoglycan-associated lipoprotein Pal -- membrane protein	Pal	C:GO:0030312	C:external encapsulating structure
PD885_RS14580	NZ_LT853882.1: 3132482-3133025	Chromosome	162,400	28,410	−2.515	−3.330	1.500E−04	0.007	50S ribosomal protein L5		F:GO:0003735; C:GO:0005829; C:GO:0005840; P:GO:0006412; F:GO:0019843; P:GO:0022618; P:GO:0042254	F:structural constituent of ribosome; C:cytosol; C:ribosome; P:translation; F:rRNA binding; P:ribonucleoprotein complex assembly; P:ribosome biogenesis
PD885_RS06640	NZ_LT853882.1: 1442583-1442976	Chromosome	407,447	70,304	−2.535	−3.587	1.000E−04	0.005	type III secretion protein HrpB2	HrpB2	F:GO:0016301	F:kinase activity
PD885_RS00060	NZ_LT853882.1: 13509-13920	Chromosome	201,226	34,623	−2.539	−3.057	8.000E−04	0.023	biopolymer transporter ExbD	ExbD	C:GO:0005886; F:GO:0022857	C:plasma membrane; F:transmembrane transporter activity
PD885_RS12525	NZ_LT853882.1: 2700780-2701821	Chromosome	115,239	19,526	−2.561	−3.915	5.000E−05	0.003	RNA polymerase-binding protein DksA	DksA	C:GO:0005737; P:GO:0008150; F:GO:0043167	C:cytoplasm; P:biological_process; F:ion binding
PD885_RS15955	NZ_LT853882.1: 3421236-3421653	Chromosome	339,675	57,529	−2.562	−3.636	5.000E−05	0.003	pilin -- fimbrial protein		C:GO:0005623; P:GO:0007155	C:cell; P:cell adhesion
PD885_RS13230	NZ_LT853882.1: 2848533-2849583	Chromosome	100,645	16,942	−2.571	−3.767	5.000E−05	0.003	right-handed parallel beta-helix repeat-containing protein		F:GO:0016829	F:lyase activity
PD885_RS00140	NZ_LT853882.1: 32577-33009	Chromosome	119,184	19,844	−2.586	−2.671	1.800E−03	0.037	PepSY domain-containing protein			
PD885_RS06645	NZ_LT853882.1: 1442977-1443742	Chromosome	148,705	23,667	−2.651	−3.886	5.000E−05	0.003	EscJ/YscJ/HrcJ family type III secretion inner membrane ring protein	EscJ/YscJ/HrcJ	P:GO:0006810; C:GO:0030312	P:transport; C:external encapsulating structure
PD885_RS02570	NZ_LT853882.1: 572098-573079	Chromosome	80,981	12,759	−2.666	−3.528	5.000E−05	0.003	murein L,D-transpeptidase		F:GO:0003674	F:molecular_function
PD885_RS06075	NZ_LT853882.1: 1322335-1322761	Chromosome	218,909	34,404	−2.670	−3.214	2.000E−04	0.008	DNA-binding protein		F:GO:0003677	F:DNA binding

PD885_RS04035	NZ_LT853882.1: 876656-877115	Chromosome	124,204	19,260	−2.689	−2.875	6.500E−04	0.021	DNA-binding protein		F:GO:0003677; C:GO:0005622; P:GO:0009058; P:GO:0034641	F:DNA binding; C:intracellular; P:biosynthetic process; P:cellular nitrogen compound metabolic process		
PD885_RS08640	NZ_LT853882.1: 1860796-1862375	Chromosome	78,344	12,134	−2.691	−3.956	5.000E−05	0.003	UDP-glucose/GDP-mannose dehydrogenase family protein		P:GO:0005975; P:GO:0009058; F:GO:0016491	P:carbohydrate metabolic process; P:biosynthetic process; F:oxidoreductase activity	EC:1.1.1.22	UDP-glucose 6-dehydrogenase
PD885_RS16965	NZ_LT853882.1: 3649782-3650145	Chromosome	3706,540	573,939	−2.691	−4.305	5.000E−05	0.003	BON domain-containing protein -- membrane protein		C:GO:0005623; P:GO:0006950	C:cell; P:response to stress		
PD885_RS04680	NZ_LT853882.1: 1026045-1026366	Chromosome	307,164	47,152	−2.704	−2.974	1.400E−03	0.032	50S ribosomal protein L21	RplU	F:GO:0003735; C:GO:0005840; P:GO:0006412; F:GO:0019843	F:structural constituent of ribosome; C:ribosome; P:translation; F:rRNA binding		
PD885_RS05865	NZ_LT853882.1: 1286301-1286619	Chromosome	340,198	51,644	−2.720	−2.984	1.450E−03	0.033	ribosome-associated translation inhibitor RaiA	EaiA	F:GO:0003674; C:GO:0005829; C:GO:0005840; P:GO:0006412	F:molecular_function; C:cytosol; C:ribosome; P:translation		
PD885_RS05120	NZ_LT853882.1: 1124038-1124848	Chromosome	87,263	13,194	−2.726	−3.280	5.000E−05	0.003	tol-pal system protein YbgF	YgbF	P:GO:0000003; C:GO:0005623; P:GO:0007049; F:GO:0043167; P:GO:0051301; P:GO:0065003	P:reproduction; C:cell; P:cell cycle; F:ion binding; P:cell division; P:protein-containing complex assembly		
PD885_RS06675	NZ_LT853882.1: 1447891-1449712	Chromosome	75,527	11,418	−2.726	−4.188	5.000E−05	0.003	EscC/YscC/HrcC family type III secretion system outer membrane ring protein	EscC/ YscC/ HrcC	F:GO:0008565; C:GO:0030312	F:protein transporter activity; C:external encapsulating structure		
PD885_RS07280	NZ_LT853882.1: 1572279-1572735	Chromosome	3049,030	460,145	−2.728	−4.642	5.000E−05	0.003	DUF2383 domain-containing protein					
PD885_RS14030	NZ_LT853882.1: 3031888-3032374	Chromosome	571,756	85,634	−2.739	−4.330	5.000E−05	0.003	AAA ATPase containing von Willebrand factor type A (vWA) domain					
PD885_RS15745	NZ_LT853882.1: 3377914-3378472	Chromosome	96,366	14,293	−2.753	−2.969	1.450E−03	0.033	DNA starvation/stationary phase protection protein -- ferritin		F:GO:0003677; C:GO:0005623; P:GO:0006950; F:GO:0016491; P:GO:0042592; F:GO:0043167	F:DNA binding; C:cell; P:response to stress; F:oxidoreductase activity; P:homeostatic process; F:ion binding		
PD885_RS01600	NZ_LT853882.1: 349894-350512	Chromosome	93,501	13,837	−2.756	−2.984	1.550E−03	0.034	RNA pyrophosphohydrolase		F:GO:0003674	F:molecular_function		
PD885_RS06635	NZ_LT853882.1: 1442090-1442546	Chromosome	572,926	81,970	−2.805	−4.493	5.000E−05	0.003	HrpB1 family type III secretion system apparatus protein -- serine kinase	HrpB1	F:GO:0016301	F:kinase activity		
PD885_RS00055	NZ_LT853882.1: 13083-13506	Chromosome	167,181	23,836	−2.810	−3.083	9.000E−04	0.025	biopolymer transporter ExbD	ExbD	C:GO:0005886; F:GO:0022857	C:plasma membrane; F:transmembrane transporter activity		
PD885_RS03860	NZ_LT853882.1: 836518-836737	Chromosome	4516,120	628,499	−2.845	−4.088	5.000E−05	0.003	DUF465 domain-containing protein					

PD885_RS16985	NZ_LT853882.1: 3654035-3654416	Chromosome	490,052	66,910	−2.873	−4.062	5.000E−05	0.003	glycine zipper 2TM domain-containing protein -- membrane protein		C:GO:0005575	C:cellular_component		
PD885_RS15205	NZ_LT853882.1: 3245053-3245800	Chromosome	157,098	21,347	−2.880	−4.093	5.000E−05	0.003	DNA-binding response regulator		F:GO:0003677; C:GO:0005622; P:GO:0007165; P:GO:0009058; P:GO:0034641	F:DNA binding; C:intracellular; P:signal transduction; P:biosynthetic process; P:cellular nitrogen compound metabolic process		
PD885_RS00615	NZ_LT853882.1: 137965-139042	Chromosome	110,394	14,660	−2.913	−4.185	5.000E−05	0.003	cellulase		P:GO:0005975; F:GO:0016798	P:carbohydrate metabolic process; F:hydrolase activity, acting on glycosyl bonds	EC:3.2.1.4	Cellulase
PD885_RS00555	NZ_LT853882.1: 119751-120516	Chromosome	90,030	11,893	−2.920	−3.489	1.000E−04	0.005	GMP synthase		P:GO:0006520; F:GO:0016874	P:cellular amino acid metabolic process; F:ligase activity	EC:6.3.5.2	GMP synthase (glutamine-hydrolyzing)
PD885_RS02780	NZ_LT853882.1: 617248-619680	Chromosome	108,513	14,085	−2.946	−3.472	5.000E−05	0.003	assimilatory sulfite reductase (NADPH) hemoprotein subunit	CysI	C:GO:0005622; P:GO:0006520; P:GO:0006790; P:GO:0009058; F:GO:0016491; P:GO:0019748; C:GO:0032991; F:GO:0043167	C:intracellular; P:cellular amino acid metabolic process; P:sulfur compound metabolic process; P:biosynthetic process; F:oxidoreductase activity; P:secondary metabolic process; C:protein-containing complex; F:ion binding	EC:1.8.1.2	Assimilatory sulfite reductase (NADPH)
PD885_RS18530	NZ_LT853882.1: 3989095-3989489	Chromosome	193,122	24,917	−2.954	−3.043	5.500E−04	0.019	hypothetical protein					
PD885_RS02910	NZ_LT853882.1: 653223-653931	Chromosome	246,485	31,565	−2.965	−4.581	5.000E−05	0.003	type III secretion protein	XopR				
PD885_RS06595	NZ_LT853882.1: 1434552-1436551	Chromosome	286,876	36,386	−2.979	−3.720	5.000E−05	0.003	serine kinase		C:GO:0005575; F:GO:0016301	C:cellular_component; F:kinase activity		
PD885_RS13285	NZ_LT853882.1: 2863190-2863535	Chromosome	473,137	59,277	−2.997	−3.842	5.000E−05	0.003	hypothetical protein					
PD885_RS14680	NZ_LT853882.1: 3152571-3152937	Chromosome	311,532	37,225	−3.065	−3.404	7.000E−04	0.022	50S ribosomal protein L7/L12		F:GO:0003735; C:GO:0005840; P:GO:0006412	F:structural constituent of ribosome; C:ribosome; P:translation		
PD885_RS16005	NZ_LT853882.1: 3432940-3433177	Chromosome	1270,230	149,876	−3.083	−3.716	1.500E−04	0.007	hypothetical protein					
PD885_RS18445	NZ_LT853882.1: 3974541-3974904	Chromosome	585,027	68,297	−3.099	−4.330	5.000E−05	0.003	hypothetical protein					
PD885_RS02985	NZ_LT853882.1: 665214-665646	Chromosome	398,619	45,159	−3.142	−4.236	5.000E−05	0.003	putative transmembrane protein		C:GO:0005575	C:cellular_component		
PD885_RS02785	NZ_LT853882.1: 619803-621651	Chromosome	118,963	13,136	−3.179	−4.376	5.000E−05	0.003	assimilatory sulfite reductase (NADPH)flavoprotein subunit		P:GO:0006520; P:GO:0006790;	P:cellular amino acid metabolic process;	EC:1.8.1.2	Assimilatory sulfite reductase (NADPH)

											P:GO:0009058; F:GO:0016491; P:GO:0019748; F:GO:0043167	P:sulfur compound metabolic process; P:biosynthetic process; F:oxidoreductase activity; P:secondary metabolic process		
PD885_RS17775	NZ_LT853882.1: 3832365-3832962	Chromosome	862,357	90,880	−3.246	−4.588	5.000E−05	0.003	Ax21 family protein					
PD885_RS03240	NZ_LT853882.1: 717721-722299	Chromosome	126,586	13,259	−3.255	−5.036	5.000E−05	0.003	ice nucleation protein		F:GO:0003674; C:GO:0030312	F:molecular function; C:external encapsulating structure		
PD885_RS02460	NZ_LT853882.1: 544314-544767	Chromosome	355,917	36,849	−3.272	−4.393	5.000E−05	0.003	fasciclin domain-containing protein					
PD885_RS10885	NZ_LT853882.1: 2338459-2339659	Chromosome	138,792	13,720	−3.339	−4.681	5.000E−05	0.003	flagellin		F:GO:0005198; C:GO:0005576; C:GO:0005623; C:GO:0043226; P:GO:0048870	F:structural molecule activity; C:extracellular region; C:cell; C:organelle; P:cell motility		
PD885_RS17630	NZ_LT853882.1: 3798092-3799190	Chromosome	695,492	67,444	−3.366	−5.290	5.000E−05	0.003	outer membrane protein		C:GO:0030312; F:GO:0043167	C:external encapsulating structure; F:ion binding		
PD885_RS20125	NZ_LT853884.1: 13991-14420	plasmid pPD885-27	1017,380	97,907	−3.377	−4.688	5.000E−05	0.003	DUF3757 domain-containing protein - transmembrane protein		C:GO:0005575	C:cellular_component		
PD885_RS06310	NZ_LT853882.1: 1375434-1376253	Chromosome	277,423	24,950	−3.475	−5.073	5.000E−05	0.003	peptidase C1		F:GO:0008233	F:peptidase activity		
PD885_RS01365	NZ_LT853882.1: 308256-308616	Chromosome	654,233	58,365	−3.487	−4.569	5.000E−05	0.003	EF hand domain-containing protein		F:GO:0043167	F:ion binding		
PD885_RS14575	NZ_LT853882.1: 3132158-3132464	Chromosome	541,340	46,793	−3.532	−3.833	1.500E−04	0.007	30S ribosomal protein S14		F:GO:0003735; C:GO:0005840; P:GO:0006412; F:GO:0019843	F:structural constituent of ribosome; C:ribosome; P:translation; F:rRNA binding		
PD885_RS08135	NZ_LT853882.1: 1760116-1762894	Chromosome	556,275	47,981	−3.535	−6.762	5.000E−05	0.003	serine kinase		F:GO:0016301; P:GO:0044403	F:kinase activity; P:symbiont process		
PD885_RS10445	NZ_LT853882.1: 2230609-2231107	Chromosome	1439,010	116,119	−3.631	−5.151	5.000E−05	0.003	type VI secretion system tube protein Hcp	Hcp				
PD885_RS05980	NZ_LT853882.1: 1305616-1305949	Chromosome	8932,290	661,674	−3.755	−6.617	5.000E−05	0.003	helix-hairpin-helix domain-containing protein - competence protein ComEA	ComEA	F:GO:0016853	F:isomerase activity	EC:5.99.1.2	DNA topoisomerase
PD885_RS05550	NZ_LT853882.1: 1208019-1208259	Chromosome	1247,780	88,067	−3.825	−3.811	6.000E−04	0.020	acyl carrier protein		C:GO:0005829; P:GO:0005975; P:GO:0006629; P:GO:0009058; F:GO:0043167; P:GO:0044281	C:cytosol; P:carbohydrate metabolic process; P:lipid metabolic process; P:biosynthetic process; F:ion binding; P:small molecule metabolic process		
PD885_RS06575	NZ_LT853882.1: 1432121-1433111	Chromosome	549,827	38,482	−3.837	−5.410	5.000E−05	0.003	DNA-binding protein		C:GO:0005576; F:GO:0016829	C:extracellular region; F:lyase activity	EC:4.2.2.2	Pectate lyase

PD885_RS07565	NZ_LT853882.1: 1627431-1627773	Chromosome	1605,000	110,402	−3.862	−5.199	5.000E−05	0.003	membrane protein			
PD885_RS12670	NZ_LT853882.1: 2729743-2729968	Chromosome	2608,590	172,051	−3.922	−4.767	5.000E−05	0.003	cold-shock protein		F:GO:0003677; C:GO:0005737; P:GO:0009058; P:GO:0034641	F:DNA binding; C:cytoplasm; P:biosynthetic process; P:cellular nitrogen compound metabolic process
PD885_RS18450	NZ_LT853882.1: 3974907-3975770	Chromosome	224,034	14,338	−3.966	−3.611	1.350E−03	0.032	membrane protein		C:GO:0005575	C:cellular component
PD885_RS06580	NZ_LT853882.1: 1433397-1433868	Chromosome	918,734	57,796	−3.991	−5.264	5.000E−05	0.003	HpaB protein	HpaB	C:GO:0005737; P:GO:0006810; F:GO:0016491	C:cytoplasm; P:transport; F:oxidoreductase activity
PD885_RS06585	NZ_LT853882.1: 1433938-1434211	Chromosome	27186,500	1688,410	−4.009	−7.662	5.000E−05	0.003	serine kinase			
PD885_RS13075	NZ_LT853882.1: 2814123-2814339	Chromosome	2108,420	120,677	−4.127	−4.186	1.500E−04	0.007	cold-shock protein		F:GO:0003677; C:GO:0005737; P:GO:0009058; P:GO:0034641	F:DNA binding; C:cytoplasm; P:biosynthetic process; P:cellular nitrogen compound metabolic process
PD885_RS06590	NZ_LT853882.1: 1434287-1434530	Chromosome	1527,290	64,837	−4.558	−4.365	5.000E−05	0.003	serine kinase		F:GO:0016301; P:GO:0016310	F:kinase activity; P:phosphorylation
PD885_RS06680	NZ_LT853882.1: 1449789-1450173	Chromosome	18593,300	590,361	−4.977	−9.216	5.000E−05	0.003	Hpa1 protein	Hpa1		

[1] In GO IDs and GO names columns, F: molecular function, C: cellular component and P: biological process.

Table S3. Differentially expressed genes of *Fragaria* × *ananassa.* This supplementary table provides the complete list of differentially expressed genes of *Fragaria* × *ananassa* cv. Elsanta while challenged by the bacterium *Xanthomonas fragariae* at 12- and 29-days post inoculation (dpi). Genomic information such as locus tag, locus, annotation as well as the gene ontology (GO) are listed below. Normalised values with Fragments Per Kilobase Million (FPKM) are listed for both collection days: 12 and 29 dpi.

Locus tag	Locus	FPKM 12 dpi	FPKM 29 dpi	Log_2 (fold change)	Test stat	*P* value	*q* value	Protein function prediction	GO IDs [1]	GO names [1]
FvH4_6g16950	Fvb6: 10815316-10816828	9,568	435,901	5.510	7.753	5.000E−05	2.218E−03	thaumatin-like		
FvH4_3g38970	Fvb3: 33214319-33218539	1,167	49,528	5.407	5.698	2.500E−04	7.689E−03	asparagine synthase	P:GO:0006529; F:GO:0004066	P:asparagine biosynthetic process; F:asparagine synthase (glutamine-hydrolyzing) activity
FvH4_4g30150	Fvb4: 29928212-29930748	1,261	42,490	5.074	5.128	1.750E−03	3.000E−02	beta-1,3-glucanase	P:GO:0005975; F:GO:0004553	P:carbohydrate metabolic process; F:hydrolase activity, hydrolyzing O-glycosyl compounds
FvH4_4g10610	Fvb4: 14349186-14350693	2,044	54,450	4.735	4.848	1.450E−03	2.666E−02	chitinase 4-like	P:GO:0005975; P:GO:0016998; P:GO:0006032; F:GO:0004568	P:carbohydrate metabolic process; P:cell wall macromolecule catabolic process; P:chitin catabolic process; F:chitinase activity
FvH4_3g28370	Fvb3: 21335348-21337404	5,691	135,042	4.569	7.102	5.000E−05	2.218E−03	glucan endo-1,3-beta-glucosidase-like	P:GO:0005975; F:GO:0004553	P:carbohydrate metabolic process; F:hydrolase activity, hydrolyzing O-glycosyl compounds
FvH4_5g01820	Fvb5: 1151603-1152293	14,326	248,482	4.116	6.165	5.000E−05	2.218E−03	thaumatin, protein P21-like		
FvH4_5g06210	Fvb5: 3658609-3660218	5,423	73,463	3.760	5.465	5.000E−05	2.218E−03	glucan endo-1,3-beta-glucosidase, basic isoform-like	P:GO:0005975; F:GO:0004553	P:carbohydrate metabolic process; F:hydrolase activity, hydrolyzing O-glycosyl compounds
FvH4_2g16350	Fvb2: 14268542-14272018	2,647	31,784	3.586	3.570	8.000E−04	1.868E−02	bromodomain-containing protein 4-like		
FvH4_7g07900	Fvb7: 7795506-7799128	5,412	64,637	3.578	6.225	5.000E−05	2.218E−03	protein NRT1/ PTR FAMILY 7.2-like	P:GO:0055085; C:GO:0016020; F:GO:0022857	P:transmembrane transport; C:membrane; F:transmembrane transporter activity
FvH4_4g11660	Fvb4: 15363633-15367751	1,461	15,406	3.399	4.334	5.000E−05	2.218E−03	extensin-2-like, partial	P:GO:0009664; F:GO:0005199	P:plant-type cell wall organization; F:structural constituent of cell wall
FvH4_6g41890	Fvb6: 32810646-32811799	11,248	111,142	3.305	4.773	5.000E−05	2.218E−03	uncharacterized protein		
FvH4_4g31070	Fvb4: 30387328-30388714	15,595	152,377	3.289	5.956	5.000E−05	2.218E−03	NAC transcription factor 29-like	P:GO:0006355; F:GO:0003677	P:regulation of transcription, DNA-templated; F:DNA binding
FvH4_1g13730	Fvb1: 7567770-7570682	1,473	11,710	2.991	3.121	3.600E−03	4.876E−02	vicilin-like antimicrobial peptides 2-2	F:GO:0045735	F:nutrient reservoir activity
FvH4_6g21670	Fvb6: 15226263-15226966	8,597	68,282	2.990	3.301	1.450E−03	2.666E−02	uncharacterized protein		
FvH4_5g36280	Fvb5: 26613578-26615638	3,095	24,342	2.976	3.160	3.250E−03	4.563E−02	uncharacterized protein		

FvH4_6g40280	Fvb6: 31792187-31793433	4,115	31,230	2.924	3.293	1.250E−03	2.433E−02	uncharacterized protein		
FvH4_2g20170	Fvb2: 16971536-16974605	1,111	7,941	2.838	3.158	1.800E−03	3.073E−02	multiple C2 and transmembrane domain-containing protein 1		
FvH4_2g02860	Fvb2: 2250275-2250770	15,815	110,683	2.807	3.586	1.500E−04	5.311E−03	pathogenesis-related protein 1A-like (Cysteine-rich)	C:GO:0005576	C:extracellular region
FvH4_6g24670	Fvb6: 18708864-18710041	6,430	43,565	2.760	3.731	1.500E−04	5.311E−03	thaumatin-like protein 1b		
FvH4_5g38040	Fvb5: 28094328-28096045	4,773	32,267	2.757	4.102	5.000E−05	2.218E−03	aminocyclopropane-1-carboxylate oxidase homolog	P:GO:0055114; F:GO:0016491	P:oxidation-reduction process; F:oxidoreductase activity
FvH4_5g04360	Fvb5: 2573220-2577327	11,428	76,964	2.752	2.812	1.650E−03	2.879E−02	probable WRKY transcription factor 53	P:GO:0006355; F:GO:0003700; F:GO:0043565	P:regulation of transcription, DNA-templated; F:DNA binding transcription factor activity; F:sequence-specific DNA binding
FvH4_4g24790	Fvb4: 26799592-26800879	5,278	35,394	2.746	3.761	1.000E−04	3.925E−03	vinorine synthase-like	F:GO:0016747	F:transferase activity, transferring acyl groups other than amino-acyl groups
FvH4_6g04320	Fvb6: 2340299-2346769	0,790	5,243	2.730	2.914	3.300E−03	4.606E−02	uncharacterized protein		
FvH4_1g00730	Fvb1: 376613-380159	1,466	9,713	2.729	3.024	2.300E−03	3.578E−02	uncharacterized protein	P:GO:0042138; P:GO:0000212	P:meiotic DNA double-strand break formation; P:meiotic spindle organization
FvH4_6g23550	Fvb6: 17607047-17612704	2,444	16,170	2.726	4.034	1.000E−04	3.925E−03	probable galactinol-sucrose galactosyltransferase 2	F:GO:0003824	F:catalytic activity
FvH4_5g38050	Fvb5: 28100620-28101688	18,925	122,111	2.690	5.256	5.000E−05	2.218E−03	leucoanthocyanidin dioxygenase-like	P:GO:0055114; F:GO:0016491	P:oxidation-reduction process; F:oxidoreductase activity
FvH4_6g27100	Fvb6: 20794675-20797557	1,393	8,841	2.666	3.021	2.850E−03	4.168E−02	seed biotin-containing protein SBP65 isoform X1		
FvH4_2g30510	Fvb2: 23619414-23622686	11,576	71,606	2.629	5.428	5.000E−05	2.218E−03	primary amine oxidase-like, Glycine, serine and threonine metabolism	P:GO:0009308; P:GO:0055114; FGO:0005507; F:GO:0008131; F:GO:0048038	P:amine metabolic process; P:oxidation-reduction process; F:copper ion binding; F:primary amine oxidase activity; F:quinone binding
FvH4_4g09530	Fvb4: 11220461-11225589	1,768	10,602	2.584	3.310	7.000E−04	1.719E−02	probable linoleate 9S-triterpen 5	P:GO:0055114; F:GO:0046872; F:GO:0016491; F:GO:0016702; F:GO:0005515	P:oxidation-reduction process; F:metal ion binding; F:oxidoreductase activity; F:oxidoreductase activity, acting on single donors with incorporation of molecular oxygen, incorporation of two atoms of oxygen; F:protein binding
FvH4_7g17320	Fvb7: 14744095-14745843	6,514	38,227	2.553	4.184	5.000E−05	2.218E−03	homeobox-leucine zipper protein ATHB-12-like	P:GO:0006355; F:GO:0003677; F:GO:0003700; F:GO:0043565	P:regulation of transcription, DNA-templated; F:DNA binding; F:DNA binding transcription factor activity; F:sequence-specific DNA binding
FvH4_4g29810	Fvb4: 29777129-29779171	1,816	10,606	2.546	2.799	3.250E−03	4.563E−02	cytochrome p450 78A5	P:GO:0055114; F:GO:0020037; F:GO:0005506; F:GO:0016705	P:oxidation-reduction process; F:heme binding; F:iron ion binding; F:oxidoreductase activity, acting on paired donors, with incorporation or reduction of molecular oxygen

FvH4_6g25180	Fvb6: 19099967-19102224	7,714	43,552	2.497	4.012	5.000E−05	2.218E−03	bromodomain-containing protein 4-like		
FvH4_1g10600	Fvb1: 5814344-5815342	7,910	43,798	2.469	2.835	3.550E−03	4.847E−02	endochitinase-like protein	P:GO:0005975; P:GO:0016998; P:GO:0006032; F:GO:0004568	P:carbohydrate metabolic process; P:cell wall macromolecule catabolic process; P:chitin catabolic process; F:chitinase activity
FvH4_4g13000	Fvb4: 16653443-16654859	3,050	16,690	2.452	2.851	1.900E−03	3.175E−02	crocetin glucosyltransferase, chloroplastic-like	P:GO:0008152; F:GO:0016758	P:metabolic process; F:transferase activity, transferring hexosyl groups
FvH4_4g17440	Fvb4: 21375829-21376138	53,013	288,274	2.443	3.340	4.000E−04	1.118E−02	monothiol glutaredoxin-S2-like	P:GO:0045454; F:GO:0009055; F:GO:0015035	P:cell redox homeostasis; F:electron transfer activity; F:protein disulfide oxidoreductase activity
FvH4_5g00420	Fvb5: 282931-283525	11,493	60,466	2.395	2.961	1.400E−03	2.610E−02	pectinesterase inhibitor domain	F:GO:0004857	F:enzyme inhibitor activity
FvH4_5g23420	Fvb5: 14763405-14766264	1,692	8,894	2.394	3.023	1.450E−03	2.666E−02	disease resistance protein RPM1-like (Leucine-rich repeat domain superfamily)	F:GO:0043531	F:ADP binding
FvH4_3g20030	Fvb3: 13176342-13177700	501,779	2622,480	2.386	3.024	4.000E−04	1.118E−02	metallothionein-like protein		
FvH4_6g39330	Fvb6: 31084771-31086238	3,713	19,344	2.381	3.014	9.500E−04	2.088E−02	putative UDP-glucose flavonoid 3-O-glucosyltransferase 3	P:GO:0008152; F:GO:0016758	P:metabolic process; F:transferase activity, transferring hexosyl groups
FvH4_4g24800	Fvb4: 26811836-26813180	11,648	59,679	2.357	4.358	5.000E−05	2.218E−03	vinorine synthase-like	F:GO:0016747	F:transferase activity, transferring acyl groups other than amino-acyl groups
FvH4_6g24680	Fvb6: 18714133-18715667	8,766	42,650	2.283	3.791	1.500E−04	5.311E−03	glucan endo-1,3-beta-glucosidase, basic isoform-like	P:GO:0005975; F:GO:0004553	P:carbohydrate metabolic process; F:hydrolase activity, hydrolyzing O-glycosyl compounds
FvH4_6g05480	Fvb6: 3115211-3117821	2,464	11,955	2.278	3.060	7.500E−04	1.786E−02	RNA-binding protein cabeza	P:GO:0030001; F:GO:0005488; F:GO:0046872	P:metal ion transport; F:binding; F:metal ion binding
FvH4_6g23160	Fvb6: 16997641-16998094	19,184	93,008	2.277	2.881	2.550E−03	3.857E−02	uncharacterized protein		
FvH4_6g47270	Fvb6: 35887415-35889469	2,305	11,079	2.265	2.688	3.400E−03	4.696E−02	BAG family molecular chaperone regulator 8, chloroplastic	F:GO:0051087	F:chaperone binding
FvH4_3g04670	Fvb3: 2683369-2685154	8,704	41,767	2.263	4.014	5.000E−05	2.218E−03	polyphenol oxidase, chloroplastic-like	F:GO:0008152; P:GO:0055114; P:GO:0046148; F:GO:0004097; F:GO:0016491	P:metabolic process; P:oxidation-reduction process; P:pigment biosynthetic process; F:catechol oxidase activity; F:oxidoreductase activity
FvH4_3g12400	Fvb3: 7371636-7378988	1,456	6,946	2.254	2.962	1.050E−03	2.206E−02	mitochondrial chaperone BCS1-like	F:GO:0005524	F:ATP binding
FvH4_1g16030	Fvb1: 9104411-9107209	6,277	29,886	2.251	3.889	5.000E−05	2.218E−03	heat shock factor protein HSF24	P:GO:0006355; C:GO:0005634; F:GO:0003700; F:GO:0043565	P:regulation of transcription, DNA-templated; C:nucleus; F:DNA binding transcription factor activity; F:sequence-specific DNA binding
FvH4_3g34960	Fvb3: 30174876-30176674	4,575	21,273	2.217	2.901	1.500E−03	2.716E−02	transcription factor MYB59	F:GO:0003677	F:DNA binding

FvH4_5g24920	Fvb5: 16382894-16383420	18,952	87,214	2.202	2.845	1.900E−03	3.175E−02	putative F-box/lrr-repeat protein 23		
FvH4_1g21540	Fvb1: 13515805-13518950	3,783	17,374	2.199	3.526	3.000E−04	8.972E−03	L-ascorbate oxidase	P:GO:0055114; C:GO:0005576; F:GO:0005507; F:GO:0005507; F:GO:0016491	P:oxidation-reduction process; C:extracellular region; F:copper ion binding; F:copper ion binding; F:oxidoreductase activity
FvH4_3g45520	Fvb3: 37735078-37737977	1,644	7,323	2.155	2.637	2.650E−03	3.973E−02	leucine-rich repeat receptor protein kinase EXS-like	F:GO:0005515	F:protein binding
FvH4_2g01360	Fvb2: 1278041-1280264	2,144	9,536	2.153	2.659	2.950E−03	4.257E−02	copper amine oxidase 1-like	P:GO:0009308; P:GO:0055114; F:GO:0005507; F:GO:0008131; F:GO:0048038	P:amine metabolic process; P:oxidation-reduction process; F:copper ion binding; F:primary amine oxidase activity; F:quinone binding
FvH4_3g02840	Fvb3: 1482707-1497385	2,177	9,671	2.152	3.293	2.500E−04	7.689E−03	cysteine-rich receptor-like protein kinase 10	P:GO:0006468; F:GO:0005524; F:GO:0005524; F:GO:0004672	P:protein phosphorylation; F:ATP binding; F:ATP binding; F:protein kinase activity
FvH4_3g13880	Fvb3: 8332999-8335390	9,754	41,993	2.106	4.278	5.000E−05	2.218E−03	calcium-transporting ATPase 12, plasma membrane-type-like	F:GO:0000166	F:nucleotide binding
FvH4_7g32800	Fvb7: 8335390-23507815	7,948	34,185	2.105	2.903	1.500E−03	2.716E−02	small heat shock protein, chloroplastic-like		
FvH4_2g17080	Fvb2: 14803071-14804862	11,688	49,961	2.096	4.129	5.000E−05	2.218E−03	3-hydroxy-3-methylglutaryl-coenzyme A reductase 1-like	P:GO:0015936; P:GO:0008299; P:GO:0055114; C:GO:0016021; F:GO:0050661; F:GO:0050662; F:GO:0050662; F:GO:0004420; F:GO:0016616;	P:coenzyme A metabolic process; P:isoprenoid biosynthetic process; P:oxidation-reduction process; C:integral component of membrane; F:NADP binding; F:coenzyme binding; F:coenzyme binding; F:hydroxymethylglutaryl-CoA reductase (NADPH) activity; F:oxidoreductase activity, acting on the CH-OH group of donors, NAD or NADP as acceptor;
FvH4_3g25960	Fvb3: 18790253-18792197	35,375	151,052	2.094	3.754	5.000E−05	2.218E−03	probable CCR4-associated factor 1 homolog 11 (ribonuclease H-like superfamily)	F:GO:0003676	F:nucleic acid binding
FvH4_4g36390	Fvb4: 33348595-33353361	2,725	11,631	2.093	2.898	1.000E−03	2.150E−02	cyclic nucleotide-gated ion channel 1-like (Plant-pathogen interaction)	P:GO:0006811; P:GO:0055085; C:GO:0016020; F:GO:0005216	P:ion transport; P:transmembrane transport; C:membrane; F:ion channel activity
FvH4_2g04750	Fvb2: 3685624-3688124	6,381	27,133	2.088	3.719	5.000E−05	2.218E−03	probable indole-3-acetic acid-amido synthetase GH3.1		
FvH4_6g21490	Fvb6: 15064482-15068435	5,736	24,351	2.086	3.794	5.000E−05	2.218E−03	F-box/kelch-repeat protein At1g15670-like	F:GO:0005515	F:protein binding
FvH4_4g37300	Fvb4: 33819432-33820850	8,130	34,079	2.068	3.321	1.000E−04	3.925E−03	uncharacterized protein	F:GO:0005515	F:protein binding
FvH4_2g02590	Fvb2: 2068496-2071894	7,197	29,973	2.058	3.760	5.000E−05	2.218E−03	squalene monooxygenase-like (sesquiterpenoid and triterpenoid biosynthesis)	P:GO:0055114; C:GO:0016021; F:GO:0050660;	P:oxidation-reduction process; C:integral component of membrane; F:flavin adenine dinucleotide binding; F:oxidoreductase activity; F:squalene monooxygenase activity

									F:GO:0016491; F:GO:0004506	
FvH4_6g39430	Fvb6: 31147472-31148909	4,596	19,060	2.052	2.775	1.700E−03	2.957E−02	putative UDP-glucose flavonoid 3-O-glucosyltransferase 3	P:GO:0008152; F:GO:0016758	P:metabolic process; F:transferase activity, transferring hexosyl groups
FvH4_5g05100	Fvb5: 2978458-2983365	3,479	14,260	2.035	3.594	5.000E−05	2.218E−03	probable alpha,alpha-trehalose-phosphate synthase	P:GO:0005992; F:GO:0003824	P:trehalose biosynthetic process; F:catalytic activity
FvH4_2g02540	Fvb2: 2024794-2028528	4,636	18,866	2.025	3.877	5.000E−05	2.218E−03	receptor-like protein kinase HAIKU2 (leucine-rich repeat)	P:GO:0006468; F:GO:0005524; F:GO:0005524; F:GO:0005515; F:GO:0004672	P:protein phosphorylation; F:ATP binding; F:ATP binding; F:protein binding; F:protein kinase activity
FvH4_5g07000	Fvb5: 4141516-4142707	6,741	27,102	2.007	2.916	9.000E−04	2.034E−02	CD2 antigen cytoplasmic tail-binding protein 2		
FvH4_3g41580	Fvb3: 34915786-34919646	2,295	9,184	2.000	3.076	5.000E−04	1.311E−02	uncharacterized protein	F:GO:0043531	F:ADP binding
FvH4_4g06830	Fvb4: 6132454-6133929	9,285	36,685	1.982	3.371	1.000E−04	3.925E−03	probable WRKY transcription factor 11	P:GO:0006355; F:GO:0003700; F:GO:0043565	P:regulation of transcription, DNA-templated; F:DNA binding transcription factor activity; F:sequence-specific DNA binding
FvH4_4g30080	Fvb4: 29893568-29895524	3,038	12,000	1.982	2.622	2.900E−03	4.205E−02	pentatricopeptide repeat-containing protein	F:GO:0005515	F:protein binding
FvH4_2g39800	Fvb2: 28484217-28488901	2,473	9,746	1.978	2.844	1.000E−03	2.150E−02	probable methyltransferase PMT28	F:GO:0008168	F:methyltransferase activity
FvH4_2g06030	Fvb2: 5029664-5035895	3,954	15,564	1.977	3.435	1.500E−04	5.311E−03	hypothetical protein	P:GO:0005975; F:GO:0004556; F:GO:0005509; F:GO:0003824	P:carbohydrate metabolic process; F:alpha-amylase activity; F:calcium ion binding; F:catalytic activity
FvH4_5g11800	Fvb5: 6673896-6678511	14,756	57,162	1.954	4.152	5.000E−05	2.218E−03	uncharacterized protein	P:GO:0006950; F:GO:0005516	P:response to stress; F:calmodulin binding
FvH4_2g39820	Fvb2: 28495138-28495537	33,707	130,483	1.953	2.776	2.150E−03	3.425E−02	parathymosin-like		
FvH4_6g45580	Fvb6: 34959190-34962068	6,204	23,833	1.942	3.197	2.000E−04	6.539E−03	probable:endo-1,3(4)-beta-glucanase ARB_01444	F:GO:0052861	F:glucan endo-1,3-beta-glucanase activity, C-3 substituted reducing group
FvH4_4g02020	Fvb4: 1803033-1805556	2,326	8,912	1.938	2.588	3.300E−03	4.606E−02	pentatricopeptide repeat-containing protein At3g23020	F:GO:0005515	F:protein binding
FvH4_7g24490	Fvb7: 18883244-18885614	2,378	9,111	1.938	2.529	2.900E−03	4.205E−02	putative serine/threonine-protein kinase-like protein CCR3	P:GO:0006468; F:GO:0005524; F:GO:0005524; F:GO:0004672	P:protein phosphorylation; F:ATP binding; F:ATP binding; F:protein kinase activity
FvH4_4g22140	Fvb4: 25081107-25082016	8,745	33,293	1.929	2.691	1.950E−03	3.236E−02	uncharacterized protein		
FvH4_7g30310	Fvb7: 22115708-22117069	116,714	440,275	1.915	4.259	5.000E−05	2.218E−03	FK506-binding protein 4-like	P:GO:0030001; F:GO:0046872	P:metal ion transport; F:metal ion binding

FvH4_5g32320	Fvb5: 23337902-23339656	6,722	25,343	1.915	3.080	4.500E−04	1.218E−02	transcription repressor MYB6	F:GO:0003677	F:DNA binding
FvH4_1g22860	Fvb1: 14804592-14806032	5,240	19,421	1.890	2.702	1.500E−03	2.716E−02	splicing factor 3A subunit 3-like isoform X1	P:GO:0000398; C:GO:0005634; C:GO:0005681; F:GO:0003723; F:GO:0003676; F:GO:0008270	P:mRNA splicing, via spliceosome; C:nucleus; C:spliceosomal complex; F:RNA binding; F:nucleic acid binding; F:zinc ion binding
FvH4_2g17090	Fvb2: 14812958-14814869	14,626	54,020	1.885	3.981	5.000E−05	2.218E−03	3-hydroxy-3-methylglutaryl-coenzyme A reductase 1-like	P:GO:0015936; P:GO:0055114; F:GO:0050662; F:GO:0004420; F:GO:0016616	P:coenzyme A metabolic process; P:oxidation-reduction process; F:coenzyme binding; F:hydroxymethylglutaryl-CoA reductase (NADPH) activity; F:oxidoreductase activity, acting on the CH-OH group of donors, NAD or NADP as acceptor
FvH4_6g10510	Fvb6: 6310957-6313581	111,484	408,547	1.874	3.216	1.000E−04	3.925E−03	probable WRKY transcription factor 33	P:GO:0006355; F:GO:0003700; F:GO:0043565	P:regulation of transcription, DNA-templated; F:DNA binding transcription factor activity; F:sequence-specific DNA binding
FvH4_7g14060	Fvb7: 12491034-12492810	12,859	47,102	1.873	3.801	5.000E−05	2.218E−03	probable leucine-rich repeat receptor-like protein kinase At1g35710	F:GO:0005515	F:protein binding
FvH4_1g29930	Fvb1: 23674191-23675538	15,214	55,629	1.870	3.535	5.000E−05	2.218E−03	U-box domain-containing protein 21	P:GO:0016567; F:GO:0005488; F:GO:0004842	P:protein ubiquitination; F:binding; F:ubiquitin-protein transferase activity
FvH4_3g38990	Fvb3: 33233291-33235593	11,458	41,810	1.868	3.514	5.000E−05	2.218E−03	probable protein phosphatase 2C 25	P:GO:0006470; F:GO:0003824; F:GO:0043169; F:GO:0004722	P:protein dephosphorylation; F:catalytic activity; F:cation binding; F:protein serine/threonine phosphatase activity
FvH4_4g34660	Fvb4: 32309876-32313718	7,277	26,259	1.851	3.422	5.000E−05	2.218E−03	aldehyde dehydrogenase family 2 member B7, mitochondrial-like	P:GO:0008152; P:GO:0055114; F:GO:0016491	P:metabolic process; P:oxidation-reduction process; F:oxidoreductase activity
FvH4_4g04000	Fvb4: 3463105-3467679	2,019	7,217	1.838	2.519	2.550E−03	3.857E−02	kinesin-like protein KIF22	P:GO:0007018; P:GO:0007018; F:GO:0005524; F:GO:0008017; F:GO:0003777	P:microtubule-based movement; P:microtubule-based movement; F:ATP binding; F:microtubule binding; F:microtubule motor activity
FvH4_2g35260	Fvb2: 26026906-26031080	3,632	12,943	1.833	3.379	5.000E−05	2.218E−03	receptor-like protein kinase 5	P:GO:0006468; F:GO:0005524; F:GO:0005524; F:GO:0005515; F:GO:0004672	P:protein phosphorylation; F:ATP binding; F:ATP binding; F:protein binding; F:protein kinase activity
FvH4_2g16180	Fvb2: 14147225-14149397	25,433	90,112	1.825	4.003	5.000E−05	2.218E−03	NAC transcription factor 29	P:GO:0006355; F:GO:0003677	P:regulation of transcription, DNA-templated; F:DNA binding
FvH4_4g09780	Fvb4: 11758877-11762248	4,077	14,314	1.812	3.165	2.000E−04	6.539E−03	probable alpha,alpha-trehalose-phosphate synthase [UDP-forming]	P:GO:0005992; F:GO:0003824	P:trehalose biosynthetic process; F:catalytic activity
FvH4_6g40950	Fvb6: 32360300-32364560	2,490	8,742	1.812	2.988	5.500E−04	1.421E−02	putative calcium-transporting ATPase 13, plasma membrane-type	C:GO:0016021; F:GO:0000166	C:integral component of membrane; F:nucleotide binding
FvH4_6g03790	Fvb6: 2051769-2055392	9,808	34,319	1.807	3.571	5.000E−05	2.218E−03	cysteine synthase-like isoform X1	P:GO:0006535; F:GO:0004124	P:cysteine biosynthetic process from serine; F:cysteine synthase activity

FvH4_1g24380	Fvb1: 16242627-16247167	5,572	19,384	1.799	3.408	5.000E−05	2.218E−03	laccase-15-like isoform X1	P:GO:0046274; P:GO:0055114; C:GO:0048046; F:GO:0005507; F:GO:0005507; F:GO:0052716; F:GO:0016491	P:lignin catabolic process; P:oxidation-reduction process; C:apoplast; F:copper ion binding; F:copper ion binding; F:hydroquinone:oxygen oxidoreductase activity; F:oxidoreductase activity
FvH4_3g20690	Fvb3: 13746269-13748147	8,477	29,469	1.798	3.202	1.000E−04	3.925E−03	NAC domain-containing protein 72-like	P:GO:0006355; F:GO:0003677	P:regulation of transcription, DNA-templated; F:DNA binding
FvH4_3g23080	Fvb3: 16172270-16173557	12,265	42,580	1.796	3.201	1.000E−04	3.925E−03	probable F-box protein At4g22030		
FvH4_2g14230	Fvb2: 12497953-12499775	98,141	340,186	1.793	3.743	5.000E−05	2.218E−03	uncharacterized protein		
FvH4_6g48240	Fvb6: 36372869-36373469	19,861	68,400	1.784	2.709	1.300E−03	2.498E−02	probable calcium-binding protein CML45	F:GO:0005509	F:calcium ion binding
FvH4_6g38220	Fvb6: 30234816-30235416	17,169	59,051	1.782	2.585	2.100E−03	3.386E−02	centrosomal protein of 83 kDa		
FvH4_5g10890	Fvb5: 6169809-6173697	7,793	26,707	1.777	3.397	5.000E−05	2.218E−03	methionine gamma-lyase	F:GO:0003824; F:GO:0030170	F:catalytic activity; F:pyridoxal phosphate binding
FvH4_5g30060	Fvb5: 20983503-20986619	10,284	34,805	1.759	2.969	4.000E−04	1.118E−02	mRNA turnover protein 4 homolog	P:GO:0042254; C:GO:0005622	P:ribosome biogenesis; C:intracellular
FvH4_6g46000	Fvb6: 35168026-35175316	2,439	8,186	1.747	2.623	1.900E−03	3.175E−02	basic leucine zipper 43	P:GO:0006355; F:GO:0003700	P:regulation of transcription, DNA-templated; F:DNA binding transcription factor activity
FvH4_1g23380	Fvb1: 15219336-15221673	7,673	25,613	1.739	3.350	5.000E−05	2.218E−03	G-type lectin S-receptor-like serine/threonine-protein kinase RLK1	P:GO:0006468; P:GO:0048544; F:GO:0005524; F:GO:0005524; F:GO:0004672; F:GO:0004674	P:protein phosphorylation; P:recognition of pollen; F:ATP binding; F:ATP binding; F:protein kinase activity; F:protein serine/threonine kinase activity
FvH4_2g14260	Fvb2: 12541983-12543225	8,440	28,115	1.736	2.733	1.400E−03	2.610E−02	U-box domain-containing protein 28-like	P:GO:0016567; F:GO:0005488; F:GO:0004842	P:protein ubiquitination; F:binding; F:ubiquitin-protein transferase activity
FvH4_4g18070	Fvb4: 22014435-22016745	9,647	31,760	1.719	3.452	5.000E−05	2.218E−03	uncharacterized protein		
FvH4_6g50160	Fvb6: 37377580-37380341	25,773	84,362	1.711	3.826	5.000E−05	2.218E−03	senescence-associated carboxylesterase 101-like isoform X1	P:GO:0006629	P:lipid metabolic process
FvH4_3g23950	Fvb3: 17038443-17043250	1,718	5,596	1.703	2.484	2.550E−03	3.857E−02	protein TORNADO 1 leucine-rich repeat	F:GO:0005515	F:protein binding
FvH4_7g09470	Fvb7: 9077848-9085705	2,167	7,034	1.699	2.674	1.300E−03	2.498E−02	pentatricopeptide repeat-containing protein At1g02060	P:GO:0008152; F:GO:0003824; F:GO:0005515	P:metabolic process; F:catalytic activity; F:protein binding

FvH4_5g31410	Fvb5: 22320064-22322074	20,626	66,878	1.697	3.536	5.000E−05	2.218E−03	serine/threonine-protein kinase-like protein CCR4	P:GO:0006468; F:GO:0005524; F:GO:0005524; F:GO:0004672	P:protein phosphorylation; F:ATP binding; F:ATP binding; F:protein kinase activity
FvH4_5g23180	Fvb5: 14543166-14546024	5,911	19,107	1.693	2.653	1.650E−03	2.879E−02	probable xyloglucan endotransglucosylase/hydrolase protein 30 (Glycoside hydrolase family 16)	P:GO:0005975; P:GO:0006073; C:GO:0048046; C:GO:0005618; F:GO:0004553; F:GO:0016762	P:carbohydrate metabolic process; P:cellular glucan metabolic process; C:apoplast; C:cell wall; F:hydrolase activity, hydrolyzing O-glycosyl compounds; F:xyloglucan:xyloglucosyl transferase activity
FvH4_3g15530	Fvb3: 9712226-9717873	3,753	12,130	1.692	2.533	2.500E−03	3.820E−02	peptidyl-prolyl cis-trans isomerase CYP59	P:GO:0000413; F:GO:0003676; F:GO:0003676; F:GO:0003755; F:GO:0008270	P:protein peptidyl-prolyl isomerization; F:nucleic acid binding; F:nucleic acid binding; F:peptidyl-prolyl cis-trans isomerase activity; F:zinc ion binding
FvH4_7g09950	Fvb7: 9446888-9451191	2,369	7,598	1.682	2.643	1.500E−03	2.716E−02	pentatricopeptide repeat-containing protein At2g44880	F:GO:0005515	F:protein binding
FvH4_7g28580	Fvb7: 21173358-21175263	7,298	23,265	1.673	2.952	4.000E−04	1.118E−02	receptor-like serine/threonine-protein kinase At4g25390	P:GO:0006468; F:GO:0005524; F:GO:0005524; F:GO:0004672	P:protein phosphorylation; F:ATP binding; F:ATP binding; F:protein kinase activity
FvH4_5g26600	Fvb5: 18048399-18049130	14,042	44,545	1.666	2.375	3.700E−03	4.961E−02	uncharacterized protein		
FvH4_5g21000	Fvb5: 12626246-12634186	1,889	5,974	1.661	2.627	1.350E−03	2.565E−02	uncharacterized protein	P:GO:0006397	P:mRNA processing
FvH4_7g31000	Fvb7: 22461265-22464273	3,325	10,503	1.660	2.463	2.800E−03	4.120E−02	uncharacterized protein		
FvH4_6g00350	Fvb6: 252981-256126	6,207	19,438	1.647	3.080	1.500E−04	5.311E−03	protein kri1		
FvH4_6g35980	Fvb6: 28347563-28349451	11,399	35,592	1.643	2.547	1.400E−03	2.610E−02	RING-H2 finger protein ATL3-like (Zinc finger)		
FvH4_3g22160	Fvb3: 15194955-15198581	5,445	16,757	1.622	2.748	9.500E−04	2.088E−02	protein MOS2	F:GO:0003676	F:nucleic acid binding
FvH4_3g14380	Fvb3: 8858823-8860554	23,091	70,921	1.619	3.509	5.000E−05	2.218E−03	zingipain-2-like	P:GO:0006508; F:GO:0008234	P:proteolysis; F:cysteine-type peptidase activity
FvH4_5g04050	Fvb5: 2408743-2415308	3,996	12,262	1.617	2.732	1.250E−03	2.433E−02	trichohyalin-like		
FvH4_3g24670	Fvb3: 17766524-17771832	2,457	7,536	1.617	2.402	3.000E−03	4.292E−02	poly [ADP-ribose]:ADP-ribose;Poly:[ADP-ribose] polymerase 3 (DNA replication)	F:GO:0006471; F:GO:0003950; F:GO:0003950	P:protein ADP-ribosylation; F:NAD+ ADP-ribosyltransferase activity; F:NAD+ ADP-ribosyltransferase activity
FvH4_2g41060	Fvb2: 29128088-29130611	17,212	52,715	1.615	3.513	5.000E−05	2.218E−03	probable wrky transcription factor 40 isoform X2	P:GO:0006355; F:GO:0003700; F:GO:0043565	P:regulation of transcription, DNA-templated; F:DNA binding transcription factor activity; F:sequence-specific DNA binding
FvH4_4g25060	Fvb4: 26986388-26990956	2,571	7,873	1.615	2.554	2.050E−03	3.342E−02	uncharacterized protein		

FvH4_3g30620	Fvb3: 23966289-23975506	6,947	21,102	1.603	2.503	2.150E−03	3.425E−02	superoxide dismutase	P:GO:0055114; P:GO:0006801; F:GO:0046872; F:GO:0004784	P:oxidation-reduction process; P:superoxide metabolic process; F:metal ion binding; F:superoxide dismutase activity
FvH4_2g40150	Fvb2: 28671382-28672822	5,907	17,886	1.598	2.359	3.550E−03	4.847E−02	anthocyanidin 3-O-glucosyltransferase 5-like	P:GO:0008152; F:GO:0016758	P:metabolic process; F:transferase activity, transferring hexosyl groups
FvH4_7g22820	Fvb7: 17936656-17943623	9,666	29,195	1.595	2.626	1.050E−03	2.206E−02	crocetin glucosyltransferase, chloroplastic-like	P:GO:0008152; F:GO:0016758	P:metabolic process; F:transferase activity, transferring hexosyl groups
FvH4_2g32920	Fvb2: 24828557-24831855	5,933	17,891	1.592	2.679	9.000E−04	2.034E−02	trichohyalin-like		
FvH4_7g04070	Fvb7: 4645233-4646280	21,143	63,375	1.584	3.108	5.000E−05	2.218E−03	probable carboxylesterase 15 (Alpha/beta hydrolase fold-3)	P:GO:0008152; F:GO:0016787	P:metabolic process; F:hydrolase activity
FvH4_2g39030	Fvb2: 28124287-28127033	5,090	15,106	1.569	2.572	1.250E−03	2.433E−02	protein LTV1 homolog		
FvH4_4g23040	Fvb4: 25643428-25647055	7,408	21,878	1.562	2.583	1.950E−03	3.236E−02	GEM-like protein 5		
FvH4_5g03330	Fvb5: 1990710-1991247	28,444	83,952	1.561	2.504	1.350E−03	2.565E−02	uncharacterized protein		
FvH4_6g49580	Fvb6: 37123794-37125405	34,195	100,353	1.553	3.479	5.000E−05	2.218E−03	probable mitochondrial chaperone BCS1-B	F:GO:0005524	F:ATP binding
FvH4_2g40560	Fvb2: 28894033-28900936	3,408	9,977	1.550	2.573	1.400E−03	2.610E−02	cytochrome p450, family 82, subfamily C, polypeptide 4	P:GO:0055114; F:GO:0020037; F:GO:0005506; F:GO:0016705	P:oxidation-reduction process; F:heme binding; F:iron ion binding; F:oxidoreductase activity, acting on paired donors, with incorporation or reduction of molecular oxygen
FvH4_2g07410	Fvb2: 6119730-6121188	15,674	45,864	1.549	3.085	1.000E−04	3.925E−03	allene oxide synthase-like	P:GO:0055114; F:GO:0020037; F:GO:0005506; F:GO:0004497; F:GO:0016705	P:oxidation-reduction process; F:heme binding; F:iron ion binding; F:monooxygenase activity; F:oxidoreductase activity, acting on paired donors, with incorporation or reduction of molecular oxygen
FvH4_7g08350	Fvb7: 8253861-8257399	5,923	17,249	1.542	2.554	1.700E−03	2.957E−02	pentatricopeptide repeat-containing protein At5g16420, mitochondrial		
FvH4_1g17230	Fvb1: 9948828-9952507	3,898	11,338	1.540	2.370	3.400E−03	4.696E−02	acyl-CoA-binding protein	F:GO:0000062	F:fatty-acyl-CoA binding
FvH4_4g26170	Fvb4: 27680689-27681235	50,288	146,231	1.540	2.898	5.000E−05	2.218E−03	uncharacterized protein		
FvH4_4g04250	Fvb4: 3668238-3673373	5,325	15,473	1.539	2.562	1.350E−03	2.565E−02	protein NRT1/ PTR FAMILY 5.2-like	P:GO:0055085; C:GO:0016020; F:GO:0022857	P:transmembrane transport; C:membrane; F:transmembrane transporter activity
FvH4_1g11200	Fvb1: 6116339-6119885	3,883	11,185	1.526	2.454	1.900E−03	3.175E−02	pre-rRNA-processing protein ESF1	C:GO:0005634	C:nucleus

FvH4_1g25750	Fvb1: 17670990-17677657	6,119	17,439	1.511	2.796	3.000E−04	8.972E−03	uncharacterized protein		
FvH4_5g19800	Fvb5: 11637731-11638778	11,079	31,466	1.506	2.344	2.700E−03	4.027E−02	ethylene-responsive transcription factor 5	P:GO:0006355; F:GO:0003677; F:GO:0003700	P:regulation of transcription, DNA-templated; F:DNA binding; F:DNA binding transcription factor activity
FvH4_5g28820	Fvb5: 19946978-19947413	1986,000	700,303	−1.504	−3.069	5.000E−05	2.218E−03	ferredoxin-1-like	P:GO:0022900; F:GO:0051537; F:GO:0009055; F:GO:0051536	P:electron transport chain; F:2 iron, 2 sulfur cluster binding; F:electron transfer activity; F:iron-sulfur cluster binding
FvH4_1g12070	Fvb1: 6594794-6601484	10,311	3,634	−1.505	−2.720	1.050E−03	2.206E−02	kinesin-like protein NACK2	P:GO:0007018; P:GO:0007018; F:GO:0005524; F:GO:0008017; F:GO:0003777	P:microtubule-based movement; P:microtubule-based movement; F:ATP binding; F:microtubule binding; F:microtubule motor activity
FvH4_5g13550	Fvb5: 7671650-7677124	32,108	11,269	−1.511	−3.365	5.000E−05	2.218E−03	protein COBRA-like	P:GO:0016049; P:GO:0010215; C:GO:0031225	P:cell growth; P:cellulose microfibril organization; C:anchored component of membrane
FvH4_3g03790	Fvb3: 2130487-2133928	37,729	13,204	−1.515	−3.027	5.000E−05	2.218E−03	putative PAP-specific phosphatase, mitochondrial	P:GO:0046854	P:phosphatidylinositol phosphorylation
FvH4_6g54460	Fvb6: 39756571-39759126	1003,380	349,880	−1.520	−2.258	2.200E−03	3.482E−02	glyceraldehyde-3-phosphate dehydrogenase A, chloroplastic [	P:GO:0006006; P:GO:0055114; F:GO:0051287; F:GO:0050661; F:GO:0016620	P:glucose metabolic process; P:oxidation-reduction process; F:NAD binding; F:NADP binding; F:oxidoreductase activity, acting on the aldehyde or oxo group of donors, NAD or NADP as acceptor
FvH4_5g15500	Fvb5: 8750872-8751965	133,903	46,681	−1.520	−3.379	5.000E−05	2.218E−03	uncharacterized protein		
FvH4_5g21610	Fvb5: 13039024-13041007	150,967	52,611	−1.521	−3.465	5.000E−05	2.218E−03	glycine cleavage system H protein 3, mitochondrial-like	P:GO:0019464; C:GO:0005960	P:glycine decarboxylation via glycine cleavage system; C:glycine cleavage complex
FvH4_2g15200	Fvb2: 13371427-13382397	17,496	6,097	−1.521	−2.726	6.500E−04	1.640E−02	ACT domain-containing protein ACR3		
FvH4_2g37730	Fvb2: 27397438-27398482	30,922	10,747	−1.525	−2.371	2.300E−03	3.578E−02	shikimate O-hydroxycinnamoyltransferase-like	F:GO:0016747	F:transferase activity, transferring acyl groups other than amino-acyl groups
FvH4_2g27000	Fvb2: 21564557-21568686	163,378	56,354	−1.536	−3.263	5.000E−05	2.218E−03	chlorophyllide a oxygenase, chloroplastic	P:GO:0055114; F:GO:0051537; F:GO:0010277; F:GO:0016491	P:oxidation-reduction process; F:2 iron, 2 sulfur cluster binding; F:chlorophyllide a oxygenase [overall] activity; F:oxidoreductase activity
FvH4_6g31740	Fvb6: 24848099-24849503	823,420	283,559	−1.538	−3.194	1.000E−04	3.925E−03	photosystem I reaction center subunit VI, chloroplastic-like	P:GO:0015979; C:GO:0009522; C:GO:0009538	P:photosynthesis; C:photosystem I; C:photosystem I reaction center
FvH4_5g30340	Fvb5: 21228946-21232762	68,867	23,653	−1.542	−3.423	5.000E−05	2.218E−03	glycerate dehydrogenase	P:GO:0008152; P:GO:0055114; F:GO:0051287; F:GO:0016616	P:metabolic process; P:oxidation-reduction process; F:NAD binding; F:oxidoreductase activity, acting on the CH-OH group of donors, NAD or NADP as acceptor
FvH4_1g29140	Fvb1: 21756709-21766705	35,393	12,136	−1.544	−3.286	5.000E−05	2.218E−03	alpha-L-arabinofuranosidase 1	P:GO:0046373; F:GO:0046556	P:L-arabinose metabolic process; F:alpha-L-arabinofuranosidase activity

FvH4_1g02570	Fvb1: 1441131-1442616	38,050	13,046	−1.544	−2.563	1.100E−03	2.252E−02	36.4 kDa proline-rich protein		
FvH4_5g39010	Fvb5: 28812197-28817206	38,741	13,268	−1.546	−3.479	5.000E−05	2.218E−03	nitrate reductase [NADH]	P:GO:0042128; P:GO:0042128; P:GO:0006809; F:GO:0055114; F:GO:0020037; F:GO:0030151; F:GO:0043546; F:GO:0050464; F:GO:0016491	P:nitrate assimilation; P:nitrate assimilation; P:nitric oxide biosynthetic process; P:oxidation-reduction process; F:heme binding; F:molybdenum ion binding; F:molybdopterin cofactor binding; F:nitrate reductase (NADPH) activity; F:oxidoreductase activity;
FvH4_6g09980	Fvb6: 5928404-5929569	206,979	70,856	−1.547	−3.535	5.000E−05	2.218E−03	non-specific lipid-transfer protein 1-like isoform X1		
FvH4_2g14790	Fvb2: 13006655-13015170	12,261	4,196	−1.547	−2.450	2.550E−03	3.857E−02	probable glucuronosyltransferase Os02g0520750	P:GO:0015979; P:GO:0006486; C:GO:0019898; C:GO:0009523; C:GO:0009654; F:GO:0005509; F:GO:0016757	P:photosynthesis; P:protein glycosylation; C:extrinsic component of membrane; C:photosystem II; C:photosystem II oxygen evolving complex; F:calcium ion binding; F:transferase activity, transferring glycosyl groups
FvH4_1g09040	Fvb1: 4778659-4780612	50,894	17,358	−1.552	−2.920	2.000E−04	6.539E−03	chlorophyll a-b binding protein, chloroplastic	P:GO:0009765; C:GO:0016020	P:photosynthesis, light harvesting; C:membrane
FvH4_6g16770	Fvb6: 10698240-10699604	128,305	43,686	−1.554	−3.517	5.000E−05	2.218E−03	aquaporin TIP1-1-like	P:GO:0055085; C:GO:0016020; F:GO:0015267	P:transmembrane transport; C:membrane; F:channel activity
FvH4_7g11190	Fvb7: 10442660-10443056	180,168	61,312	−1.555	−2.680	7.500E−04	1.786E−02	putative lipid-binding protein At4g00165		
FvH4_3g19430	Fvb3: 12597964-12600101	124,918	42,426	−1.558	−3.159	5.000E−05	2.218E−03	30S ribosomal protein S20, chloroplastic	P:GO:0006412; C:GO:0005622; C:GO:0005840; F:GO:0003723; F:GO:0003735	P:translation; C:intracellular; C:ribosome; F:RNA binding; F:structural constituent of ribosome
FvH4_6g44370	Fvb6: 34191144-34193039	438,605	148,636	−1.561	−3.293	5.000E−05	2.218E−03	cytochrome b6-f complex iron-sulfur subunit, chloroplastic	P:GO:0055114; P:GO:0015979; C:GO:0016020; C:GO:0042651; F:GO:0051537; F:GO:0045158; F.GO:0016491; F:GO:0016679; F:GO:0009496;	P:oxidation-reduction process; P:photosynthesis; C:membrane; C:thylakoid membrane; F:2 iron, 2 sulfur cluster binding; F:electron transporter, transferring electrons within cytochrome b6/f complex of photosystem II activity; F:oxidoreductase activity; F:oxidoreductase activity, acting on diphenols and related substances as donors; F:plastoquinol--plastocyanin reductase activity;
FvH4_5g15290	Fvb5: 8652214-8656148	11,263	3,812	−1.563	−2.351	3.500E−03	4.790E−02	protein IQ-DOMAIN 31	F:GO:0005515	F:protein binding
FvH4_2g20470	Fvb2: 17180656-17182221	78,725	26,473	−1.572	−3.227	5.000E−05	2.218E−03	photosystem II reaction center Psb28 protein	P:GO:0015979; C:GO:0016020; C:GO:0009523; C:GO:0009654	P:photosynthesis; C:membrane; C:photosystem II; C:photosystem II oxygen evolving complex

FvH4_7g31650	Fvb7: 22833443-22837316	32,274	10,851	−1.573	−3.304	5.000E−05	2.218E−03	probable pectinesterase/pectinesterase inhibitor 34	P:GO:0042545; C:GO:0005618; F:GO:0004857; F:GO:0030599	P:cell wall modification; C:cell wall; F:enzyme inhibitor activity; F:pectinesterase activity
FvH4_4g16670	Fvb4: 20537377-20543743	28,679	9,608	−1.578	−3.384	5.000E−05	2.218E−03	pyruvate, phosphate dikinase 2	P:GO:0016310; P:GO:0016310; P:GO:0006090; F:GO:0005524; F:GO:0005524; F:GO:0003824; F:GO:0003824; F:GO:0016301; F:GO:0050242; F:GO:0016772	P:phosphorylation; P:phosphorylation; P:pyruvate metabolic process; F:ATP binding; F:ATP binding; F:catalytic activity; F:catalytic activity; F:kinase activity; F:pyruvate, phosphate dikinase activity; F:transferase activity, transferring phosphorus-containing groups
FvH4_1g04250	Fvb1: 2248419-2252439	55,401	18,556	−1.578	−3.464	5.000E−05	2.218E−03	palmitoyl-acyl carrier protein thioesterase, chloroplastic	P:GO:0006633; F:GO:0016790	P:fatty acid biosynthetic process; F:thiolester hydrolase activity
FvH4_7g05600	Fvb7: 5873291-5875660	53,418	17,875	−1.579	−2.929	2.000E−04	6.539E−03	Alpha/beta hydrolase fold-1		
FvH4_5g07070	Fvb5: 4165279-4173530	29,256	9,784	−1.580	−2.562	2.050E−03	3.342E−02	haloacid dehalogenase-like hydrolase domain-containing protein At3g48420	P:GO:0008152; F:GO:0016787	P:metabolic process; F:hydrolase activity
FvH4_5g31620	Fvb5: 22776388-22777671	57,161	19,093	−1.582	−2.907	1.500E−04	5.311E−03	carboxymethylenebutenolidase homolog isoform X1	F:GO:0016787	F:hydrolase activity
FvH4_7g06810	Fvb7: 6818389-6823978	36,678	12,221	−1.586	−3.033	5.000E−05	2.218E−03	mitochondrial carnitine/acylcarnitine carrier-like protein		
FvH4_3g01310	Fvb3: 667879-669731	138,811	46,153	−1.589	−3.603	5.000E−05	2.218E−03	plastid-lipid-associated protein, chloroplastic		
FvH4_6g33070	Fvb6: 26130469-26136978	16,143	5,359	−1.591	−3.333	5.000E−05	2.218E−03	ABC transporter C family member 8-like	P:GO:0055085; C:GO:0016021; F:GO:0005524; F:GO:0016887; F:GO:0042626	P:transmembrane transport; C:integral component of membrane; F:ATP binding; F:ATPase activity; F:ATPase activity, coupled to transmembrane movement of substances
FvH4_3g26310	Fvb3: 19275091-19277530	59,918	19,885	−1.591	−3.464	5.000E−05	2.218E−03	protein FAF-like, chloroplastic		
FvH4_6g06940	Fvb6: 4183710-4194757	15,736	5,198	−1.598	−2.879	2.500E−04	7.689E−03	phospholipase D p1	P:GO:0048017; P:GO:0006654; F:GO:0003824; F:GO:0035091; F:GO:0004630	P:inositol lipid-mediated signaling; P:phosphatidic acid biosynthetic process; F:catalytic activity; F:phosphatidylinositol binding; F:phospholipase D activity
FvH4_2g37370	Fvb2: 27225179-27228523	20,687	6,827	−1.599	−2.542	1.100E−03	2.252E−02	F-box protein SKIP31	F:GO:0005515	F:protein binding
FvH4_4g34390	Fvb4: 32205730-32212875	12,036	3,956	−1.605	−2.481	2.150E−03	3.425E−02	carbonic anhydrase 2 isoform X1		
FvH4_5g09430	Fvb5: 5471698-5473025	270,878	88,991	−1.606	−3.544	5.000E−05	2.218E−03	magnesium protoporphyrin IX methyltransferase, chloroplasti	P:GO:0015995; F:GO:0046406	P:chlorophyll biosynthetic process; F:magnesium protoporphyrin IX methyltransferase activity

FvH4_6g35830	Fvb6: 28260215-28265168	25,561	8,381	−1.609	−3.107	5.000E−05	2.218E−03	pectinesterase-like	P:GO:0042545; C:GO:0005618; F:GO:0004857; F:GO:0030599	P:cell wall modification; C:cell wall; F:enzyme inhibitor activity; F:pectinesterase activity
FvH4_5g14320	Fvb5: 8096868-8100518	33,636	11,021	−1.610	−3.130	5.000E−05	2.218E−03	heparanase-like protein 1	C:GO:0016020; F:GO:0016798	C:membrane; F:hydrolase activity, acting on glycosyl bonds
FvH4_5g10820	Fvb5: 6116909-6119363	33,673	10,959	−1.620	−2.968	2.500E−04	7.689E−03	beta-carotene isomerase D27, chloroplastic isoform X2		
FvH4_4g26990	Fvb4: 28167295-28173751	28,053	9,082	−1.627	−3.639	5.000E−05	2.218E−03	zinc finger protein-related	F:GO:0008270	F:zinc ion binding
FvH4_2g37410	Fvb2: 27272666-27281273	34,122	11,040	−1.628	−3.440	5.000E−05	2.218E−03	1,4-alpha-glucan-branching enzyme 1, chloroplastic/amyloplastic-like	P:GO:0005975; P:GO:0005975; P:GO:0005978; F:GO:0003844; F:GO:0003824; F:GO:0043169; F:GO:0004553	P:carbohydrate metabolic process; P:carbohydrate metabolic process; P:glycogen biosynthetic process; F:1,4-alpha-glucan branching enzyme activity; F:catalytic activity; F:cation binding; F:hydrolase activity, hydrolyzing O-glycosyl compounds ;
FvH4_1g13000	Fvb1: 7169239-7172204	27,447	8,875	−1.629	−2.936	3.500E−04	1.016E−02	pheophytinase, chloroplastic		
FvH4_5g37940	Fvb5: 27986598-27988238	54,394	17,579	−1.630	−2.921	3.000E−04	8.972E−03	pollen-specific protein C13, Allergen	C:GO:0005615	C:extracellular space
FvH4_3g38780	Fvb3: 33059784-33062590	21,236	6,842	−1.634	−2.835	3.500E−04	1.016E−02	omega-3 fatty acid desaturase, endoplasmic reticulum	P:GO:0006629; P:GO:0055114; F:GO:0016717	P:lipid metabolic process; P:oxidation-reduction process; F:oxidoreductase activity, acting on paired donors, with oxidation of a pair of donors resulting in the reduction of molecular oxygen to two molecules of water
FvH4_1g05310	Fvb1: 2813698-2820474	24,069	7,750	−1.635	−3.343	5.000E−05	2.218E−03	Fe-S cluster assembly factor	F:GO:0005524	F:ATP binding
FvH4_6g29130	Fvb6: 22468650-22469271	80,749	25,906	−1.640	−2.768	7.500E−04	1.786E−02	uncharacterized protein		
FvH4_7g23490	Fvb7: 18302314-18307888	21,240	6,792	−1.645	−2.993	4.500E−04	1.218E−02	folylpolyglutamate synthase	P:GO:0009058; P:GO:0009058; F:GO:0009396; F:GO:0005524; F:GO:0016874; F:GO:0004326	P:biosynthetic process; P:biosynthetic process; P:folic acid-containing compound biosynthetic process; F:ATP binding; F:ligase activity; F:tetrahydrofolylpolyglutamate synthase activity
FvH4_5g25760	Fvb5: 17250900-17253991	241,395	76,976	−1.649	−3.492	5.000E−05	2.218E−03	glyceraldehyde-3-phosphate dehydrogenase B, chloroplastic	P:GO:0006006; P:GO:0055114; F:GO:0051287; F:GO:0050661; F:GO:0016620	P:glucose metabolic process; P:oxidation-reduction process; F:NAD binding; F:NADP binding; F:oxidoreductase activity, acting on the aldehyde or oxo group of donors, NAD or NADP as acceptor
FvH4_5g26660	Fvb5: 18111515-18112011	229,426	73,139	−1.649	−3.372	5.000E−05	2.218E−03	uncharacterized protein		
FvH4_1g28120	Fvb1: 19967370-19970423	32,563	10,380	−1.649	−3.195	2.000E−04	6.539E−03	glutamate dehydrogenase 1	P:GO:0006520; P:GO:0055114; F:GO:0016491; F:GO:0016639	P:cellular amino acid metabolic process; P:oxidation-reduction process; F:oxidoreductase activity; F:oxidoreductase activity, acting on the CH-NH2 group of donors, NAD or NADP as acceptor

FvH4_7g04090	Fvb7: 4675655-4678077	28,071	8,942	−1.650	−2.866	5.000E−04	1.311E−02	WAT1-related protein	C:GO:0016021; C:GO:0016021; C:GO:0016020; C:GO:0022857	C:integral component of membrane; C:integral component of membrane; C:membrane; F:transmembrane transporter activity
FvH4_4g12300	Fvb4: 16010848-16013334	20,001	6,360	−1.653	−2.790	7.000E−04	1.719E−02	vacuolar amino acid transporter 1 isoform X2		
FvH4_3g05680	Fvb3: 3313507-3317532	38,191	12,109	−1.657	−3.095	2.000E−04	6.539E−03	uncharacterized protein		
FvH4_2g27940	Fvb2: 22069706-22073785	24,899	7,878	−1.660	−3.392	5.000E−05	2.218E−03	probable receptor-like serine/threonine-protein kinase	P:GO:0006468; F:GO:0005524; F:GO:0005524; F:GO:0004672	P:protein phosphorylation; F:ATP binding; F:ATP binding; F:protein kinase activity
FvH4_6g48590	Fvb6: 36571330-36572596	39,213	12,345	−1.667	−2.517	2.500E−03	3.820E−02	gibberellin-regulated protein 6-like		
FvH4_4g00270	Fvb4: 274901-281225	23,428	7,370	−1.669	−3.288	5.000E−05	2.218E−03	uncharacterized aarF domain-containing protein kinase At1g71810, chloroplastic	P:GO:0006468; F:GO:0005524; F:GO:0004672	P:protein phosphorylation; F:ATP binding; F:protein kinase activity
FvH4_5g17300	Fvb5: 9876630-9880876	35,947	11,307	−1.669	−3.352	5.000E−05	2.218E−03	phosphomethylethanolamine N-methyltransferase-like	P:GO:0008152; P:GO:0006656; F:GO:0008168; F:GO:0000234	P:metabolic process; P:phosphatidylcholine biosynthetic process; F:methyltransferase activity; F:phosphoethanolamine N-methyltransferase activity
FvH4_5g21090	Fvb5: 12674464-12677534	35,381	11,125	−1.669	−3.147	1.000E−04	3.925E−03	mannose-6-phosphate isomerase 2-like	P:GO:0009298; P:GO:0005975; F:GO:0004476; F:GO:0004476; F:GO:0008270	P:GDP-mannose biosynthetic process; P:carbohydrate metabolic process; F:mannose-6-phosphate isomerase activity; F:mannose-6-phosphate isomerase activity; F:zinc ion binding
FvH4_2g38670	Fvb2: 27909830-27912670	19,262	6,040	−1.673	−2.839	8.000E−04	1.868E−02	endoglucanase 10-like	P:GO:0005975; F:GO:0003824; F:GO:0004553	P:carbohydrate metabolic process; F:catalytic activity; F:hydrolase activity, hydrolyzing O-glycosyl compounds
FvH4_2g19970	Fvb2: 16837232-16839096	28,199	8,826	−1.676	−2.453	2.450E−03	3.763E−02	protein LOW PSII accumulation 1, chloroplastic		
FvH4_4g23890	Fvb4: 26217026-26220091	16,881	5,277	−1.678	−2.550	2.300E−03	3.578E−02	sulfite oxidase-like isoform X2	P:GO:0042128; P:GO:0055114; F:GO:0030151; F:GO:0016491	P:nitrate assimilation; P:oxidation-reduction process; F:molybdenum ion binding; F:oxidoreductase activity
FvH4_6g00530	Fvb6: 323097-325385	1868,310	583,708	−1.678	−2.331	2.950E−03	4.257E−02	photosystem I reaction center subunit XI, chloroplastic	P:GO:0015979; C:GO:0009522; C:GO:0009538	P:photosynthesis; C:photosystem I; C:photosystem I reaction center
FvH4_4g23750	Fvb4: 26130750-26132548	2039,370	637,039	−1.679	−2.200	2.700E−03	4.027E−02	chlorophyll a-b binding protein, chloroplastic	P:GO:0009765; C:GO:0016020	P:photosynthesis, light harvesting; C:membrane
FvH4_4g22640	Fvb4: 25375451-25377058	39,895	12,454	−1.680	−2.991	1.000E−04	3.925E−03	uncharacterized protein LOC101303873		
FvH4_6g51010	Fvb6: 37881988-37887165	25,824	8,053	−1.681	−3.326	5.000E−05	2.218E−03	phosphoenolpyruvate/phosphate translocator 2, chloroplastic	P:GO:0030488; P:GO:0055085; C:GO:0016021; C:GO:0031515; F:GO:0016429; F:GO:0022857	P:tRNA methylation; P:transmembrane transport; C:integral component of membrane; C:tRNA (m1A) methyltransferase complex; F:tRNA (adenine-N1-)-methyltransferase activity; F:transmembrane transporter activity

FvH4_2g13890	Fvb2: 12167935-12172009	37,404	11,661	−1.682	−3.289	1.000E−04	3.925E−03	fructose-1,6-bisphosphatase, cytosolic	P:GO:0005975; F:GO:0042132; F:GO:0016791; F:GO:0042578	P:carbohydrate metabolic process; F:fructose 1,6-bisphosphate 1-phosphatase activity; F:phosphatase activity; F:phosphoric ester hydrolase activity
FvH4_7g24240	Fvb7: 18726677-18731259	10,174	3,158	−1.688	−2.734	4.500E−04	1.218E−02	probable lrr receptor-like serine/threonine-protein kinase At3g47570	P:GO:0006468; F:GO:0005524; F:GO:0005515; F:GO:0004672	P:protein phosphorylation; F:ATP binding; F:protein binding; F:protein kinase activity
FvH4_7g23380	Fvb7: 18260295-18263189	50,533	15,677	−1.689	−3.506	5.000E−05	2.218E−03	mannose-1-phosphate guanylyltransferase 1	P:GO:0009058; F:GO:0016779	P:biosynthetic process; F:nucleotidyltransferase activity
FvH4_6g29320	Fvb6: 22610901-22616004	240,984	74,598	−1.692	−3.270	5.000E−05	2.218E−03	pyrophosphate-energized vacuolar membrane proton pump-like	P:GO:0015992; C:GO:0016020; F:GO:0009678; F:GO:0004427	P:proton transport; C:membrane; F:hydrogen-translocating pyrophosphatase activity; F:inorganic diphosphatase activity
FvH4_5g14950	Fvb5: 8458428-8459503	60,874	18,842	−1.692	−2.972	6.000E−04	1.520E−02	gibberellin-regulated protein 6		
FvH4_7g18050	Fvb7: 15175409-15178980	15,976	4,940	−1.693	−2.869	2.000E−04	6.539E−03	cucumisin-like	P:GO:0006508; F:GO:0004252	P:proteolysis; F:serine-type endopeptidase activity
FvH4_1g21630	Fvb1: 13591226-13595458	25,161	7,780	−1.693	−2.565	2.300E−03	3.578E−02	photosynthetic NDH subunit of lumenal location 4, chloroplastic		
FvH4_6g08370	Fvb6: 4946527-4949032	17,802	5,453	−1.707	−2.568	1.450E−03	2.666E−02	S-adenosylmethionine synthase 1-like	P:GO:0006556; F:GO:0005524; F:GO:0004478	P:S-adenosylmethionine biosynthetic process; F:ATP binding; F:methionine adenosyltransferase activity
FvH4_4g15260	Fvb4: 18876811-18877429	3428,450	1050,130	−1.707	−2.689	9.500E−04	2.088E−02	photosystem I reaction center subunit II, chloroplastic-like	P:GO:0015979; C:GO:0009522; C:GO:0009538	P:photosynthesis; C:photosystem I; C:photosystem I reaction center
FvH4_2g38630	Fvb2: 27898398-27899997	28,636	8,742	−1.712	−2.401	2.050E−03	3.342E−02	protein disulfide-isomerase LQY1		
FvH4_4g25450	Fvb4: 27213930-27219353	159,072	48,483	−1.714	−3.767	5.000E−05	2.218E−03	glutamate--glyoxylate aminotransferase 2	P:GO:0009058; F:GO:0003824; F:GO:0030170	P:biosynthetic process; F:catalytic activity; F:pyridoxal phosphate binding
FvH4_3g02090	Fvb3: 1061066-1063146	792,176	240,868	−1.718	−2.854	3.500E−04	1.016E−02	peroxidase 42	P:GO:0055114; P:GO:0006979; F:GO:0020037; F:GO:0004601	P:oxidation-reduction process; P:response to oxidative stress; F:heme binding; F:peroxidase activity
FvH4_3g11800	Fvb3: 6971526-6972286	2788,440	843,449	−1.725	−2.634	7.500E−04	1.786E−02	photosystem I reaction center subunit III, chloroplastic	P:GO:0015979; C:GO:0009522; C:GO:0009538	P:photosynthesis; C:photosystem I; C:photosystem I reaction center
FvH4_5g21490	Fvb5: 12951593-12956410	18,922	5,720	−1.726	−3.100	2.000E−04	6.539E−03	protein NRT1/ PTR	P:GO:0055085; C:GO:0016020; F:GO:0022857	P:transmembrane transport; C:membrane; F:transmembrane transporter activity
FvH4_3g02920	Fvb3: 1561440-1563015	974,029	294,374	−1.726	−3.097	5.000E−05	2.218E−03	oxygen-evolving enhancer protein 3-2, chloroplastic	P:GO:0015979; C:GO:0019898; C:GO:0009523; C:GO:0009654; F:GO:0005509	P:photosynthesis; C:extrinsic component of membrane; C:photosystem II; C:photosystem II oxygen evolving complex; F:calcium ion binding
FvH4_6g40570	Fvb6: 32039296-32044774	25,957	7,825	−1.730	−3.454	1.000E−04	3.925E−03	uncharacterized aarF domain-containing protein kinase At1g79600, chloroplastic		

FvH4_7g27470	Fvb7: 20513141-20513822	56,331	16,963	−1.732	−2.668	1.450E−03	2.666E−02	peroxisomal membrane protein 11B	P:GO:0016559; C:GO:0005779	P:peroxisome fission; C:integral component of peroxisomal membrane
FvH4_2g10390	Fvb2: 9250051-9252469	1149,010	344,089	−1.740	−2.543	2.100E−03	3.386E−02	fructose-bisphosphate aldolase 1, chloroplastic	P:GO:0006096; F:GO:0003824; F:GO:0004332	P:glycolytic process; F:catalytic activity; F:fructose-bisphosphate aldolase activity
FvH4_3g43290	Fvb3: 36141244-36147616	48,350	14,469	−1.741	−2.995	4.500E−04	1.218E−02	uncharacterized protein		
FvH4_6g23800	Fvb6: 17888285-17894115	286,526	85,674	−1.742	−2.817	2.000E−04	6.539E−03	magnesium-chelatase subunit ChlH, chloroplastic	P:GO:0009058; P:GO:0015995; F:GO:0016851	P:biosynthetic process; P:chlorophyll biosynthetic process; F:magnesium chelatase activity
FvH4_3g32630	Fvb3: 27933522-27939963	11,574	3,447	−1.748	−2.812	8.500E−04	1.951E−02	beta-galactosidase 8	P:GO:0005975; F:GO:0030246; F.GO:0004553	P:carbohydrate metabolic process; F:carbohydrate binding; F:hydrolase activity, hydrolyzing O-glycosyl compounds
FvH4_7g22420	Fvb7: 17672129-17676289	38,639	11,418	−1.759	−3.332	5.000E−05	2.218E−03	epsin-3-like		
FvH4_2g36990	Fvb2: 26997371-27000518	71,643	21,160	−1.759	−3.945	5.000E−05	2.218E−03	3-ketoacyl-CoA synthase 10	P:GO:0006633; P:GO:0008152; C:GO:0016020; F:GO:0003824; F:GO:0016747	P:fatty acid biosynthetic process; P:metabolic process; C:membrane; F:catalytic activity; F:transferase activity, transferring acyl groups other than amino-acyl groups
FvH4_5g08480	Fvb5: 4899806-4902107	18,594	5,487	−1.761	−2.813	1.000E−03	2.150E−02	non-specific phospholipase C6	P:GO:0008152; F:GO:0003824; F:GO:0016788	P:metabolic process; F:catalytic activity; F:hydrolase activity, acting on ester bonds
FvH4_3g15380	Fvb3: 9556723-9560275	59,283	17,493	−1.761	−3.698	5.000E−05	2.218E−03	sedoheptulose-1,7-bisphosphatase, chloroplastic-like	P:GO:0005975; F:GO:0016791; F:GO:0042578	P:carbohydrate metabolic process; F:phosphatase activity; F:phosphoric ester hydrolase activity
FvH4_2g23540	Fvb2: 19298527-19303102	15,085	4,423	−1.770	−2.895	8.000E−04	1.868E−02	mitochondrial ribosome-associated GTPase 1	F:GO:0005525	F:GTP binding
FvH4_6g33030	Fvb6: 26088508-26090344	19,724	5,784	−1.770	−2.526	2.900E−03	4.205E−02	inorganic pyrophosphatase 1-like	F:GO:0016791	F:phosphatase activity
FvH4_1g24360	Fvb1: 16228411-16233750	475,949	139,286	−1.773	−2.824	5.500E−04	1.421E−02	probable polygalacturonase	P:GO:0005975; F:GO:0004650	P:carbohydrate metabolic process; F:polygalacturonase activity
FvH4_3g38370	Fvb3: 32785883-32789835	31,327	9,148	−1.776	−3.843	5.000E−05	2.218E−03	E3 ubiquitin-protein ligase CHFR		
FvH4_3g29980	Fvb3: 23159280-23164945	33,465	9,765	−1.777	−3.723	5.000E−05	2.218E−03	glucomannan 4-beta-mannosyltransferase 2		
FvH4_3g00400	Fvb3: 212651-216587	99,526	28,953	−1.781	−3.890	5.000E−05	2.218E−03	oligopeptide transporter 3	P:GO:0055085	P:transmembrane transport
FvH4_6g28220	Fvb6: 21723358-21729860	37,777	10,988	−1.782	−3.379	5.000E−05	2.218E−03	protein ASPARTIC PROTEASE IN GUARD CELL 2	P:GO:0006508; F:GO:0004190	P:proteolysis; F:aspartic-type endopeptidase activity
FvH4_3g01370	Fvb3: 694216-695972	90,959	26,355	−1.787	−3.720	5.000E−05	2.218E−03	uncharacterized protein		
FvH4_5g21680	Fvb5: 13094291-13096030	26,054	7,548	−1.787	−2.654	1.150E−03	2.326E−02	uncharacterized protein		

FvH4_7g30120	Fvb7: 22019432-22044391	16,145	4,661	−1.792	−3.981	5.000E−05	2.218E−03	ferric reduction oxidase 4	P:GO:0055114; F:GO:0016491	P:oxidation-reduction process; F:oxidoreductase activity
FvH4_4g11930	Fvb4: 15646302-15649061	198,154	56,975	−1.798	−3.987	5.000E−05	2.218E−03	chitinase-like protein 1	P:GO:0005975; P:GO:0016998; P:GO:0006032; F:GO:0004568	P:carbohydrate metabolic process; P:cell wall macromolecule catabolic process; P:chitin catabolic process; F:chitinase activity
FvH4_5g28280	Fvb5: 19440578-19445104	12,812	3,673	−1.803	−2.649	1.750E−03	3.000E−02	prolycopene isomerase, chloroplastic isoform X1	P:GO:0055114; F:GO:0016491	P:oxidation-reduction process; F:oxidoreductase activity
FvH4_2g03130	Fvb2: 2426355-2428013	80,994	23,106	−1.810	−2.961	7.000E−04	1.719E−02	uncharacterized protein		
FvH4_7g17340	Fvb7: 14759798-14760392	82,832	23,591	−1.812	−2.492	2.900E−03	4.205E−02	auxin-induced protein X15-like	P:GO:0009733	P:response to auxin
FvH4_1g17630	Fvb1: 10263973-10266658	33,804	9,459	−1.838	−3.211	1.500E−04	5.311E−03	mitochondrial outer membrane protein porin 2-like	P:GO:0098656; P:GO:0055085; C:GO:0005741; F:GO:0008308	P:anion transmembrane transport; P:transmembrane transport; C:mitochondrial outer membrane; F:voltage-gated anion channel activity
FvH4_7g27620	Fvb7: 20584463-20589489	22,471	6,276	−1.840	−3.200	5.000E−05	2.218E−03	trehalose-phosphate phosphatase A	P:GO:0005992; F:GO:0003824	P:trehalose biosynthetic process; F:catalytic activity
FvH4_3g16520	Fvb3: 10424429-10432272	439,758	122,828	−1.840	−3.420	5.000E−05	2.218E−03	peroxisomal (S)-2-hydroxy-acid oxidase isoform X1	P:GO:0055114; F:GO:0010181; F:GO:0003824; F:GO:0016491; F:GO:0016491	P:oxidation-reduction process; F:FMN binding; F:catalytic activity; F:oxidoreductase activity; F:oxidoreductase activity
FvH4_1g24510	Fvb1: 16350441-16358787	13,476	3,756	−1.843	−2.498	3.600E−03	4.876E−02	histone deacetylase 14 isoform X1		
FvH4_5g10570	Fvb5: 6007489-6008918	98,157	27,345	−1.844	−3.967	5.000E−05	2.218E−03	uncharacterized protein		
FvH4_6g38900	Fvb6: 30775176-30776861	1601,740	445,876	−1.845	−2.854	4.000E−04	1.118E−02	oxygen-evolving enhancer protein 2, chloroplastic	P:GO:0015979; C:GO:0019898; C:GO:0009523; C:GO:0009654; F:GO:0005509	P:photosynthesis; C:extrinsic component of membrane; C:photosystem II; C:photosystem II oxygen evolving complex; F:calcium ion binding
FvH4_6g09130	Fvb6: 5410099-5411342	38,523	10,682	−1.851	−3.010	5.000E−04	1.311E−02	uncharacterized protein		
FvH4_4g06760	Fvb4: 6090694-6093633	54,166	14,987	−1.854	−3.443	5.000E−05	2.218E−03	thioredoxin X, chloroplastic	P.GO:0045454; P:GO:0006662; F:GO:0015035	P:cell redox homeostasis; P:glycerol ether metabolic process; F:protein disulfide oxidoreductase activity
FvH4_6g00380	Fvb6: 264234-265586	512,877	141,756	−1.855	−4.159	5.000E−05	2.218E−03	thioredoxin F-type, chloroplastic-like isoform X2	P:GO:0045454; P:GO:0006662; F:GO:0015035	P:cell redox homeostasis; P:glycerol ether metabolic process; F:protein disulfide oxidoreductase activity
FvH4_1g17000	Fvb1: 9837644-9840993	82,416	22,582	−1.868	−4.090	5.000E−05	2.218E−03	metal transporter Nramp3	P:GO:0030001; C:GO:0016020; F:GO:0046873	P:metal ion transport; C:membrane; F:metal ion transmembrane transporter activity
FvH4_5g27150	Fvb5: 18417464-18422984	54,693	14,942	−1.872	−4.163	5.000E−05	2.218E−03	ferric reduction oxidase 7, chloroplastic	P:GO:0055114; C:GO:0016020; F:GO:0016491	P:oxidation-reduction process; C:membrane; F:oxidoreductase activity

FvH4_6g38930	Fvb6: 30788402-30792522	35,804	9,764	−1.875	−3.466	5.000E−05	2.218E−03	uncharacterized oxidoreductase At1g06690, chloroplastic	P:GO:0055114; F:GO:0016491	P:oxidation-reduction process; F:oxidoreductase activity
FvH4_2g37890	Fvb2: 27502908-27503412	2514,610	684,397	−1.877	−3.549	5.000E−05	2.218E−03	plastocyanin, chloroplastic	F:GO:0005507; F:GO:0009055	F:copper ion binding; F:electron transfer activity
FvH4_2g33340	Fvb2: 25038066-25044026	60,644	16,472	−1.880	−4.228	5.000E−05	2.218E−03	subtilisin-like protease	P:GO:0006508; F:GO:0004252	P:proteolysis; F:serine-type endopeptidase activity
FvH4_2g31940	Fvb2: 24333646-24339659	515,382	138,351	−1.897	−2.462	1.850E−03	3.127E−02	glycine dehydrogenase (decarboxylating), mitochondrial	P:GO:0006546; P:GO:0006544; P.GO:0055114; F:GO:0003824; F:GO:0004375	P:glycine catabolic process; P:glycine metabolic process; P:oxidation-reduction process; F:catalytic activity; F:glycine dehydrogenase (decarboxylating) activity
FvH4_7g19750	Fvb7: 16227980-16230030	2292,040	610,382	−1.909	−2.527	1.100E−03	2.252E−02	chlorophyll a-b binding protein 6, chloroplastic	P:GO:0009765; C:GO:0016020	P:photosynthesis, light harvesting; C:membrane
FvH4_6g32440	Fvb6: 25477938-25478742	31,877	8,473	−1.912	−2.496	3.600E−03	4.876E−02	chlorophyll a-b binding protein of LHCII type 1-like	P:GO:0009765; C:GO:0016020	P:photosynthesis, light harvesting; C:membrane
FvH4_5g33740	Fvb5: 24430492-24436620	21,757	5,744	−1.921	−3.551	5.000E−05	2.218E−03	phosphoenolpyruvate carboxykinase [ATP]	P:GO:0006094; P:GO:0006094; F:GO:0005524; F:GO:0004612; F:GO:0004611; F:GO:0017076	P:gluconeogenesis; P:gluconeogenesis; F:ATP binding; F:phosphoenolpyruvate carboxykinase (ATP) activity; F:phosphoenolpyruvate carboxykinase activity; F:purine nucleotide binding
FvH4_6g41950	Fvb6: 32844751-32853444	9,428	2,485	−1.923	−2.514	3.700E−03	4.961E−02	triacylglycerol lipase 2-like	P:GO:0006629	P:lipid metabolic process
FvH4_3g23310	Fvb3: 16385272-16388525	92,712	24,416	−1.925	−4.317	5.000E−05	2.218E−03	GDP-L-galactose phosphorylase 2	F:GO:0080048	F:GDP-D-glucose phosphorylase activity
FvH4_2g34230	Fvb2: 25472803-25475107	69,852	18,271	−1.935	−4.301	5.000E−05	2.218E−03	subtilisin-like protease	P:GO:0006508; F:GO:0004252	P:proteolysis; F:serine-type endopeptidase activity
FvH4_5g37970	Fvb5: 28007504-28008957	82,997	21,482	−1.950	−3.835	5.000E−05	2.218E−03	uncharacterized protein		
FvH4_3g29440	Fvb3: 22515643-22518380	75,508	19,479	−1.955	−4.308	5.000E−05	2.218E−03	zinc transporter 11-like	P:GO:0030001; P:GO:0055085; C:GO:0016020; F:GO:0046873	P:metal ion transport; P:transmembrane transport; C:membrane; F:metal ion transmembrane transporter activity
FvH4_2g02330	Fvb2: 1915610-1919341	15,060	3,842	−1.971	−2.540	3.150E−03	4.464E−02	uncharacterized protein		
FvH4_2g05530	Fvb2: 4568048-4570195	163,365	41,639	−1.972	−4.372	5.000E−05	2.218E−03	leucine-rich repeat (lrr) family protein		
FvH4_3g44350	Fvb3: 36986582-36989613	37,351	9,514	−1.973	−3.612	5.000E−05	2.218E−03	probable plastid-lipid-associated protein 12, chloroplastic isoform X1		
FvH4_2g02490	Fvb2: 1986822-1989446	102,924	26,209	−1.973	−4.165	5.000E−05	2.218E−03	malate dehydrogenase, glyoxysomal isoform X2	P:GO:0005975; P:GO:0019752; P:GO:0006108;	P:carbohydrate metabolic process; P:carboxylic acid metabolic process; P:malate metabolic process; P:oxidation-reduction process; P:tricarboxylic acid cycle; F:L-malate dehydrogenase activity; F:catalytic activity; F:malate dehydrogenase activity;

									P:GO:0055114; P:GO:0006099; F:GO:0030060; F:GO:0003824; F:GO:0016615; F:GO:0016491; F:GO:0016616	F:oxidoreductase activity; F:oxidoreductase activity, acting on the CH-OH group of donors, NAD or NADP as acceptor
FvH4_5g02700	Fvb5: 1623401-1625033	17,668	4,466	−1.984	−2.826	7.500E−04	1.786E−02	cytochrome p450 86A7	P:GO:0055114; F:GO:0020037; F:GO:0005506; F:GO:0016705	P:oxidation-reduction process; F:heme binding; F:iron ion binding; F:oxidoreductase activity, acting on paired donors, with incorporation or reduction of molecular oxygen
FvH4_2g34170	Fvb2: 25445418-25447722	65,767	16,595	−1.987	−4.373	5.000E−05	2.218E−03	subtilisin-like protease	P:GO:0006508; F.GO:0004252	P:proteolysis; F:serine-type endopeptidase activity
FvH4_6g41050	Fvb6: 32391614-32398766	27,190	6,817	−1.996	−2.584	3.750E−03	5.000E−02	chlorophyll a-b binding protein 151, chloroplastic-like, partial	P:GO:0009765; C;GO:0016020	P:photosynthesis, light harvesting; C:membrane
FvH4_3g09680	Fvb3: 5629058-5631096	478,745	119,754	−1.999	−4.187	5.000E−05	2.218E−03	photosystem I reaction center subunit psaK, chloroplastic	P:GO:0015979; C:GO:0016020; C:GO:0009522; F:GO:0016168	P:photosynthesis; C:membrane; C:photosystem I; F:chlorophyll binding
FvH4_6g44990	Fvb6: 34565510-34570206	81,623	20,284	−2.009	−4.342	5.000E−05	2.218E−03	probable indole-3-acetic acid-amido synthetase GH3.5		
FvH4_6g13610	Fvb6: 8271683-8274190	60,969	15,107	−2.013	−4.146	5.000E−05	2.218E−03	expansin-A6	P:GO:0009664; C:GO:0005576	P:plant-type cell wall organization; C:extracellular region
FvH4_6g43040	Fvb6: 33359479-33361283	20,648	5,105	−2.016	−2.601	2.450E−03	3.763E−02	uncharacterized protein		
FvH4_6g40150	Fvb6: 31710858-31712682	2119,710	521,867	−2.022	−2.717	7.500E−04	1.786E−02	chlorophyll a-b binding protein 8, chloroplastic	P:GO:0009765; C:GO:0016020	P:photosynthesis, light harvesting; C:membrane
FvH4_1g03510	Fvb1: 1918189-1923619	21,235	5,210	−2.027	−2.819	2.150E−03	3.425E−02	adenine nucleotide transporter BT1, chloroplastic/mitochondrial	P:GO:0055085	P:transmembrane transport
FvH4_2g12490	Fvb2: 10943985-10944738	499,987	122,428	−2.030	−4.475	5.000E−05	2.218E−03	ATP synthase delta chain, chloroplastic	P:GO:0015986; C:GO:0016020; F:GO:0046933	P:ATP synthesis coupled proton transport; C:membrane; F:proton-transporting ATP synthase activity, rotational mechanism
FvH4_6g11490	Fvb6: 6889482-6906811	53,736	13,132	−2.033	−4.409	5.000E−05	2.218E−03	vacuolar cation/proton exchanger 3-like	P:GO:0006816; P:GO:0006812; P:GO:0055085; C:GO:0016021; F:GO:0015369; F:GO:0008324	P:calcium ion transport; P:cation transport; P:transmembrane transport; C:integral component of membrane; F:calcium:proton antiporter activity; F:cation transmembrane transporter activity
FvH4_1g18970	Fvb1: 11247879-11253086	137,614	33,575	−2.035	−4.419	5.000E−05	2.218E−03	uncharacterized protein, Glycine-rich domain-containing protein-like		
FvH4_4g31140	Fvb4: 30415509-30417353	467,185	113,217	−2.045	−4.121	5.000E−05	2.218E−03	expansin-A1	P:GO:0009664; C:GO:0005576	P:plant-type cell wall organization; C:extracellular region
FvH4_5g14010	Fvb5: 7931662-7935314	37,567	9,088	−2.047	−3.944	5.000E−05	2.218E−03	flavonoid 3'-monooxygenase	P:GO:0055114; F:GO:0020037;	P:oxidation-reduction process; F:heme binding; F:iron ion binding; F:oxidoreductase activity, acting on paired donors, with incorporation or reduction of molecular oxygen

									F:GO:0005506; F:GO:0016705	
FvH4_2g26970	Fvb2: 21549577-21552377	384,594	92,840	−2.051	−4.258	5.000E−05	2.218E−03	photosystem II 22 kDa protein, chloroplastic		
FvH4_3g41620	Fvb3: 34939645-34940283	2979,760	714,865	−2.059	−3.428	5.000E−05	2.218E−03	photosystem I reaction center subunit V, chloroplastic	P:GO:0015979; C:GO:0016020; C:GO:0009522; F:GO:0016168	P:photosynthesis; C:membrane; C:photosystem I; F:chlorophyll binding
FvH4_1g25290	Fvb1: 17074759-17076830	26,520	6,315	−2.070	−3.111	3.500E−04	1.016E−02	uncharacterized protein		
FvH4_3g01050	Fvb3: 527569-528823	26,511	6,287	−2.076	−2.705	1.600E−03	2.845E−02	mavicyanin-like	F:GO:0009055	F:electron transfer activity
FvH4_3g33490	Fvb3: 28889620-28892844	13,585	3,165	−2.102	−2.632	3.450E−03	4.738E−02	thiamine thiazole synthase, chloroplastic-like	P:GO:0006950; P:GO:0009228	P:response to stress; P:thiamine biosynthetic process
FvH4_4g27370	Fvb4: 28396859-28400285	170,794	39,787	−2.102	−4.601	5.000E−05	2.218E−03	GDP-L-galactose phosphorylase 2	F:GO:0080048	F:GDP-D-glucose phosphorylase activity
FvH4_4g32720	Fvb4: 31275531-31276123	102,686	23,898	−2.103	−3.062	4.500E−04	1.218E−02	major latex protein, MLP-like protein 43	P:GO:0006952; P:GO:0009607	P:defense response; P:response to biotic stimulus
FvH4_4g27420	Fvb4: 28425590-28427377	190,634	43,843	−2.120	−4.717	5.000E−05	2.218E−03	3-ketoacyl-CoA synthase 6	P:GO:0006633; P:GO:0008152; C:GO:0016020; F:GO:0003824; F:GO:0016747	P:fatty acid biosynthetic process; P:metabolic process; C:membrane; F:catalytic activity; F:transferase activity, transferring acyl groups other than amino-acyl groups
FvH4_7g27850	Fvb7: 20731673-20732255	453,959	104,226	−2.123	−4.701	5.000E−05	2.218E−03	pectinesterase inhibitor domain, 21 kDa protein	F:GO:0004857	F:enzyme inhibitor activity
FvH4_1g08270	Fvb1: 4379754-4380126	2714,430	619,244	−2.132	−4.562	5.000E−05	2.218E−03	photosystem II, uncharacterized protein	P:GO:0015979; C:GO:0016020; C:GO:0009523	P:photosynthesis; C:membrane; C:photosystem II
FvH4_3g00160	Fvb3: 91235-99400	41,958	9,474	−2.147	−4.515	5.000E−05	2.218E−03	probable galactinol--sucrose galactosyltransferase 2	P:GO:0006508; F:GO:0003824; F:GO:0004252	P:proteolysis; F:catalytic activity; F:serine-type endopeptidase activity
FvH4_6g27700	Fvb6: 21360836-21365897	48,846	11,027	−2.147	−3.993	5.000E−05	2.218E−03	DnaJ homolog subfamily C member 2	F:GO:0009055; F:GO:0005506	F:electron transfer activity; F:iron ion binding
FvH4_1g17940	Fvb1: 10425761-10432019	16,680	3,761	−2.149	−2.996	1.250E−03	2.433E−02	galactomannan galactosyltransferase 1-like	C:GO:0016021; F:GO:0016757	C:integral component of membrane; F:transferase activity, transferring glycosyl groups
FvH4_4g21340	Fvb4: 24380885-24383481	79,787	17,922	−2.154	−4.713	5.000E−05	2.218E−03	S-adenosylmethionine synthase 2	P:GO:0006556; F:GO:0005524; F:GO:0004478	P:S-adenosylmethionine biosynthetic process; F:ATP binding; F:methionine adenosyltransferase activity
FvH4_7g13860	Fvb7: 12351610-12353659	51,137	11,411	−2.164	−3.745	5.000E−05	2.218E−03	remorin-like		
FvH4_6g27920	Fvb6: 21489410-21493992	47,414	10,567	−2.166	−3.941	5.000E−05	2.218E−03	ACT domain-containing protein ACR11		

FvH4_4g13360	Fvb4: 16972557-16974936	48,243	10,740	−2.167	−4.247	5.000E−05	2.218E−03	protein ECERIFERUM 26-like	F:GO:0016747	F:transferase activity, transferring acyl groups other than amino-acyl groups
FvH4_2g31210	Fvb2: 23984136-23987486	1602,480	355,155	−2.174	−3.035	5.000E−05	2.218E−03	photosystem II PsbX	P:GO:0015979; C:GO:0016020; C:GO:0009523	P:photosynthesis; C:membrane; C:photosystem II
FvH4_4g04010	Fvb4: 3468654-3474141	145,836	32,156	−2.181	−4.746	5.000E−05	2.218E−03	probable plastid-lipid-associated protein 6, chloroplastic		
FvH4_2g18090	Fvb2: 15486281-15487211	80,104	17,527	−2.192	−3.972	5.000E−05	2.218E−03	uncharacterized protein		
FvH4_6g53560	Fvb6: 39232986-39237091	17,658	3,776	−2.225	−3.050	7.000E−04	1.719E−02	ribonucleoside-diphosphate reductase small chain	P:GO:0055114	P:oxidation-reduction process
FvH4_4g05760	Fvb4: 5132055-5136676	68,184	14,506	−2.233	−4.728	5.000E−05	2.218E−03	probable pectate lyase 8		
FvH4_6g09970	Fvb6: 5915102-5916203	1131,000	239,870	−2.237	−3.981	5.000E−05	2.218E−03	lipid transfer protein 4	P:GO:0006869; F:GO:0008289	P:lipid transport; F:lipid binding
FvH4_7g01160	Fvb7: 1568330-1574452	47,990	9,615	−2.319	−4.726	5.000E−05	2.218E−03	polyketide synthase 1	P:GO:0009058; P:GO:0008152; F:GO:0003824; F:GO:0016746; F:GO:0016747	P:biosynthetic process; P:metabolic process; F:catalytic activity; F:transferase activity, transferring acyl groups; F:transferase activity, transferring acyl groups other than amino-acyl groups
FvH4_6g00660	Fvb6: 378744-381847	13,453	2,695	−2.320	−2.968	1.950E−03	3.236E−02	putative auxin efflux carrier component 8	P:GO:0055085; C:GO:0016021	P:transmembrane transport; C:integral component of membrane
FvH4_3g06120	Fvb3: 3521880-3529614	4407,370	870,799	−2.340	−2.575	2.250E−03	3.537E−02	chlorophyll a-b binding protein of LHCII type 1-like	P:GO:0009765; C:GO:0016020	P:photosynthesis, light harvesting; C:membrane
FvH4_2g37190	Fvb2: 27124459-27147571	28,328	5,586	−2.342	−2.735	9.000E−04	2.034E−02	pheophytinase, chloroplastic		
FvH4_6g48210	Fvb6: 36358898-36360920	27,384	5,291	−2.372	−3.523	5.000E−05	2.218E−03	expansin-A1	P:GO:0009664; C:GO:0005576	P:plant-type cell wall organization; C:extracellular region
FvH4_1g01190	Fvb1: 601402-604802	14,368	2,770	−2.375	−2.885	2.550E−03	3.857E−02	uncharacterized protein		
FvH4_1g01030	Fvb1: 507447-511071	38,058	7,317	−2.379	−4.317	5.000E−05	2.218E−03	omega-6 fatty acid desaturase, chloroplastic	P:GO:0006629	P:lipid metabolic process
FvH4_1g21450	Fvb1: 13385262-13388290	127,373	24,316	−2.389	−5.288	5.000E−05	2.218E−03	inositol-3-phosphate synthase	P:GO:0006021; P:GO:0008654; F:GO:0004512	P:inositol biosynthetic process; P:phospholipid biosynthetic process; F:inositol-3-phosphate synthase activity
FvH4_5g30940	Fvb5: 21867161-21868613	1355,070	256,749	−2.400	−3.865	5.000E−05	2.218E−03	chlorophyll a-b binding protein CP24 10A, chloroplastic	P:GO:0009765; C:GO:0016020	P:photosynthesis, light harvesting; C:membrane
FvH4_7g31450	Fvb7: 22725705-22729890	25,181	4,715	−2.417	−4.233	5.000E−05	2.218E−03	starch synthase 1, chloroplastic/amyloplastic	F:GO:0004373	F:glycogen (starch) synthase activity
FvH4_3g45570	Fvb3: 37769055-37770050	212,153	39,538	−2.424	−4.593	5.000E−05	2.218E−03	uncharacterized protein		

FvH4_3g44810	Fvb3: 37292068-37293010	39,364	7,316	−2.428	−3.251	7.000E−04	1.719E−02	transmembrane protein 45B		
FvH4_4g33020	Fvb4: 31393766-31402067	15,256	2,831	−2.430	−3.450	4.000E−04	1.118E−02	purple acid phosphatase 8-like	F:GO:0016787	F:hydrolase activity
FvH4_6g32640	Fvb6: 25666200-25673569	24,277	4,485	−2.436	−4.664	5.000E−05	2.218E−03	GDSL esterase/lipase APG	F:GO:0016788	F:hydrolase activity, acting on ester bonds
FvH4_7g25860	Fvb7: 19688928-19690653	339,000	62,579	−2.438	−5.194	5.000E−05	2.218E−03	expansin-A10	P:GO:0009664; C:GO:0005576	P:plant-type cell wall organization; C:extracellular region
FvH4_4g32760	Fvb4: 31283648-31284184	244,399	44,378	−2.461	−4.257	5.000E−05	2.218E−03	kirola-like	P:GO:0006952; P:GO:0009607	P:defense response; P:response to biotic stimulus
FvH4_6g38450	Fvb6: 30386770-30387574	1474,210	267,520	−2.462	−4.545	5.000E−05	2.218E−03	chlorophyll a-b binding protein of LHCII type 1-like	P:GO:0009765; C:GO:0016020	P:photosynthesis, light harvesting; C:membrane
FvH4_5g02340	Fvb5: 1431485-1433512	25,361	4,593	−2.465	−3.313	4.000E−04	1.118E−02	tubulin beta chain-like	P:GO:0007017; C:GO:0005874; F:GO:0005525; F:GO:0003924; F:GO:0005200	P:microtubule-based process; C:microtubule; F:GTP binding; F:GTPase activity; F:structural constituent of cytoskeleton
FvH4_3g40360	Fvb3: 34066985-34068488	79,083	14,317	−2.466	−4.669	5.000E−05	2.218E−03	expansin-A4-like	P:GO:0009664; C:GO:0005576	P:plant-type cell wall organization; C:extracellular region
FvH4_5g31830	Fvb5: 22956758-22961240	63,738	11,538	−2.466	−5.027	5.000E−05	2.218E−03	protein NRT1/ PTR FAMILY 5.10-like	P:GO:0006857; P:GO:0055085; C:GO:0016020; C:GO:0016020; F:GO:0022857; F:GO:0005215	P:oligopeptide transport; P:transmembrane transport; C:membrane; C:membrane; F:transmembrane transporter activity; F:transporter activity
FvH4_3g02980	Fvb3: 1583763-1588587	56,209	10,093	−2.477	−4.446	5.000E−05	2.218E−03	anthocyanidin reductase, flavonoid biosynthesis	F:GO:0003824; F:GO:0050662	F:catalytic activity; F:coenzyme binding
FvH4_3g04660	Fvb3: 2679287-2681111	15,649	2,787	−2.489	−3.162	8.500E−04	1.951E−02	polyphenol oxidase, chloroplastic-like	P:GO:0008152; P:GO:0055114; P:GO:0046148; F:GO:0004097; F:GO:0016491	P:metabolic process; P:oxidation-reduction process; P:pigment biosynthetic process; F:catechol oxidase activity; F:oxidoreductase activity
FvH4_3g37660	Fvb3: 32272449-32273253	2072,090	364,285	−2.508	−4.259	5.000E−05	2.218E−03	chlorophyll a-b binding protein of LHCII type 1-like	P:GO:0009765; C:GO:0016020	P:photosynthesis, light harvesting; C:membrane
FvH4_7g24350	Fvb7: 18809164-18811045	102,342	17,846	−2.520	−4.895	5.000E−05	2.218E−03	chlorophyll a-b binding protein CP29.3, chloroplastic isoform X1	P:GO:0009765; C:GO:0016020	P:photosynthesis, light harvesting; C:membrane
FvH4_5g06720	Fvb5: 3962325-3964815	147,484	25,623	−2.525	−5.502	5.000E−05	2.218E−03	probable pectate lyase 18		
FvH4_6g40970	Fvb6: 32372483-32373647	5710,770	946,875	−2.592	−2.381	2.000E−03	3.296E−02	chlorophyll a-b binding protein 151, chloroplastic	P:GO:0009765; C:GO:0016020	P:photosynthesis, light harvesting; C:membrane

FvH4_3g29540	Fvb3: 22573086-22574827	152,699	25,102	−2.605	−4.955	5.000E−05	2.218E−03	major latex protein 146-like	P:GO:0006952; P:GO:0009607	P:defense response; P:response to biotic stimulus
FvH4_4g28630	Fvb4: 29206860-29210026	21,126	3,434	−2.621	−3.126	1.000E−03	2.150E−02	protein strictosiding synthase-like 10-like	P:GO:0009058; F:GO:0016844	P:biosynthetic process; F:strictosidine synthase activity
FvH4_6g34740	Fvb6: 27411186-27411858	141,138	22,417	−2.654	−4.616	5.000E−05	2.218E−03	auxin-binding protein ABP19a	F:GO:0030145; F:GO:0045735	F:manganese ion binding; F:nutrient reservoir activity
FvH4_3g18760	Fvb3: 12044361-12050554	28,434	4,495	−2.661	−3.771	1.500E−04	5.311E−03	uncharacterized protein	P:GO:0055085; C:GO:0016021; F:GO:0005509	P:transmembrane transport; C:integral component of membrane; F:calcium ion binding
FvH4_2g02070	Fvb2: 1735923-1736342	303,847	47,740	−2.670	−2.896	3.750E−03	5.000E−02	uncharacterized protein		
FvH4_6g38390	Fvb6: 30344332-30345143	551,786	81,859	−2.753	−5.908	5.000E−05	2.218E−03	chlorophyll a-b binding protein of LHCII type 1	P:GO:0009765; C:GO:0016020	P:photosynthesis, light harvesting; C:membrane
FvH4_2g36250	Fvb2: 26568941-26584896	739,553	107,545	−2.782	−4.673	5.000E−05	2.218E−03	dehydration-responsive protein RD22		
FvH4_6g31200	Fvb6: 24362871-24363808	96,333	13,848	−2.798	−3.328	2.650E−03	3.973E−02	uncharacterized protein		
FvH4_2g28920	Fvb2: 22545044-22545446	334,465	46,767	−2.838	−4.707	5.000E−05	2.218E−03	14 kDa proline-rich protein DC2.15-like		
FvH4_3g21020	Fvb3: 14037513-14039386	747,443	85,484	−3.128	−5.578	5.000E−05	2.218E−03	chlorophyll a-b binding protein 13, chloroplastic	P:GO:0009765; C:GO:0016020	P:photosynthesis, light harvesting; C:membrane
FvH4_6g07830	Fvb6: 4683630-4684110	952,647	100,539	−3.244	−6.674	5.000E−05	2.218E−03	uncharacterized protein		
FvH4_3g36020	Fvb3: 30924788-30925623	82,786	8,180	−3.339	−3.518	1.850E−03	3.127E−02	metallothionein-like protein 1	F:GO:0046872	F:metal ion binding
FvH4_6g02980	Fvb6: 1661812-1665311	104,340	10,113	−3.367	−6.584	5.000E−05	2.218E−03	GDP-mannose 3,5-epimerase 2	F:GO:0003824; F:GO:0050662	F:catalytic activity; F:coenzyme binding
FvH4_3g38530	Fvb3: 32875977-32880278	21,069	1,986	−3.407	−4.220	1.000E−04	3.925E−03	vacuolar cation/proton exchanger 3-like	P:GO:0006816; P:GO:0006812; P:GO:0055085; C:GO:0016021; F:GO:0015369; F:GO:0008324	P:calcium ion transport; P:cation transport; P:transmembrane transport; C:integral component of membrane; F:calcium:proton antiporter activity; F:cation transmembrane transporter activity
FvH4_1g13440	Fvb1: 7384238-7387179	53,791	4,841	−3.474	−5.479	5.000E−05	2.218E−03	uncharacterized protein	C:GO:0016021	C:integral component of membrane
FvH4_7g31750	Fvb7: 22913712-22918538	139,104	12,082	−3.525	−7.036	5.000E−05	2.218E−03	probable plastid-lipid-associated protein 14, chloroplastic	P:GO:0006468; F:GO:0005524; F:GO:0004672	P:protein phosphorylation; F:ATP binding; F:protein kinase activity
FvH4_1g04050	Fvb1: 2144949-2145177	5119,150	410,760	−3.640	−6.364	5.000E−05	2.218E−03	uncharacterized protein		

FvH4_3g45290	Fvb3: 37607197-37609278	74,017	5,672	−3.706	−6.293	5.000E−05	2.218E−03	probable polyamine transporter	P:GO:0003333; C:GO:0016020; F:GO:0015171	P:amino acid transmembrane transport; C:membrane; F:amino acid transmembrane transporter activity
FvH4_2g11650	Fvb2: 10174570-10176151	387,149	25,530	−3.923	−7.314	5.000E−05	2.218E−03	transcription factor ORG2-like	P:GO:0006357; F:GO:0003677; F:GO:0046983	P:regulation of transcription from RNA polymerase II promoter; F:DNA binding; F:protein dimerization activity
FvH4_6g45730	Fvb6: 35028795-35031791	69,179	4,561	−3.923	−5.631	5.000E−05	2.218E−03	proline dehydrogenase 2, mitochondrial	P:GO:0006562; F.GO:0004657	P:proline catabolic process; F:proline dehydrogenase activity
FvH4_1g12090	Fvb1: 6609415-6610712	144,282	9,045	−3.996	−6.505	5.000E−05	2.218E−03	Glyoxalase/fosfomycin resistance/dioxygenase domain		
FvH4_1g04060	Fvb1: 2147352-2147541	2857,810	150,920	−4.243	−11.524	1.150E−03	2.326E−02			
FvH4_3g37570	Fvb3: 32218393-32220360	54,210	1,687	−5.006	−4.920	7.000E−04	1.719E−02	uncharacterized protein		
FvH4_3g36040	Fvb3: 30928874-30931002	142,261	3,110	−5.516	−7.193	5.000E−05	2.218E−03	uncharacterized protein		

[1] In GO IDs and GO names columns, F: molecular function, C: cellular component and P: biological process.

© 2020 by the authors. Licensee MDPI, Basel, Switzerland. This article is an open access article distributed under the terms and conditions of the Creative Commons Attribution (CC BY) license (http://creativecommons.org/licenses/by/4.0/).
